# Supplementary material for: Dear student, what should I write on my wall? A case study on academic uses of Facebook and Instagram during the pandemic
Source: PLoS One. 2021 Sep 23;16(9):e0257729. doi: 10.1371/journal.pone.0257729 (PMC8459956; doi:10.1371/journal.pone.0257729)
Supplement: S1 Appendix — (DOCX) [file pone.0257729.s001.docx]

**S1 Appendix A. Initial versions of the scales**

**Table A1. The use of Facebook as a tool in the educational process by teachers**

| Construct | Variables | Survey items  Please indicate how much you agree or disagree with the following statements… seven-point Likert scale, ranging from 1 (“disagree very strongly”) to 7 (“agree very strongly”) |
| --- | --- | --- |
| Didactic activity (AD_Fp) | f1.p | Facebook is an appropriate platform for teachers to post information/article/video regarding the course/seminar topics |
|  | f2.p | Facebook is an appropriate platform for teachers to post links regarding the topics of the course/seminar |
|  | f3.p | Facebook is an appropriate platform for teachers to offer answers to students’ questions about the tasks/projects that they have to carry out |
|  | f4.p. | Facebook is an appropriate platform for teachers to invite field specialists to debate certain topics |
|  | f5.p | Facebook is an appropriate platform for teachers to propose various debate topics regarding the theme of the course/seminar |
|  | f6.p | Facebook is an appropriate platform for teachers to share various experiences with respect to their didactic activity |
|  | f7.p | Facebook is an appropriate platform for teachers to carry out surveys on certain topics related to the course/seminar |
|  | f8.p | Facebook is an appropriate platform for teachers to implement a teaching model that is focused on students’ needs |
|  | f16.p | Facebook is an appropriate platform for teachers to give feedback on certain course/seminar activities carried out by students |
|  | f17.p | Facebook is an appropriate platform for teachers to give feedback on certain projects before the students have to hand them in |
|  | f18.p | Facebook is an appropriate platform for teachers to chat with student about the result of their evaluation |
|  | f19.p | Facebook is an appropriate platform for teachers to share with students information regarding organizational and administrative aspects of the faculty |
|  | f22.p | Facebook is an appropriate platform for teachers to announce changes regarding the courses/deadlines |
|  | f23.p | Facebook is an appropriate platform for teachers to communicate with students about certain personal problems that have an impact on their academic performance |
|  | f24.p | Facebook is an appropriate platform for teachers to manage o series of situation that requires group interaction and consensus |
| Extracurricular information and  career development (EC_Fp) | f9.p | Facebook is an appropriate platform for teachers to post announcement of jobs that might be of interest to students |
|  | f10.p. | Facebook is an appropriate platform for teachers to post announcements about internship opportunities |
|  | f11.p | Facebook is an appropriate platform for teachers to post announcements about personal development workshops |
|  | f12.p | Facebook is an appropriate platform for teachers to post announcements about volunteering opportunities |
|  | f13.p | Facebook is an appropriate platform for teachers to post announcements about various project of interest at the community level |
|  | f14.p | Facebook is an appropriate platform for teachers to post announcements about various partnerships tha the faculty has |
|  | f15.p | Facebook is an appropriate platform for teachers to post announcements about opportunities to develop the internship required by the faculty |
|  | f20.p | Facebook in an appropriate platform for teachers to maintain connection with students |
|  | f21.p | Facebook is an appropriate platform for teachers to maintain connection with graduates |
|  | f25.p | Facebook is an appropriate platform for teachers to positively influence students through personal example by posting information about the activities they carry out |
|  | f26.p | Facebook is an appropriate platform for teachers to share scientific information outside of the course/seminar curriculum |
|  | f27.p | Facebook is an appropriate platform for teachers to post information about the projects they are involved in |
|  | f28.p | Facebook is an appropriate platform for teachers to post information about their recent publications |
|  | f29.p | Facebook is an appropriate platform for teachers to post information about conferences of scientific interest |
|  | f30.p | Facebook is an appropriate platform for teachers to post information about workshops of scientific interest |
|  | f31.p | Facebook is an appropriate platform for teachers to test research instruments |
|  | f32.p | Facebook is an appropriate platform for teachers to test research ideas |
|  | f33.p | Facebook is an appropriate platform for teachers to post information aimed at promoting continuing study programs (master, PhD, etc.) |
|  | f34.p | Facebook is an appropriate platform for teachers to post information aimed at promoting socio-cultural events that are carried out/will be carried out within their faculty |
|  | f35.p. | Facebook is an appropriate platform for teachers to post information about other extracurricular events that are carried out/will be carried out within their faculty |

**Table A2. The use of Facebook as a tool in the educational process by students**

| Construct | Variables | Survey items  Please indicate how much you agree or disagree with the following statements… seven-point Likert scale, ranging from 1 (“disagree very strongly”) to 7 (“agree very strongly”) |
| --- | --- | --- |
| Didactic activity (AD_Fs) | f1.s | Facebook is an appropriate platform for students to post information/articles/videos regarding the course/seminar topics |
|  | f2.s | Facebook is an appropriate platform for students to post links regarding the topics of the course/seminar |
|  | f3.s | Facebook is an appropriate platform for students to post questions about the tasks/projects that they have to carry out |
|  | f4.s | Facebook is an appropriate platform for students to post tasks/projects/ essays they have done |
|  | f5.s | Facebook is an appropriate platform for students to collaborate on carrying out various projects/task/essays for the seminar activity |
|  | f6.s | Facebook is an appropriate platform for students to propose varied debate topics regarding the theme of the course/seminar. |
|  | f7.s | Facebook is an appropriate platform for students to share experiences related to the didactic activity |
|  | f8.s | Facebook is an appropriate platform for students to share various ideas regarding the didactic activity |
|  | f9.s | Facebook is an appropriate platform for students to carry out surveys on certain topics related to the course/seminar |
|  | f15.s | Facebook is an appropriate platform for students to post feedback about the course/seminar activity |
|  | f16.s | Facebook is an appropriate platform for students to receive feedback from their peers on their essays/projects |
|  | f18.s | Facebook is an appropriate platform for students to post announcements about changes related to the course, deadlines, etc. |
|  | f19.s | Facebook is an appropriate platform for students to manage a series of situations that require interaction and group consensus |
|  | f21.s | Facebook is an appropriate platform for students to post information about the projects they are involved in |
| Extracurricular information and  career development (EC_Fs) | f10.s | Facebook is an appropriate platform for students to post announcements about jobs of interest for other students |
|  | f11.s | Facebook is an appropriate platform for students to post announcements about internship opportunities |
|  | f12.s | Facebook is an appropriate platform for students to post announcements about personal development workshops |
|  | f13.s | Facebook is an appropriate platform for students to post announcements about volunteering opportunities |
|  | f14.s | Facebook is an appropriate platform for students to post announcements about various projects on interest at the community level |
|  | f17.s | Facebook is an appropriate platform for students to keep in touch with other students |
|  | f20.s | Facebook is an appropriate platform for students to share extracurricular information |
|  | f22.s | Facebook is an appropriate platform for students to post information about a series of students specific scientific conferences/ sessions |
|  | f23.s | Facebook is an appropriate platform for students to post information about workshops of scientific interest |
|  | f24.s | Facebook is an appropriate platform for students to post information aimed at promoting continuing study programs (master, PhD) |
|  | f25.s | Facebook is an appropriate platform for students to post information aimed at promoting socio-cultural events that are carried out/will be carried out within their faculty |
|  | f26.s | Facebook is an appropriate platform for students to post information about other extracuricular events that are carried out/will be carried out within their faculty |

**Table A3.The use of Instagram as a tool in the educational process by teachers**

| Construct | Variables | Survey items  Please indicate how much you agree or disagree with the following statements… seven-point Likert scale, ranging from 1 (“disagree very strongly”) to 7 (“agree very strongly”) |
| --- | --- | --- |
| Didactic activity (AD_Ip) | i1.p | Instagram is an appropriate platform for teachers to post photos/various types of presentation/videos related to the topic of the course/seminar |
|  | i2.p. | Instagram is an appropriate platform for teachers to invite field specialists to post photos/various types of presentations related to the course/seminar topic |
|  | i3.p | Instagram is an appropriate platform for teachers to propose debate topics starting from photos/ various types of presentations/videos related to the course/seminar topic |
|  | i4.p | Instagram is an appropriate platform for teachers to share personal experiences related to their didactic activity |
|  | i5.p | Instagram is an appropriate platform for teachers to propose ideas regarding the didactic activity |
|  | i6.p | Instagram is an appropriate platform for teachers to post photos, types of presentation, videos developed during the course/seminar |
|  | i7.p | Instagram is an appropriate platform for teachers to post photos/ various types of presentations/videos developed during certain extracurricular activities |
|  |  |  |
|  | i16.p. | Instagram is an appropriate platform for teachers to share with students information regarding organizational or administrative aspects of the faculty |
|  | i19.p | Instagram is an appropriate platform for teachers to announce changes regarding the course, deadlines, etc. |
| Extracurricular information and  career development (EC_Ip) | i8.p | Instagram is an appropriate platform for teachers to post photos/various types of presentations/videos developed together with other students during certain extracurricular activities |
|  | i9.p | Instagram is an appropriate platform for teachers to post announcements about jobs of interest for students |
|  | i10.p | Instagram is an appropriate platform for teachers to post announcements about internship opportunities |
|  | i11.p | Instagram is an appropriate platform for teachers to post announcements about personal development workshops |
|  | i12.p | Instagram is an appropriate platform for teachers to post announcements about volunteering opportunities |
|  | i13.p | Instagram is an appropriate platform for teachers to post announcements about various projects of interest at the community level |
|  | i14.p | Instagram is an appropriate platform for teachers to post announcements about various partnerships of the faculty |
|  | i15.p | Instagram is an appropriate platform for teachers to post announcement about opportunities to develop the internship required by the faculty |
|  | i17.p | Instagram is an appropriate platform for teachers to maintain connection with students |
|  | i18.p | Instagram is an appropriate platform for teachers to maintain connesction with graduates |
|  | i20.p | Instagram is an appropriate platform for teachers to positively influence students through personal example by posting information about the activities they carry out |
|  | i21.p | Instagram is an appropriate platform for teachers to share scientific information outside of the course/seminar curriculum |
|  | i22.p | Instagram is an appropriate platform for teachers to post information about the project they are involved in |
|  | i23.p | Instagram is an appropriate platform for teachers to post information about their recent publications |
|  | i24.p | Instagram is an appropriate platform for teachers to post information about conferences of scientific interest |
|  | i25.p | Instagram is an appropriate platform for teachers to post information about workshops of interest in the study field |
|  | i26.p | Instagram is an appropriate platform for teachers to post information aimed at promoting continuing study programs (master, PhD) |
|  | i27.p | Instagram is an appropriate platform for teachers to post information aimed at promoting socio-cultural events that are carried out/will be carried out within their faculty |
|  | i28.p | Instagram is an appropriate platform for teachers to post information about other extracurricular events that are carried out/will be carried out within their faculty |

**Table A4. The use of Instagram as a tool in the educational process by students**

| Construct | Variables | Survey items  Please indicate how much you agree or disagree with the following statements… seven-point Likert scale, ranging from 1 (“disagree very strongly”) to 7 (“agree very strongly”) |
| --- | --- | --- |
| Didactic activity (AD_Is) | i1.s. | Instagram is an appropriate platform for students to post photos/various types of presentation/videos related to the topic of the course/seminar |
|  | i2.s. | Instagram is an appropriate platform for students to collaborate with their peers on carrying out various projects/task/essays for the seminar activity |
|  | i3.s. | Instagram is an appropriate platform for students to propose varied debate topics regarding the theme of the course/seminar. |
|  | i4.s. | Instagram is an appropriate platform for students to share various experiences with respect to their didactic activity |
|  | i5.s. | Instagram is an appropriate platform for students to share various ideas with respect to their didactic activity |
|  | i11.s. | Instagram is an appropriate platform for students to post feedback about the course/seminar activity |
|  | i12.s. | Instagram is an appropriate platform for students to receive feedback from their peers on their essays/projects |
|  | i14.s. | Instagram is an appropriate platform for students to post announcements about changes related to the course, deadlines, etc. |
|  | i15.s. | Instagram an appropriate platform for students to manage situations that requires interaction and group consensus |
|  | i17.s. | Instagram is an appropriate platform for students to post information about the projects they are involved in |
| Extracurricular information and  career development (EC_Is) | i6.s. | Instagram is an appropriate platform for students to post announcements about jobs of interest for other students |
|  | i7.s. | Instagram is an appropriate platform for students to post announcements about internship opportunities |
|  | i8.s. | Instagram is an appropriate platform for students to post announcements about personal development workshops |
|  | i9.s. | Instagram is an appropriate platform for students to post announcements about volunteering opportunities |
|  | i10.s. | Instagram is an appropriate platform for students to post announcements about various projects on interest at the community level |
|  | i13.s. | Instagram is an appropriate platform for students to keep in touch with other students |
|  | i16.s. | Instagram is an appropriate platform for students to share extracurricular information |
|  |  |  |
|  | i18.s. | Instagram Facebook is an appropriate platform for students to post information about a series of students specific scientific conferences/ sessions |
|  | i19.s. | Instagram is an appropriate platform for students to post information about workshops of scientific interest. |
|  | I20.s. | Instagram is an appropriate platform for students to post information aimed at promoting continuing study programs (master, PhD) |
|  | i21.s. | Instagram is an appropriate platform for students to post information aimed at promoting socio-cultural events that are carried out/will be carried out within their faculty. |
|  | i22.s. | Instagram is an appropriate platform for students to post information about other extracurricular events that are carried out/will be carried out within their faculty |

S1. Do you have a Facebook account?

1. Yes.
2. No

S2. How often do you access your account?

1. Daily
2. 3-4 times a week
3. 1-2 times a week
4. Several times a month
5. Rarely
6. It is not the case/I do not have an account

S3.Do you have an Instagram account?

1. Yes.
2. No

S4. How often do you access your account?

1. Daily
2. 3-4 times a week
3. 1-2 times a week
4. Several times a month
5. Rarely
6. It is not the case/I do not have an account

5S. Gender:

1. Male
2. Female

S6. You are currently:

1. A Bachelor student
2. A Master students
3. A PhD student

S7. What is the field in which you are taking courses?
