# Supplementary material for: Dear student, what should I write on my wall? A case study on academic uses of Facebook and Instagram during the pandemic
Source: PLoS One. 2021 Sep 23;16(9):e0257729. doi: 10.1371/journal.pone.0257729 (PMC8459956; doi:10.1371/journal.pone.0257729)
Supplement: S2 Appendix — (DOCX) [file pone.0257729.s002.docx]

**S2 Appendix B. Exploratory factor analyses item loadings ,Reliability, and Explained Variances**

**Table B1. The use of Facebook as a tool in the educational process by teachers**

| Survey items  Please indicate how much you agree or disagree with the following statements… seven-point Likert scale, ranging from 1 (“disagree very strongly”) to 7 (“agree very strongly”) | M (SD) | Factor  Didactic activity -(AD_Fp) | Factor  Extracurricular information and career development  (EC_Fp) |
| --- | --- | --- | --- |
| f1.p. Facebook is an appropriate platform for teachers to post information/article/video regarding the course/seminar topics | 4.89 (2.20) | .81 |  |
| f2.p. Facebook is an appropriate platform for teachers to post links regarding the topics of the course/seminar | 5.03 (2.16) | .81 |  |
| f3.p. Facebook is an appropriate platform for teachers to offer answers to students’ questions about the tasks/projects that they have to carry out | 5.10 (2.17) | .82 |  |
| f4.p. Facebook is an appropriate platform for teachers to invite field specialists to debate certain topics | 5.23 (1.98) | .67 |  |
| f5.p. Facebook is an appropriate platform for teachers to propose various debate topics regarding the theme of the course/seminar | 5.01 (2.05) | .80 |  |
| f6.p. Facebook is an appropriate platform for teachers to share various experiences with respect to their didactic activity | 5.16 (2.00) | .71 |  |
| f7.p. Facebook is an appropriate platform for teachers to carry out surveys on certain topics related to the course/seminar | 5.37 (2.00) | .67 |  |
| f8.p. Facebook is an appropriate platform for teachers to implement a teaching model that is focused on students’ needs | 5.22 (2.10) | .78 |  |
| f16.p. Facebook is an appropriate platform for teachers to give feedback on certain course/seminar activities carried out by students | 4.76 (2.26) | .84 |  |
| f17.p. Facebook is an appropriate platform for teachers to give feedback on certain projects before the students have to hand them in | 4.82 (2.24) | .87 |  |
| f18.p. Facebook is an appropriate platform for teachers to chat with student about the result of their evaluation | 4.53 (2.31) | .85 |  |
| f19.p. Facebook is an appropriate platform for teachers to share with students information regarding organizational and administrative aspects of the faculty | 5.03 (2.12) | .68 |  |
| f22.p. Facebook is an appropriate platform for teachers to announce changes regarding the courses/deadlines | 5.34 (2.14) | .79 |  |
| f9.p. Facebook is an appropriate platform for teachers to post announcement of jobs that might be of interest to students | 5.81 (1.77) |  | .77 |
| f10.p. Facebook is an appropriate platform for teachers to post announcements about internship opportunities | 5.79 (1.71) |  | .79 |
| f11.p. Facebook is an appropriate platform for teachers to post announcements about personal development workshops | 5.84 (1.70) |  | .81 |
| f12.p. Facebook is an appropriate platform for teachers to post announcements about volunteering opportunities | 5.92 (1.65) |  | .81 |
| f13.p. Facebook is an appropriate platform for teachers to post announcements about various project of interest at the community level | 5.79 (1.69) |  | .83 |
| f14.p. Facebook is an appropriate platform for teachers to post announcements about various partnerships that the faculty has | 5.65 (1.79) |  | .78 |
| f15.p. Facebook is an appropriate platform for teachers to post announcements about opportunities to develop the internship required by the faculty | 5.74 (1.80) |  | .69 |
| f25.p. Facebook is an appropriate platform for teachers to positively influence students through personal example by posting information about the activities they carry out | 5.17 (2.04) |  | .63 |
| f26.p. Facebook is an appropriate platform for teachers to share scientific information outside of the course/seminar curriculum | 5.25 (1.95) |  | .73 |
| f27.p. Facebook is an appropriate platform for teachers to post information about the projects they are involved in | 5.14 (1.96) |  | .76 |
| f28.p. Facebook is an appropriate platform for teachers to post information about their recent publications | 5.26 (1.91) |  | .82 |
| f29.p. Facebook is an appropriate platform for teachers to post information about conferences of scientific interest | 5.34 (1.88) |  | .84 |
| f30.p. Facebook is an appropriate platform for teachers to post information about workshops of scientific interest | 5.48 (1.80) |  | .88 |
| f31.p. Facebook is an appropriate platform for teachers to test research instruments | 5.13 (1.97) |  | .71 |
| f32.p. Facebook is an appropriate platform for teachers to test research ideas | 5.18 (1.95) |  | .73 |
| f33.p. Facebook is an appropriate platform for teachers to post information aimed at promoting continuing study programs (master, PhD, etc.) | 5.46 (1.91) |  | .72 |
| f34.p. Facebook is an appropriate platform for teachers to post information aimed at promoting socio-cultural events that are carried out/will be carried out within their faculty | 5.69 (1.76) |  | .83 |
| f35.p. Facebook is an appropriate platform for teachers to post information about other extracurricular events that are carried out/will be carried out within their faculty | 5.78 (1.71) |  | .85 |
| ***N of items*** |  | ***13*** | ***18*** |
| ***Eigenvalues*** |  | ***13.15*** | ***10.72*** |
| ***% of explained variance*** |  | ***42.44%*** | ***34.59%*** |
| ***Cronbach’s α*** |  | ***0.97*** | ***.98*** |

Note: The Kaiser–Meyer–Olkin (KMO = .97) and Bartlett’s test of sphericity (χ2 = 39931,p < .001); Rotation method: Varimax.

**Table B2. The use of Facebook as a tool in the educational process by students**

| Survey items  Please indicate how much you agree or disagree with the following statements… seven-point Likert scale, ranging from 1 (“disagree very strongly”) to 7 (“agree very strongly”) | M (SD) | Factor  Didactic activity (AD_Fs) | Factor  Extracurricular information and  career development (EC_Fs) |
| --- | --- | --- | --- |
| f1.s. Facebook is an appropriate platform for students to post information/articles/videos regarding the course/seminar topics | 4.62 (2.23 | .84 |  |
| f2.s. Facebook is an appropriate platform for students to post links regarding the topics of the course/seminar | 4.65 (2.22 | .84 |  |
| f3.s. Facebook is an appropriate platform for students to post questions about the tasks/projects that they have to carry out | 4.85 (2.23 | .86 |  |
| f4.s. Facebook is an appropriate platform for students to post tasks/projects/ essays they have done | 4.22 (2.33 | .85 |  |
| f5.s. Facebook is an appropriate platform for students to collaborate on carrying out various projects/task/essays for the seminar activity | 5.05 (2.06) | .72 |  |
| f6.s. Facebook is an appropriate platform for students to propose varied debate topics regarding the theme of the course/seminar. | 4.89 (2.13) | .85 |  |
| f7.s. Facebook is an appropriate platform for students to share experiences related to the didactic activity | 4.86 (2.08) | .79 |  |
| f8.s. Facebook is an appropriate platform for students to share various ideas regarding the didactic activity | 4.97 (2.02) | .80 |  |
| f9.s. Facebook is an appropriate platform for students to carry out surveys on certain topics related to the course/seminar | 5.16 (1.99) | .67 |  |
| f15.s. Facebook is an appropriate platform for students to post feedback about the course/seminar activity | 4.72 (2.22) | .85 |  |
| f16.s. Facebook is an appropriate platform for students to receive feedback from their peers on their essays/projects | 4.57 (2.29) | .83 |  |
| f18.s. Facebook is an appropriate platform for students to post announcements about changes related to the course, deadlines, etc. | 5.19 (2.10) | .70 |  |
| f10.s. Facebook is an appropriate platform for students to post announcements about jobs of interest for other students | 5.62 (1.79) |  | .85 |
| f11.s. Facebook is an appropriate platform for students to post announcements about internship opportunities | 5.57 (1.82) |  | .87 |
| f12.s. Facebook is an appropriate platform for students to post announcements about personal development workshops | 5.61 (1.78) |  | .88 |
| f13.s. Facebook is an appropriate platform for students to post announcements about volunteering opportunities | 5.69 (1.77) |  | .89 |
| f14.s. Facebook is an appropriate platform for students to post announcements about various projects on interest at the community level | 5.62 (1.78) |  | .88 |
| f17.s. Facebook is an appropriate platform for students to keep in touch with other students | 5.85 (1.72) |  | .79 |
| f20.s. Facebook is an appropriate platform for students to share extracurricular information | 5.29 (1.93) |  | .75 |
| f22.s. Facebook is an appropriate platform for students to post information about a series of students specific scientific conferences/ sessions | 5.32 (1.96) |  | .78 |
| f23.s. Facebook is an appropriate platform for students to post information about workshops of scientific interest | 5.35 (1.92) |  | .84 |
| f24.s. Facebook is an appropriate platform for students to post information aimed at promoting continuing study programs (master, PhD) | 5.35 (1.95) |  | .76 |
| f25.s. Facebook is an appropriate platform for students to post information aimed at promoting socio-cultural events that are carried out/will be carried out within their faculty | 5.53 (1.86) |  | .87 |
| f26.s. Facebook is an appropriate platform for students to post information about other extracurricular events that are carried out/will be carried out within their faculty | 5.57 (1.82) |  | .87 |
| ***N of items*** |  | ***12*** | ***12*** |
| ***Eigenvalues*** |  | ***10.28*** | ***9.24*** |
| ***% of explained variance*** |  | ***42.85%*** | ***38.53%*** |
| ***Cronbach’s α*** |  | ***0.97*** | ***.98*** |

Note: The Kaiser–Meyer–Olkin (KMO = .96) and Bartlett’s test of sphericity (χ2 = 32686,p < .001); Rotation method: Varimax.

**Table B3.The use of Instagram as a tool in the educational process by teachers**

| Survey items  Please indicate how much you agree or disagree with the following statements… seven-point Likert scale, ranging from 1 (“disagree very strongly”) to 7 (“agree very strongly”) | M (SD) | Factor  Didactic activity (AD_Ip) | Factor  Extracurricular information and  career development (EC_Ip) |
| --- | --- | --- | --- |
| i1.p. Instagram is an appropriate platform for teachers to post photos/various types of presentation/videos related to the topic of the course/seminar | 4.13 (2.41) | .87 |  |
| i2.p. Instagram is an appropriate platform for teachers to invite field specialists to post photos/various types of presentations related to the course/seminar topic | 4.26 (2.37) | .83 |  |
| i3.p. Instagram is an appropriate platform for teachers to propose debate topics starting from photos/ various types of presentations/videos related to the course/seminar topic | 4.17 (2.38) | .83 |  |
| i4.p. Instagram is an appropriate platform for teachers to share personal experiences related to their didactic activity | 4.28 (2.31) | .78 |  |
| i5.p. Instagram is an appropriate platform for teachers to propose ideas regarding the didactic activity | 4.22 (2.35) | .80 |  |
| i6.p. Instagram is an appropriate platform for teachers to post photos, types of presentation, videos developed during the course/seminar | 4.20 (2.37) | .85 |  |
| i7.p. Instagram is an appropriate platform for teachers to post photos/ various types of presentations/videos developed during certain extracurricular activities | 4.34 (2.32) | .79 |  |
| i8.p. Instagram is an appropriate platform for teachers to post photos/various types of presentations/videos developed together with other students during certain extracurricular activities | 4.52 (2.27) | .67 |  |
| i16.p. Instagram is an appropriate platform for teachers to share with students information regarding organizational or administrative aspects of the faculty | 4.28 (2.33) | .63 |  |
| i19.p. Instagram is an appropriate platform for teachers to announce changes regarding the course, deadlines, etc. | 4.19 (2.42) | .77 |  |
| i9.p. Instagram is an appropriate platform for teachers to post announcements about jobs of interest for students | 4.83 (2.23) |  | .82 |
| i10.p. Instagram is an appropriate platform for teachers to post announcements about internship opportunities | 4.81 (2.23) |  | .85 |
| i11.p. Instagram is an appropriate platform for teachers to post announcements about personal development workshops | 4.81 (2.22) |  | .85 |
| i12.p. Instagram is an appropriate platform for teachers to post announcements about volunteering opportunities | 4.88 (2.20) |  | .86 |
| i13.p. Instagram is an appropriate platform for teachers to post announcements about various projects of interest at the community level | 4.80 (2.22) |  | .86 |
| i14.p. Instagram is an appropriate platform for teachers to post announcements about various partnerships of the faculty | 4.70 (2.24) |  | .83 |
| i15.p. Instagram is an appropriate platform for teachers to post announcement about opportunities to develop the internship required by the faculty | 4.74 (2.26) |  | .79 |
| i20.p. Instagram is an appropriate platform for teachers to positively influence students through personal example by posting information about the activities they carry out | 4.64 (2.28) |  | .62 |
| i21.p. Instagram is an appropriate platform for teachers to share scientific information outside of the course/seminar curriculum | 4.49 (2.29) |  | .71 |
| i22.p. Instagram is an appropriate platform for teachers to post information about the project they are involved in | 4.48 (2.25) |  | .70 |
| i23.p. Instagram is an appropriate platform for teachers to post information about their recent publications | 4.53 (2.23) |  | .73 |
| i24.p. Instagram is an appropriate platform for teachers to post information about conferences of scientific interest | 4.54 (2.25) |  | .78 |
| i25.p. Instagram is an appropriate platform for teachers to post information about workshops of interest in the study field | 4.70 (2.24) |  | .84 |
| i26.p. Instagram is an appropriate platform for teachers to post information aimed at promoting continuing study programs (master, PhD) | 4.69 (2.26) |  | .76 |
| i27.p. Instagram is an appropriate platform for teachers to post information aimed at promoting socio-cultural events that are carried out/will be carried out within their faculty | 4.78 (2.22) |  | .84 |
| i28.p. Instagram is an appropriate platform for teachers to post information about other extracurricular events that are carried out/will be carried out within their faculty | 4.85 (2.22) |  | .85 |
| ***N of items*** |  | ***10*** | ***16*** |
| ***Eigenvalues*** |  | ***12.47*** | ***9.84*** |
| ***% of explained variance*** |  | ***47.99%*** | ***37.87%*** |
| ***Cronbach’s α*** |  | ***0.97*** | ***.98*** |

Note: The Kaiser–Meyer–Olkin (KMO = .98) and Bartlett’s test of sphericity (χ2 = 42437,p < .001); Rotation method: Varimax.

**Table B4. The use of Instagram as a tool in the educational process by students**

| Survey items  Please indicate how much you agree or disagree with the following statements… seven-point Likert scale, ranging from 1 (“disagree very strongly”) to 7 (“agree very strongly”) | M (SD) | Factor  Didactic activity (AD_Is) | Factor  Extracurricular information and  career development (EC_Is) |
| --- | --- | --- | --- |
| i1.s. Instagram is an appropriate platform for students to post photos/various types of presentation/videos related to the topic of the course/seminar | 3.92 (2.37) | .85 |  |
| i2.s. Instagram is an appropriate platform for students to collaborate with their peers on carrying out various projects/task/essays for the seminar activity | 4.14 (2.35) | .80 |  |
| i3.s. Instagram is an appropriate platform for students to propose varied debate topics regarding the theme of the course/seminar. | 3.93 (2.34) | .85 |  |
| i4.s. Instagram is an appropriate platform for students to share various experiences with respect to their didactic activity | 4.07 (2.29) | .83 |  |
| i5.s. Instagram is an appropriate platform for students to share various ideas with respect to their didactic activity | 4.05 (2.29) | .82 |  |
| i11.s. Instagram is an appropriate platform for students to post feedback about the course/seminar activity | 3.89 (2.37) | .84 |  |
| i12.s. Instagram is an appropriate platform for students to receive feedback from their peers on their essays/projects | 3.79 (2.37) | .86 |  |
| i14.s. Instagram is an appropriate platform for students to post announcements about changes related to the course, deadlines, etc. | 4.06 (2.37) | .79 |  |
| i15.s. Instagram an appropriate platform for students to manage situations that requires interaction and group consensus | 4.22 (2.31) | .72 |  |
| i6.s. Instagram is an appropriate platform for students to post announcements about jobs of interest for other students | 4.66 (2.26) |  | .84 |
| i7.s. Instagram is an appropriate platform for students to post announcements about internship opportunities | 4.65 (2.27) |  | .86 |
| i8.s. Instagram is an appropriate platform for students to post announcements about personal development workshops | 4.69 (2.25) |  | .87 |
| i9.s. Instagram is an appropriate platform for students to post announcements about volunteering opportunities | 4.80 (2.26) |  | .86 |
| i10.s. Instagram is an appropriate platform for students to post announcements about various projects on interest at the community level | 4.72 (2.24) |  | .87 |
| i13.s. Instagram is an appropriate platform for students to keep in touch with other students | 4.93 (2.24) |  | .75 |
| i16.s. Instagram is an appropriate platform for students to share extracurricular information | 4.47 (2.26) |  | .75 |
| i18.s. Instagram Facebook is an appropriate platform for students to post information about a series of students specific scientific conferences/ sessions | 4.43 (2.29) |  | .77 |
| i19.s. Instagram is an appropriate platform for students to post information about workshops of scientific interest. | 4.53 (2.26) |  | .81 |
| I20.s. Instagram is an appropriate platform for students to post information aimed at promoting continuing study programs (master, PhD) | 4.46 (2.29) |  | .76 |
| i21.s. Instagram is an appropriate platform for students to post information aimed at promoting socio-cultural events that are carried out/will be carried out within their faculty. | 4.62 (2.26) |  | .83 |
| i22.s. Instagram is an appropriate platform for students to post information about other extracurricular events that are carried out/will be carried out within their faculty | 4.64 (2.26) |  | .84 |
| ***N of items*** |  | ***9*** | ***12*** |
| ***Eigenvalues*** |  | ***9.85*** | ***3.88*** |
| ***% of explained variance*** |  | ***46.93%*** | ***39.93%*** |
| ***Cronbach’s α*** |  | ***0.98*** | ***.98*** |

Note: The Kaiser–Meyer–Olkin (KMO = .96) and Bartlett’s test of sphericity (χ2 = 34415,p < .001); Rotation method: Varimax.
