# Supplementary material for: Dear student, what should I write on my wall? A case study on academic uses of Facebook and Instagram during the pandemic
Source: PLoS One. 2021 Sep 23;16(9):e0257729. doi: 10.1371/journal.pone.0257729 (PMC8459956; doi:10.1371/journal.pone.0257729)
Supplement: S3 Appendix — (DOCX) [file pone.0257729.s003.docx]

**Appendix C.**

**Table A1.** **Significant t-test results: comparisons between index variables**

|  |  |  | | t-test for Equality of Means | | | | | | |
| --- | --- | --- | --- | --- | --- | --- | --- | --- | --- | --- |
| Group | N | Mean | S. D. | t | df | p | Mean Difference | Std. Error Difference | CI4 | |
|  |  |  |  |  |  |  |  |  | Lower | Upper |
| AD_Fp | 872 | 5.03 | 1.87 | 5.66 | 871 | .00 | .22 | .04 | .14 | .30 |
| AD_Fs | 872 | 4.81 | 1.91 |  |  |  |  |  |  |  |
| AD_Fp | 872 | 5.03 | 1.87 | -12.39 | 871 | .00 | -.48 | .03 | -.50 | -.40 |
| EC_FP | 872 | 5.52 | 1.59 |  |  |  |  |  |  |  |
| AD_Fs | 872 | 4.81 | 1.91 | -15.76 | 871 | .00 | -.71 | .04 | -.80 | -.63 |
| EC_Fs | 872 | 5.53 | 1.67 |  |  |  |  |  |  |  |
| AD_Ip | 872 | 4.25 | 2.15 | 6.53 | 871 | .00 | .25 | 0.03 | -.17 | .32 |
| AD_Is | 872 | 4.00 | 2.18 |  |  |  |  |  |  |  |
| AD_Ip | 872 | 4.25 | 2.15 | -12.23 | 871 | .00 | -.44 | .03 | -.51 | -.37 |
| EC_Ip | 872 | 4.7 | 2.07 |  |  |  |  |  |  |  |
| AD_Is | 872 | 4.00 | 2.18 | -14.57 | 871 | .00 | -.62 | 0.04 | -.70 | -.54 |
| EC_Is | 872 | 4.63 | 2.10 |  |  |  |  |  |  |  |
