# Supplementary material for: Dear student, what should I write on my wall? A case study on academic uses of Facebook and Instagram during the pandemic
Source: PLoS One. 2021 Sep 23;16(9):e0257729. doi: 10.1371/journal.pone.0257729 (PMC8459956; doi:10.1371/journal.pone.0257729)
Supplement: S4 Appendix — (DOCX) [file pone.0257729.s004.docx]

S4. Appendix D. Questionnaire English version

Dear student, what should I write on my wall? (Questionnaire on the use of Facebook and Instagram in the educational process)

Dear participant,

Prof. univ. dr. Claudiu Coman (Transilvania University of Brașov) and Conf. Dr. Laurențiu Țîru (West University of Timisoara) invites you to participate in the study about "Using Facebook and Instagram in the educational process". We are interested in knowing your opinion on how these platforms could be used in the educational process, by both teachers and students.

You will be asked to answer the questions contained in the questionnaire on the above mentioned topic. The questionnaire takes about 15 minutes.

You may be vulnerable only if personal data will be associated with the answers provided in the study. We assume full responsibility for the protection of personal data, for ensuring anonymity and confidentiality (no email addresses or other data through which you could be identified are collected).

Each participant has the right to withdraw at any time.

Participation in the study is voluntary.

This study is conducted under the auspices of Transilvania University of Brasov and West University of Timisoara and the results will be published in a scientific journal.

If you have any questions, you can contact us at the e-mail addresses: [claudiu.coman@unitbv.ro](mailto:claudiu.coman@unitbv.ro) or [laurentiu.tiru@e-uvt.ro](mailto:laurentiu.tiru@e-uvt.ro) .

Thank you!

* Required

If you would like to participate in this study, please express your agreement / disagreement with the following aspects of the research:

I declare that I understand the purpose of the research, the procedure, the risks and the fact that the research is voluntary. I understand that I can withdraw at any time without being penalized in any way. *

*Check all applicable options.*

YES

I agree that the data provided in this research will be processed and published by the researcher. *

YES

I declare that I am over 18 years old. *

YES

By clicking on the "Yes" button you declare that you have read the information in this form and that you agree to participate in this study. *

YES

I. In the beginning, you will read a series of statements about how the Facebook platform could be used by teachers in the educational process. Please express your agreement or disagreement with these statements.

Please specify on a scale of 1 to 7, to what extent you consider Facebook to be an appropriate platform for: *

1- TOTAL DISAGREEMENT ... 7-TOTAL AGREEMENT

*Mark one answer for each row.*

|  | 1 | 2 | 3 | 4 | 5 | 6 | 7 |
| --- | --- | --- | --- | --- | --- | --- | --- |
| 1.Teachers to post information/article/video regarding the course/seminar topics |  |  |  |  |  |  |  |
| 2.Teachers to post links regarding the topics of the course/seminar |  |  |  |  |  |  |  |
| 3.Teachers to offer answers to students’ questions about the tasks/projects that they have to carry out |  |  |  |  |  |  |  |
| 4. Teachers to invite field specialists to debate certain topics |  |  |  |  |  |  |  |
| 5. Teachers to propose various debate topics regarding the theme of the course/seminar |  |  |  |  |  |  |  |
| 6. Teachers to share various experiences with respect to their didactic activity |  |  |  |  |  |  |  |
| 7. Teachers to carry out surveys on certain topics related to the course/seminar |  |  |  |  |  |  |  |
| 8. Teachers to implement a teaching model that is focused on students’ needs |  |  |  |  |  |  |  |
| 9. Teachers to post announcement of jobs that might be of interest to students |  |  |  |  |  |  |  |
| 10.Teachers to post announcements about internship opportunities |  |  |  |  |  |  |  |
| 11.Teachers to post announcements about personal development workshops |  |  |  |  |  |  |  |
| 12.Teachers to post announcements about volunteering opportunities |  |  |  |  |  |  |  |
| 13.Teachers to post announcements about various project of interest at the community level |  |  |  |  |  |  |  |
| 14.Teachers to post announcements about various partnerships that the faculty has |  |  |  |  |  |  |  |
| 15.Teachers to post announcements about opportunities to develop the internship required by the faculty |  |  |  |  |  |  |  |
| 16.Teachers to give feedback on certain course/seminar activities carried out by students |  |  |  |  |  |  |  |
| 17. Teachers to give feedback on certain projects before the students have to hand them in |  |  |  |  |  |  |  |
| 18.Teachers to chat with student about the result of their evaluation |  |  |  |  |  |  |  |
| 19. Teachers to share with students information regarding organizational and administrative aspects of the faculty |  |  |  |  |  |  |  |
| 20.Teachers to maintain connection with students |  |  |  |  |  |  |  |
| 21.Teachers to maintain connection with graduates |  |  |  |  |  |  |  |
| 22.Teachers to announce changes regarding the courses/deadlines |  |  |  |  |  |  |  |
| 23.Teachers to communicate with students about certain personal problems that have an impact on their academic performance |  |  |  |  |  |  |  |
| 24.Teachers to manage o series of situation that requires group interaction and consensus |  |  |  |  |  |  |  |
| 25.Teachers to positively influence students through personal example by posting information about the activities they carry out |  |  |  |  |  |  |  |
| 26.Teachers to share scientific information outside of the course/seminar curriculum |  |  |  |  |  |  |  |
| 27.Teachers to post information about the projects they are involved in |  |  |  |  |  |  |  |
| 28.Teachers to post information about their recent publications |  |  |  |  |  |  |  |
| 29.Teachers to post information about conferences of scientific interest |  |  |  |  |  |  |  |
| 30.Teachers to post information about workshops of scientific interest |  |  |  |  |  |  |  |
| 31. Teachers to test research instruments |  |  |  |  |  |  |  |
| 32. Teachers to test research ideas |  |  |  |  |  |  |  |
| 33.Teachers to post information aimed at promoting continuing study programs (master, PhD, etc.) |  |  |  |  |  |  |  |
| 34.Teachers to post information aimed at promoting socio-cultural events that are carried out/will be carried out within their faculty |  |  |  |  |  |  |  |
| 35.Teachers to post information about other extracurricular events that are carried out/will be carried out within their faculty |  |  |  |  |  |  |  |

II. Next, the following statements refer to how the Instagram platform could be used by students in the educational process. Please express your agreement or disagreement with these statements.

Please specify on a scale of 1 to 7, to what extent you consider Instagram to be an appropriate platform for: *

1- TOTAL DISAGREEMENT ... 7-TOTAL AGREEMENT

*Mark one answer for each row.*

|  | 1 | 2 | 3 | 4 | 5 | 6 | 7 |
| --- | --- | --- | --- | --- | --- | --- | --- |
| 1. Students to post information/articles/videos regarding the course/seminar topics |  |  |  |  |  |  |  |
| 2. Students to post links regarding the topics of the course/seminar |  |  |  |  |  |  |  |
| 3. Students to post questions about the tasks/projects that they have to carry out |  |  |  |  |  |  |  |
| 4. Students to post tasks/projects/ essays they have done |  |  |  |  |  |  |  |
| 5. Students to collaborate on carrying out various projects/task/essays for the seminar activity |  |  |  |  |  |  |  |
| 6. Students to propose varied debate topics regarding the theme of the course/seminar. |  |  |  |  |  |  |  |
| 7. Students to share experiences related to the didactic activity |  |  |  |  |  |  |  |
| 8. Students to share various ideas regarding the didactic activity |  |  |  |  |  |  |  |
| 9. Students to carry out surveys on certain topics related to the course/seminar |  |  |  |  |  |  |  |
| 10. Students to post announcements about jobs of interest for other students |  |  |  |  |  |  |  |
| 11. Students to post announcements about internship opportunities |  |  |  |  |  |  |  |
| 12. Students to post announcements about personal development workshops |  |  |  |  |  |  |  |
| 13. Students to post announcements about volunteering opportunities |  |  |  |  |  |  |  |
| 14. Students to post announcements about various projects on interest at the community level |  |  |  |  |  |  |  |
| 15. Students to post feedback about the course/seminar activity |  |  |  |  |  |  |  |
| 16. Students to receive feedback from their peers on their essays/projects |  |  |  |  |  |  |  |
| 17. Students to keep in touch with other students |  |  |  |  |  |  |  |
| 18. Students to post announcements about changes related to the course, deadlines, etc. |  |  |  |  |  |  |  |
| 19. Students to manage a series of situations that require interaction and group consensus |  |  |  |  |  |  |  |
| 20. Students to share extracurricular information |  |  |  |  |  |  |  |
| 21. Students to post information about a series of students specific scientific conferences/ sessions |  |  |  |  |  |  |  |
| 22. Students to post information about a series of students specific scientific conferences/ sessions |  |  |  |  |  |  |  |
| 23. Students to post information about workshops of scientific interest |  |  |  |  |  |  |  |
| 24. Students to post information aimed at promoting continuing study programs (master, PhD) |  |  |  |  |  |  |  |
| 25. Students to post information aimed at promoting socio-cultural events that are carried out/will be carried out within their faculty |  |  |  |  |  |  |  |
| 26. Students to post information about other extracuricular events that are carried out/will be carried out within their faculty |  |  |  |  |  |  |  |

III. Next, the following statements refer to how the Instagram platform could be used by teachers in the educational process. Please express your agreement or disagreement with these statements.

Please specify on a scale of 1 to 7, to what extent you consider Instagram to be an appropriate platform for: *

1- TOTAL DISAGREEMENT ... 7-TOTAL AGREEMENT

*Mark one answer for each row.*

|  | 1 | 2 | 3 | 4 | 5 | 6 | 7 |
| --- | --- | --- | --- | --- | --- | --- | --- |
| 1. Teachers to post photos/various types of presentation/videos related to the topic of the course/seminar |  |  |  |  |  |  |  |
| 2. Teachers to invite field specialists to post photos/various types of presentations related to the course/seminar topic |  |  |  |  |  |  |  |
| 3. Teachers to propose debate topics starting from photos/ various types of presentations/videos related to the course/seminar topic |  |  |  |  |  |  |  |
| 4. Teachers to share personal experiences related to their didactic activity |  |  |  |  |  |  |  |
| 5. Teachers to propose ideas regarding the didactic activity |  |  |  |  |  |  |  |
| 6. Teachers to post photos, types of presentation, videos developed during the course/seminar |  |  |  |  |  |  |  |
| 7. Teachers to post photos/ various types of presentations/videos developed during certain extracurricular activities |  |  |  |  |  |  |  |
| 8. Teachers to post photos/various types of presentations/videos developed together with other students during certain extracurricular activities |  |  |  |  |  |  |  |
| 9. Teachers to post announcements about jobs of interest for students |  |  |  |  |  |  |  |
| 10. Teachers to post announcements about internship opportunities |  |  |  |  |  |  |  |
| 11. Teachers to post announcements about personal development workshops |  |  |  |  |  |  |  |
| 12. Teachers to post announcements about volunteering opportunities |  |  |  |  |  |  |  |
| 13. Teachers to post announcements about various projects of interest at the community level |  |  |  |  |  |  |  |
| 14. Teachers to post announcements about various partnerships of the faculty |  |  |  |  |  |  |  |
| 15. Teachers to post announcement about opportunities to develop the internship required by the faculty |  |  |  |  |  |  |  |
| 16. Teachers to share with students information regarding organizational or administrative aspects of the faculty |  |  |  |  |  |  |  |
| 17. Teachers to maintain connection with students |  |  |  |  |  |  |  |
| 18. Teachers to maintain connesction with graduates |  |  |  |  |  |  |  |
| 19. Teachers to announce changes regarding the course, deadlines, etc |  |  |  |  |  |  |  |
| 20. Teachers to positively influence students through personal example by posting information about the activities they carry out |  |  |  |  |  |  |  |
| 21. Teachers to share scientific information outside of the course/seminar curriculum |  |  |  |  |  |  |  |
| 22. Teachers to post information about the project they are involved in |  |  |  |  |  |  |  |
| 23. Teachers to post information about their recent publications |  |  |  |  |  |  |  |
| 24. Teachers to post information about conferences of scientific interest |  |  |  |  |  |  |  |
| 25. Teachers to post information about workshops of interest in the study field |  |  |  |  |  |  |  |
| 26. Teachers to post information aimed at promoting continuing study programs (master, PhD) |  |  |  |  |  |  |  |
| 27. Teachers to post information aimed at promoting socio-cultural events that are carried out/will be carried out within their faculty |  |  |  |  |  |  |  |
| 28. Teachers to post information about other extracurricular events that are carried out/will be carried out within their faculty |  |  |  |  |  |  |  |

IV. Next, you will read a series of statements about how the Instagram platform could be used by students in the educational process. Please express your agreement or disagreement with these statements.

Please specify on a scale of 1 to 7, to what extent you consider Instagram to be an appropriate platform for: *

1- TOTAL DISAGREEMENT ... 7-TOTAL AGREEMENT

*Mark one answer for each row*

|  | 1 | 2 | 3 | 4 | 5 | 6 | 7 |
| --- | --- | --- | --- | --- | --- | --- | --- |
| 1. Students to post photos/various types of presentation/videos related to the topic of the course/seminar |  |  |  |  |  |  |  |
| 2. Students to collaborate with their peers on carrying out various projects/task/essays for the seminar activity |  |  |  |  |  |  |  |
| 3. Students to propose varied debate topics regarding the theme of the course/seminar. |  |  |  |  |  |  |  |
| 4. Students to share various experiences with respect to their didactic activity |  |  |  |  |  |  |  |
| 5. Students to share various ideas with respect to their didactic activity |  |  |  |  |  |  |  |
| 6. Students to post announcements about jobs of interest for other students |  |  |  |  |  |  |  |
| 7. Students to post announcements about internship opportunities |  |  |  |  |  |  |  |
| 8. Students to post announcements about personal development workshops |  |  |  |  |  |  |  |
| 9. Students to post announcements about volunteering opportunities |  |  |  |  |  |  |  |
| 10. Students to post announcements about various projects on interest at the community level |  |  |  |  |  |  |  |
| 11. Students to post feedback about the course/seminar activity |  |  |  |  |  |  |  |
| 12. Students to receive feedback from their peers on their essays/projects |  |  |  |  |  |  |  |
| 13. Students to keep in touch with other students |  |  |  |  |  |  |  |
| 14. Students to post announcements about changes related to the course, deadlines, etc. |  |  |  |  |  |  |  |
| 15. Students to manage situations that requires interaction and group consensus |  |  |  |  |  |  |  |
| 16. Students to share extracurricular information |  |  |  |  |  |  |  |
| 17. Students to post information about the projects they are involved in |  |  |  |  |  |  |  |
| 18. Students to post information about a series of students specific scientific conferences/ sessions |  |  |  |  |  |  |  |
| 19. Students to post information about workshops of scientific interest |  |  |  |  |  |  |  |
| 20. Students to post information aimed at promoting continuing study programs (master, PhD) |  |  |  |  |  |  |  |
| 21. Students to post information aimed at promoting socio-cultural events that are carried out/will be carried out within their faculty |  |  |  |  |  |  |  |
| 22. Students to post information about other extracurricular events that are carried out/will be carried out within their faculty |  |  |  |  |  |  |  |

Finally, please answer some socio-demographic questions

Do you have Facebook?*

YES

NO

How often do you access this account? *

daily

3-4 times a week

1-2 times a week

several times a month

rarely

It is not the case/I do not have an account

Do you have an Instagram account?*

YES

NO

How often do you access this account? *

daily

3-4 times a week

1-2 times a week

several times a month

rarely

It is not the case/I do not have an account

Your age: *

____________________

Your gender: *

Masculin

Feminin

Geographical location: *

Urban

Rural

City where you study: *

______________________________

University where you study: *

___________________________________________

Currently you are: *

A Bachelor student

A Master student

A PhD students

What is the field in which you are taking courses?*

Communication science

Psychology and educational sciences

Sociology and social work

Administrative sciences

Political Sciences

Engineering

Humanities and arts

Economics

Mathematic and informatics

Biological and medical sciences

Sports and physical education

Legal Sciences

Military sciences, information and public order

Natural Sciences

Other: _______________________________

Thank you for your willingness to respond!
