# Supplementary material for: Dear student, what should I write on my wall? A case study on academic uses of Facebook and Instagram during the pandemic
Source: PLoS One. 2021 Sep 23;16(9):e0257729. doi: 10.1371/journal.pone.0257729 (PMC8459956; doi:10.1371/journal.pone.0257729)
Supplement: S5 Appendix — (DOCX) [file pone.0257729.s005.docx]

Dear student, what should I write on my wall? (Chestionar despre utilizarea platormelor Facebook și Instagram în procesul educațional)

Stimate participant,

Prof. univ. dr. Claudiu Coman (Universitatea Transilvania din Brașov) și Conf. univ. dr. Laurențiu Țîru (Universitatea de Vest) vă invită să participați la studiul privind "Utilizarea platformelor Facebook și Instagram în procesul educațional". Suntem interesați de cunoașterea opiniilor voastre privind modalitățile în care aceste platforme ar putea fi utilizate în procesul educațional, atât de către profesori cât și de studenți.

Veți fi solicitat/ă să răspundeți în scris la întrebările cuprinse în chestionarul pe tema mai sus menționată, pe durata a 15 minute.

Puteți fi vulnerabil/ă doar dacă datele personale vor fi asociate cu răspunsurile furnizate în cadrul studiului. Ne asumăm întreaga responsabilitate pentru protejarea datelor personale, pentru asigurarea anonimatului și a confidențialității (nu se colectează adrese de e-mail sau alte date prin care ați putea fi identificați).

Fiecare participant are dreptul să se retragă în orice moment. Participarea la studiu este voluntară.

Acest studiu este realizat sub egida Universității Transilvania din Braşov și a Universității de Vest din Timișoara şi rezultatele vor fi publicate într-o revistă științifică.

Dacă aveți întrebări, puteți să ne contactați la adresele de e-mail [claudiu.coman@unitbv.ro](mailto:claudiu.coman@unitbv.ro) sau [laurentiu.tiru@e-uvt.ro](mailto:laurentiu.tiru@e-uvt.ro)

Vă mulțumim!

*Obligatoriu

Dacă doriți să participați la acest studiu, vă rugăm frumos să vă exprimați acordul/dezacordul cu următoarele aspecte legate de cercetare:

Declar că am înțeles care este scopul cercetării, procedura, riscurile și faptul că cercetarea este voluntară. Am înțeles că pot să mă retrag oricând fără a fi penalizat în niciun fel. *

*Bifați toate variantele aplicabile.*

DA

Sunt de acord ca datele furnizate în această cercetare să fie prelucrate și publicate de către cercetător. *

DA

Declar că am cel puțin 18 ani. *

DA

Prin apăsarea butonului ”Da” declarați că ați luat la cunoștință de informațiile din acest formular și că sunteți de acord să participați la acest studiu. *

DA

I. Pentru început, veți citi o serie de afirmații referitoare la modul în care platforma Facebook ar putea fi utilizată de către profesori în procesul educațional. Vă rugăm să vă exprimați acordul sau dezacordul cu aceste afirmații.

Vă rugăm să precizați pe o scală de la 1 la 7, în ce măsură considerați că Facebook este un loc potrivit pentru ca: *

1- TOTAL DEZACORD... 7-TOTAL ACORD

*Marcați un singur oval pentru fiecare rând.*

|  | 1 | 2 | 3 | 4 | 5 | 6 | 7 |
| --- | --- | --- | --- | --- | --- | --- | --- |
| 1.Profesorii să posteze informații/articole/video-uri privind tematica cursului/seminarului. |  |  |  |  |  |  |  |
| 2.Profesorii să posteze link-uri referitoare la tematica cursului/seminarului. |  |  |  |  |  |  |  |
| 3.Profesorii să ofere răspunsuri la întrebările studenților privind temele/proiectele pe care le au de realizat. |  |  |  |  |  |  |  |
| 4.Profesorii să invite specialiști în domeniu pentru diverse dezbateri. |  |  |  |  |  |  |  |
| 5.Profesorii să propună diverse teme de dezbatere privind tematica cursului/seminarului. |  |  |  |  |  |  |  |
| 6.Profesorii să împărtășească diverse experiențe legate de activitatea didactică. |  |  |  |  |  |  |  |
| 7.Profesorii să realizeze sondaje pe anumite tematici legate de curs/seminar. |  |  |  |  |  |  |  |
| 8.Profesorii să poată să implementeze un model de predare centrat pe nevoile studenților. |  |  |  |  |  |  |  |
| 9.Profesorii să posteze anunțuri despre job-uri de interes pentru studenți. |  |  |  |  |  |  |  |
| 10.Profesorii să posteze anunțuri despre posibilități de internshipuri |  |  |  |  |  |  |  |
| 11.Profesorii să posteze anunțuri despre workshop-uri de dezvoltare personală. |  |  |  |  |  |  |  |
| 12.Profesorii să posteze anunțuri despre oportunități de a face voluntariat. |  |  |  |  |  |  |  |
| 13.Profesorii să posteze anunțuri despre diverse proiecte de interes la nivel comunitar. |  |  |  |  |  |  |  |
| 14.Profesorii să posteze anunțuri despre diverse parteneriate pe care le are facultatea. |  |  |  |  |  |  |  |
| 15.Profesorii să posteze anunțuri despre posibilități de a desfășura practica. |  |  |  |  |  |  |  |
| 16.Profesorii să dea feedback despre anumite activități ale studenților de la seminar/curs. |  |  |  |  |  |  |  |
| 17.Profesorii să dea feedback despre anumite teme/proiecte înainte de a fi predate. |  |  |  |  |  |  |  |
| 18.Profesorii să aibă diverse discuții legate de rezultatele evaluării. |  |  |  |  |  |  |  |
| 19.Profesorii să distribuie către studenți informații ce vizează aspecte organizatorice și administrative ale facultății |  |  |  |  |  |  |  |
| 20.Profesorii să mențină legătura cu studenții. |  |  |  |  |  |  |  |
| 21.Profesorii să mențină legătura cu absolvenții. |  |  |  |  |  |  |  |
| 22.Profesorii să anunțe modificări privind cursurile, termenele limite etc. |  |  |  |  |  |  |  |
| 23.Profesorii să comunice cu studenții privind unele probleme personale cu impact asupra performanței lor școlare |  |  |  |  |  |  |  |
| 24.Profesorii să gestioneze o serie de situații ce necesită interacțiunea și consensul grupului. |  |  |  |  |  |  |  |
| 25.Profesorii să influențeze pozitiv studenții prin postările și activitatea lor de pe Facebook prin exemplul personal |  |  |  |  |  |  |  |
| 26.Profesorii să împărtășească informații științifice, dar care nu fac parte din curricula cursului/seminarului. |  |  |  |  |  |  |  |
| 27.Profesorii să posteze informații despre proiectele în care sunt implicați. |  |  |  |  |  |  |  |
| 28.Profesorii să posteze informații despre publicațiile lor recente. |  |  |  |  |  |  |  |
| 29.Profesorii să posteze informații despre conferințe de interes științific.. |  |  |  |  |  |  |  |
| 30.Profesorii să posteze informații despre workshopuri de interes științific. |  |  |  |  |  |  |  |
| 31.Profesorii să testeze instrumente de cercetare. |  |  |  |  |  |  |  |
| 32.Profesorii să testeze idei de cercetare. |  |  |  |  |  |  |  |
| 33.Profesorii să posteze informații care vizează promovarea programelor de continuarea studiilor (master, doctorat etc |  |  |  |  |  |  |  |
| 34.Profesorii să posteze informații care vizează promovarea unor evenimente socio-culturale care se desfășoară/ se vor desfășura în facultate. |  |  |  |  |  |  |  |
| 35.Profesorii să posteze informații despre alte evenimente extra-currriculare care se desfășoară/ se vor desfășura în facultate. |  |  |  |  |  |  |  |

II. În continuare, următoarele afirmații fac referire la modul în care platforma Facebook ar putea fi utilizată de către studenți în procesul educational. Vă rugăm să vă exprimați acordul sau dezacordul cu aceste afirmații.

Vă rugăm să precizați pe o scală de la 1 la 7, în ce măsură considerați că Facebook este un loc potrivit pentru ca: *

1- TOTAL DEZACORD... 7-TOTAL ACORD

*Marcați un singur oval pentru fiecare rând.*

|  | 1 | 2 | 3 | 4 | 5 | 6 | 7 |
| --- | --- | --- | --- | --- | --- | --- | --- |
| 1.Studenții să posteze informații/articole/video-uri privind tematica cursului/seminarului. |  |  |  |  |  |  |  |
| 2.Studenții să posteze link-uri referitoare la tematica cursului/seminarului. |  |  |  |  |  |  |  |
| 3.Studenții să posteze întrebări privind temele/proiectele pe care le au de realizat. |  |  |  |  |  |  |  |
| 4.Studenții să posteze teme/proiecte/eseuri. |  |  |  |  |  |  |  |
| 5.Studenții să colaboreze pentru realizarea proiectelor/eseurilor/temelor de seminar |  |  |  |  |  |  |  |
| 6.Studenții să propună diverse teme de dezbatere privind tematica cursului/seminarului. |  |  |  |  |  |  |  |
| 7.Studenții să împărtășească diverse experiențe legate de activitatea didactică. |  |  |  |  |  |  |  |
| 8.Studenții să împărtășească diverse idei legate de activitatea didactică. |  |  |  |  |  |  |  |
| 9.Studenții să realizeze sondaje pe anumite tematici legate de curs/seminar. |  |  |  |  |  |  |  |
| 10.Studenții să posteze anunțuri despre job-uri de interes pentru studenți. |  |  |  |  |  |  |  |
| 11.Studenții să posteze anunțuri despre posibilități de internshipuri. |  |  |  |  |  |  |  |
| 12.Studenții să posteze anunțuri despre workshop-uri de dezvoltare personală. |  |  |  |  |  |  |  |
| 13.Studenții să posteze anunțuri despre posibilități de a face voluntariat. |  |  |  |  |  |  |  |
| 14.Studenții să posteze anunțuri despre diverse proiecte de interes la nivel comunitar. |  |  |  |  |  |  |  |
| 15.Studenții să posteze feedback-uri legate de activitatea de la curs/seminar. |  |  |  |  |  |  |  |
| 16.Studenții să primească feedback la temele/eseurile/proiectele lor de la ceilalți studenți. |  |  |  |  |  |  |  |
| 17.Studenții să mențină legătura cu studenții. |  |  |  |  |  |  |  |
| 18.Studenții să posteze anunțuri privind modificări ce țin de cursuri, termenele limite |  |  |  |  |  |  |  |
| 19.Studenții să gestioneze o serie de situații ce necesită interacțiunea și consensul grupului. |  |  |  |  |  |  |  |
| 20.Studenții să partajeze informații extracuriculare |  |  |  |  |  |  |  |
| 21.Studenții să posteze informații despre proiectele în care sunt implicați. |  |  |  |  |  |  |  |
| 22.Studenții să posteze informații despre o serie de conferințe/sesiuni științifice specifice studenților |  |  |  |  |  |  |  |
| 23.Studenții să posteze informații despre workshopuri de interes științific. |  |  |  |  |  |  |  |
| 24.Studenții să posteze informații care vizează promovarea programelor de continuarea studiilor (master, doctorat) |  |  |  |  |  |  |  |
| 25.Studenții să posteze informații care vizează promovarea unor evenimente socio-culturale care se desfășoară/ se vor desfășura în facultate. |  |  |  |  |  |  |  |
| 26.Studenții să posteze informații despre alte evenimente extra-currriculare care se desfășoară/ se vor desfășura în facultate. |  |  |  |  |  |  |  |

III. În continuare, următoarele afirmații fac referire la modul în care platforma Instagram ar putea fi utilizată de către profesori în procesul educațional

Vă rugăm să precizați pe o scală de la 1 la 7, în ce măsură considerați că Instagram este un loc potrivit pentru ca: *

1- TOTAL DEZACORD... 7-TOTAL ACORD

*Marcați un singur oval pentru fiecare rând.*

|  | 1 | 2 | 3 | 4 | 5 | 6 | 7 |
| --- | --- | --- | --- | --- | --- | --- | --- |
| 1.Profesorii să posteze fotografii/diverse tipuri de prezentări /video-uri privind tematica cursului/seminarului |  |  |  |  |  |  |  |
| 2.Profesorii să invite specialiști în domeniu pentru a posta fotografii/diverse tipuri de prezentări /video-uri referitoare la tematica cursului. |  |  |  |  |  |  |  |
| 3.Profesorii să propună teme de dezbatere pornind de la fotografii/ tipuri de prezentări /video-uri legate de tematica cursului/seminarului. |  |  |  |  |  |  |  |
| 4.Profesorii să împărtășească experiențe personale legate de activitatea didactică. |  |  |  |  |  |  |  |
| 5.Profesorii să propună idei legate de activitatea didactică. |  |  |  |  |  |  |  |
| 6.Profesorii să posteze fotografii, tipuri de prezentări sau video-uri din timpul unor activități de curs/seminar |  |  |  |  |  |  |  |
| 7.Profesorii să posteze fotografii, diverse tipuri de prezentări sau video-uri din timpul realizării unor proiecte curriculare. |  |  |  |  |  |  |  |
| 8.Profesorii să posteze fotografii, tipuri de prezentări sau video-uri din timpul realizării unor proiecte extracurriculare realizate cu studenții. |  |  |  |  |  |  |  |
| 9.Profesorii să posteze anunțuri despre job-uri de interes pentru studenți. |  |  |  |  |  |  |  |
| 10.Profesorii să posteze anunțuri despre oportunități de internshipuri. |  |  |  |  |  |  |  |
| 11.Profesorii să posteze anunțuri despre workshop-uri de dezvoltare personală. |  |  |  |  |  |  |  |
| 12.Profesorii să posteze anunțuri despre oportunități de a face voluntariat. |  |  |  |  |  |  |  |
| 13.Profesorii să posteze anunțuri despre diverse proiecte de interes la nivel comunitar |  |  |  |  |  |  |  |
| 14.Profesorii să posteze anunțuri despre diverse parteneriate pe care le are facultatea. |  |  |  |  |  |  |  |
| 15.Profesorii să posteze anunțuri despre posibiltăți de desfășura practica. |  |  |  |  |  |  |  |
| 16.Profesorii să distribuie studenților informațiii ce vizează aspecte organizatorice și administrative ale facultății. |  |  |  |  |  |  |  |
| 17.Profesorii să mențină legătura cu studenții. |  |  |  |  |  |  |  |
| 18.Profesorii să mențină legătura cu absolvenții. |  |  |  |  |  |  |  |
| 19.Profesorii să anunțe modificări privind cursurile, termenele limită |  |  |  |  |  |  |  |
| 20.Profesorii să influențeze pozitiv studenții prin postările și activitatea lor de pe instagram prin exemplul personal |  |  |  |  |  |  |  |
| 21.Profesorii să împărtășească informații științifice, dar care nu fac parte din curricula cursului/seminarului. |  |  |  |  |  |  |  |
| 22.Profesorii să posteze informații despre proiectele în care sunt implicați. |  |  |  |  |  |  |  |
| 23.Profesorii să posteze informații despre publicațiile lor recente. |  |  |  |  |  |  |  |
| 24.Profesorii să posteze informații despre conferințe de interes academic. |  |  |  |  |  |  |  |
| 25.Profesorii să posteze informații despre workshopuri de interes pentru domeniul studiat. |  |  |  |  |  |  |  |
| 26.Profesorii să posteze informații care vizează promovarea programelor de continuarea studiilor (master, doctorat) |  |  |  |  |  |  |  |
| 27.Profesorii să posteze informații care vizează promovarea unor evenimente socio-culturale care se desfășoară/ se vor desfășura în facultate. |  |  |  |  |  |  |  |
| 28.Profesorii să posteze informații despre alte evenimente extra-currriculare care se desfășoară/ se vor desfășura în facultate. |  |  |  |  |  |  |  |

IV. În continuare, veți citi o serie de afirmații referitoare la modul în care platforma Instagram ar putea fi utilizată de către studenți în procesul educațional

Vă rugăm să precizați pe o scală de la 1 la 7, în ce măsură considerați că Instagram este un loc potrivit pentru ca: *

1- TOTAL DEZACORD... 7-TOTAL ACORD

*Marcați un singur oval pentru fiecare rând.*

|  | 1 | 2 | 3 | 4 | 5 | 6 | 7 |
| --- | --- | --- | --- | --- | --- | --- | --- |
| 1.Studenții să posteze fotografii/diverse prezentări/video-uri privind tematica cursului/seminarului. |  |  |  |  |  |  |  |
| 2.Studenții să discute cu colegii despre realizarea unor proiecte/eseuri/teme pentru seminar. |  |  |  |  |  |  |  |
| 3.Studenții să propună diverse teme de dezbatere privind tematica cursului/seminarului. |  |  |  |  |  |  |  |
| 4.Studenții să împărtășească diverse experiențe legate de activitatea didactică. |  |  |  |  |  |  |  |
| 5.Studenții să împărtășească diverse idei legate de activitatea didactică. |  |  |  |  |  |  |  |
| 6.Studenții să posteze anunțuri despre job-uri de interes pentru studenți. |  |  |  |  |  |  |  |
| 7.Studenții să posteze anunțuri despre oportunități de internshipuri. |  |  |  |  |  |  |  |
| 8.Studenții să posteze anunțuri despre workshop-uri de dezvoltare personală. |  |  |  |  |  |  |  |
| 9.Studenții să posteze anunțuri despre posibilități de a face voluntariat. |  |  |  |  |  |  |  |
| 10.Studenții să posteze anunțuri despre diverse proiecte de interes la nivel comunitar. |  |  |  |  |  |  |  |
| 11.Studenții să posteze feedback-uri legate de activitatea de la curs/seminar. |  |  |  |  |  |  |  |
| 12.Studenții să primească feedback la temele/eseurile/proiectele lor de la ceilalți studenți. |  |  |  |  |  |  |  |
| 13.Studenții să mențină legătura cu studenții |  |  |  |  |  |  |  |
| 14.Studenții să posteze anunțuri privind modificări ce țin de cursuri, termenele limite etc. |  |  |  |  |  |  |  |
| 15.Studenții să gestioneze o serie de situații ce necesită interacțiunea și consensul grupului. |  |  |  |  |  |  |  |
| 16.Studenții să distribuie informații extracuriculare |  |  |  |  |  |  |  |
| 17.Studenții să posteze informații despre proiectele în care sunt implicați. |  |  |  |  |  |  |  |
| 18.Studenții să posteze informații despre o serie de conferințe/sesiuni științifice destinate studenților |  |  |  |  |  |  |  |
| 19.Studenții să posteze informații despre workshopuri de interes științific. |  |  |  |  |  |  |  |
| 20.Studenții să posteze informații care vizează promovarea programelor de continuarea studiilor (master, doctorat) |  |  |  |  |  |  |  |
| 21.Studenții să posteze informații care vizează promovarea unor evenimente socio-culturale care se desfășoară/ se vor desfășura în facultate. |  |  |  |  |  |  |  |
| 22.Studenții să posteze informații despre alte evenimente extra-currriculare care se desfășoară/ se vor desfășura în facultate. |  |  |  |  |  |  |  |

În final, vă rugăm să ne mai răspundeți la câteva întrebări socio-demografice

Aveți cont de Facebook? *

DA

NU

Cât de des accesați acest cont? *

zilnic

de 3-4 ori pe săptămână

de 1-2 ori pe săptămână

de câteva ori pe lună

mai rar

Nu e cazul/ nu am cont

Aveți cont de Instagram? *

DA

NU

Cât de des accesați acest cont? *

zilnic

de 3-4 ori pe săptămână

de 1-2 ori pe săptămână

de câteva ori pe lună

mai rar

Nu e cazul/ nu am cont

Vârsta dumneavoastră: *

____________________

Genul dumneavoastră *

Masculin

Feminin

Mediul de proveniență *

Urban

Rural

Orașul în care vă desfășurați studiile: *

______________________________

Universitatea în cadrul căreia vă desfășurați studiile *

___________________________________________

Sunteți student (ă) la ciclul de: *

Licență

Masterat

Doctorat

Sunteți student(ă) la o facultate din domeniul *

Științe ale comunicării

Psihologie și științe ale educației

Sociologie și Asistență socială

Științe administrative

Științe politice

Științe inginerești

Științe umaniste și arte

Științe economice

Matematică/ informatică

Științe biologice și medicale

Știința sportului și educației fizice

Științe juridice

Științe militare, informații și ordine publică

Științe ale naturii

Altele_______________________________

Vă mulțumim pentru disponibilitatea de a răspunde!
