# Supplementary material for: Dear student, what should I write on my wall? A case study on academic uses of Facebook and Instagram during the pandemic
Source: PLoS One. 2021 Sep 23;16(9):e0257729. doi: 10.1371/journal.pone.0257729 (PMC8459956; doi:10.1371/journal.pone.0257729)
Supplement: Attachment — Submitted filename: PONE-D-21-10280.docx [file pone.0257729.s006.docx]

Reviewer Comments

Line 61 Although E-learning platforms or social networks were used by higher education institutions in the educational process prior to the COVID 19 pandemic, due to the pandemic

Change to

Line 61 Although E-learning platforms or social networks were used by higher education institutions in the educational process prior to the COVID-19 pandemic, due to the pandemic,

Line 66 Recent studies that focused on identifying the perception of Romanian students about online learning during the pandemic [8,9], revealed less positive attitudes towards this type of learning, students considering that the online educational process has less value than the traditional one, them preferring to use E-learning platforms as a complementary method to traditional, face to face learning

Change to

Line 66 Recent studies that focused on identifying the perception of Romanian students about online learning during the pandemic [8,9], revealed less positive attitudes towards this type of learning, students considering that the online educational process has less value than the traditional one, preferring instead to use E-learning platforms as a complementary method to traditional, face-to-face learning.

Line 73 Moreover, a study conducted on Romanian students [8], also revealed that even though students mostly used E-learning platforms, in certain circumstances they would have preferred to use other online platforms in order to compensate for the disadvantages of the E-learning platform, such as technical problems, lack of proper functions that allow collaborative learning.

Change to

Line 73 Moreover, a study conducted on Romanian students [8], also revealed that even though students frequently E-learning platforms, in certain circumstances they would have preferred to use other online platforms in order to compensate for the disadvantages of the E-learning platform, such as technical problems, lack of proper functions that allow collaborative learning.

Line 85 Facebook and Instagram were the focus of our research due to the fact that they are two of the most popular and used social networks worldwide

Change to

Line 85 Facebook and Instagram were the focus of our research because they are two of the most popular and used social networks worldwide.

Line 89 these tools, what they would like their teachers to post on these platforms and what types of activities would they prefer to carry out on them

Change to

Line 89 these tools, what they would like their teachers to post on these platforms, and what types of activities would they prefer to carry out on them.

Line 145 Students no longer use social networks only for connecting and interacting with friends or relatives [30] and these sites are nowadays also adopted by teachers because teaching and learning without technology often seems uninteresting.

Change to

Line 145 Students no longer use social networks only for connecting and interacting with friends or relatives [30], and these sites are nowadays also adopted by teachers because teaching and learning without technology often seems uninteresting.

Line 150 Even more, with the development of online courses, it is important to understand the way students think and how do they approach learning in an environment where learning is very different from how it can usually be done in traditional courses

Change to

Line 150 Even more, with the development of online courses, it is important to understand the methods students use to learn and the manner in which they think in an environment where learning is very different from how it can usually be done in traditional courses.

Line 157 When teaching online, teachers must provide clear instructions, the content of the course must be delivered in a smooth, simple manner [35], and it is not recommended to attempt to create online the traditional course, but to adapt it and focus on the benefits offered by the environment

Change to

Line 157 When teaching online, teachers must provide clear instructions, the content of the course must be delivered in a smooth, simple manner [35], and , and adapt it to focus on the benefits offered by the environment.

Line 162 Besides being used for dissemination of information [39], social networks encourage collaboration, allowing students to share, debate and examine their ideas together with their peers and teachers and can improve students engagement, providing the opportunity to learn in an informal environment, supporting intimidated or less involved students to express their opinion in a more pleasant way.

Change to

Line 162 Besides being used for dissemination of information [39], social networks encourage collaboration, allowing students to share, debate and examine their ideas together with their peers and teachers, which can improve students engagement; providing the opportunity to learn in an informal environment; and supporting intimidated or less involved students to express their opinion in a more pleasant way.

Line 167 Social networks, help teachers and students maintain continuous contact while setting up new and spaces for learning, [40] they make learning interactive [41] and through them students can access, read the content taught at any date and time.

Change to

Line 167 Social networks, help teachers and students maintain continuous contact while setting up new and spaces for learning; [40] they make learning interactive [41], and through them students can access and read the content taught at any date and time.

Line 171 Studies [42,43] on the use of social networks and their impact on student’s grades showed that they negatively affect student’s academic performance, those using platforms such as Facebook, registering lower grade-point averages, while other studies [44,45], found no connection or significant relationship between the amount of time spent on social networks and grades, thus providing contrasting results on this matter.

Change to

Line 171 Studies [42,43] on the use of social networks and their impact on student’s grades showed that they negatively affect student’s academic performance. Those using platforms such as Facebook, registering lower grade-point averages, while other studies [44,45], found no connection or significant relationship between the amount of time spent on social networks and grades, thus providing contrasting results on this matter.

Line 178 It is a fact that Facebook is the most popular and largest social network nowadays, reaching a wide number of people that use it for multiple purposes.

Change to

Line 178 It is a fact that Facebook is the most popular and largest social network nowadays, reaching a wide number of people who use it for multiple purposes.

Line 180 According to a study on the global internet use, in January 2020 there were 3,8 billion social media users and Facebook, with 2,44 billion monthly users, remains the social network most used worldwide.

Change to

Line 180 According to a study on the global internet use, in January 2020 there were 3,8 billion social media users and Facebook, with 2,44 billion monthly users. It remains the social network most used worldwide.

Line 182 Facebook was originally created within Harvard University’s grounds,[46] being restricted only to Harvard students, allowing them to create personal profiles, interact, socialize and contact other students

Change to

Line 182 Facebook was originally created within Harvard University’s grounds,[46] restricted only to Harvard students, and allowing them to create personal profiles, interact, socialize, and contact other students

Line 186 From a student’s perspective, teachers could use Facebook for creating groups where presentations related to the lectures could be posted, as well as information about different changes with respect to the development of the courses

Change to

Line 186 From a student’s perspective, teachers could use Facebook for creating groups where presentations related to the lectures could be posted, as well as provide information about different changes with respect to the development of the courses.

Line 190 between them and the university, thus students being more willing to study and complete their degree at their current university

Change to

Line 190 between them and the university, thus students are more willing to study and complete their degree at their current university.

Line 201 much more proper medium for social learning than the real one, they preferring to receive tasks on this platform and stating that they favor discovering, searching and accomplishing school tasks through this platform, for it makes them feel more comfortable.

Change to

Line 201 much more proper medium for social learning than the real one; they preferring to receive tasks on this platform and, because they prefer this medium for discovering information, accomplishing school tasks through this platform.

Line 206 accommodate to university life

Change to

Line 206 acclimate to university life.

Line 207 Even more, a large amount of students access Facebook in order to find out more information about people they had recently met, to stay in touch with old and present friends [59],

Change to

Line 207 Even more, a large number of students access Facebook in order to find out more information about people they had recently met and to stay in touch with old and present friends [59]; additional studies [60] show that students would rather interact with friends on public wall spaces, with this type of communication being twice as frequent as exchanging information through private messages, over personal or face-to-face interaction.

Line 211 In addition, it is taught that Facebook is the social network best suited for students [61], and this affirmation is sustained by a study which included students using a Facebook group for academic purposes that uncovered the fact that all students confirmed an improved interaction with their teachers, concluding that the use of social networks could refine their learning experience [62].

Change to

Line 211 In addition, it is thought that Facebook is the social network best suited for students [61], This statement is sustained by a study which included students using a Facebook group for academic purposes confirmed an improved student interaction with their teachers, and, thus, concluding that the use of social networks could refine their learning experience [62].

Line 219 Recognizing the educational potential of Facebook, teachers must find ways to efficiently use it and to understand how to interact with students so as to create positive attitudes towards this type of learning

Change to

Line 219 Recognizing the educational potential of Facebook, teachers must find ways to efficiently use it and understand how to interact with students, so they create positive attitudes towards this type of learning.

Line 226 Instagram is a photo-video sharing platform launched in 2010, currently owned by Facebook, that allows users to post visual content, 24 hours stories, and privately communicate with their friends, the platform registering in 2020 over 1 billion monthly active users.

Change to

Line 226 Instagram is a photo-video sharing platform launched in 2010, currently owned by Facebook, that allows users to post visual content, 24 hours stories, and privately communicate with their friends. The platform registering in 2020 over 1 billion monthly active users.

Line 230 Instagram is especially designed for mobile use and it’s a platform more orientated to mobile experience, one of the reasons for its rapid ascension being the development of smartphones [68], and the excessively use of them.

Change to

Line 230 Instagram is especially designed for mobile use; it is a platform more orientated to a mobile experience. One of the reasons for its rapid ascension is the development and widespread use of smartphones.

Line 232 However, Instagram is about more than just photo sharing, the platform offering the possibility to create communities in many domains, with users being able to broaden their contact list, exchange information, share knowledge through public or private messages, live videos

Change to

Line 232 However, Instagram is about more than just photo sharing. The platform offers the possibility to create communities in many domains, and the ability for users to broaden their contact list, exchange information, share knowledge through public or private messages, live videos.

Line 235 Even more, through its emphasis on visual content, and through the actions of the users: taking the photo, editing it, writing a description, Instagram can help improve student’s spatial or linguistic intelligence

Change to

Line 235 Additionally, through its emphasis on visual content, and through the actions of the users (taking the photo, editing a photo, and writing a description) Instagram can help improve student’s spatial or linguistic intelligence.

Line 237 While students today are considered digital natives who heavily use social networks, [71], professors should integrate such networks as teaching tools, in order to enhance their learning experience, giving them the chance to share ideas and opinions through a medium that they consider pleasant and interesting [72].

Changes

Line 237 This should be added to the previous paragraph. Also, **change to**: While students today are considered digital natives who heavily use social networks,[71], professors should integrate such networks as teaching tools in order to enhance their learning experience and give students the chance to share ideas and opinions through a medium that they consider pleasant and interesting [72].

Line 244 A previous study on the efficiency of Instagram in the field of education indicated that, including this platform in the teaching process helped improve students writing, them being more creative, developing the story in a more attractive way by approaching diverse topics [74].

Change to

Line 244 A previous study on the efficiency of Instagram in the field of education indicated that including this platform in the teaching process helped improve students writing, creativity, and story development by approaching diverse topics [74].

Line 247 Other studies also support the idea that Instagram can have positive effects on students, showing that this tool can help them socially adjust [75], and can assist them in the process of learning new languages [76].

Change to

Line 247 Other studies also support the idea that Instagram can have positive effects on students, showing that this tool can help them socially adjust [75] and can assist in the process of learning new languages [76].

Line 251 and while asked in a study [78] to rank social media apps, teachers and students mentioned Instagram as being the second social network that they use most, after WhatsApp and Facebook.

Change to

Line 251; when asked in a study [78] to rank social media apps, teachers and students mentioned Instagram as being the second social network that they use most, after WhatsApp and Facebook.

Line 253 Thus, according to previous studies, Instagram is used in education as a tool for improving student’s ability to write, to learn a new languages, and further research can be done regarding its use in the teaching and learning process.

Chang to

Line 253 Thus, according to previous studies, Instagram is used in education as a tool for improving student’s ability to write, to learn a new languages, and aid in research development.

Line 268 708 (81.2%) students are in the Bachelor Primary 267 Education program, 135 (15.5%) are enrolled in a Master programme and 29 (3.3%) are in a PhD 268 programme.

Change to

Line 268 708 Should not start a sentence with a number-A total of 708 (81.2%) students are in the Bachelor Primary 267 Education program, 135 (15.5%) are enrolled in a Master program, and 29 (3.3%) are in a PhD program.

Line 269 847 (97.1%) students have a Facebook account and 86.7% use daily their Facebook account.

Change to

Line 269 The selected 847 (97.1%) students have a Facebook account, and 86.7% use daily their Facebook account.

Line 270 Also, 760 (87.2%) students have an Instagram account and 84.7% of them use daily their 13 Instagram account.

Change to

Line 270 Also, 760 (87.2%) students have an Instagram account, and 84.7% of them use daily their 13 Instagram account.

Line 271 These information, together with other characteristics of the participants are presented in Table 1.

Change to

Line 271 This information, together with other characteristics of the participants, are presented in Table 1.

Line 276 Having as a frame of reference, a series of instruments previously used by researchers in order to analyze students’ attitude towards the use of Facebook and Instagram in general and in the academic field in particular, [79, 80, 81, 82], we developed a questionnaire through which we measured the attitude of students towards the use of the these two platforms

Change to

Line 276 Based on the data gleaned and a series of mechanisms previously used by researchers in order to analyze students’ attitude towards the use of Facebook and Instagram in general, [79, 80, 81, 82], we developed a questionnaire that measured the attitude of students towards the use of the these two platforms in the educational process.

Line 282 In this regard, the questionnaire comprises four scales, two for each of the social networks analyzed, scales that focused on the use of Facebook and Instagram in the online teaching and learning process by teachers and students.

Change to

Line 282 The questionnaire is comprised of four scales, two for each of the social networks analyzes, scales that focused on the use of Facebook and Instagram in the online teaching and learning process by teachers and students.

Line 285 Change to: All students understood the questions, and they did not find the questions difficult to answer.

Line 286 However, on the basis of their comments, we reformulated, inserted or deleted certain items.

Change to

Line 286 However, based on their feedback, we reformulated questions by inserted or deleted certain elements.

Line 290 Ranging from 1 (“disagree very strongly”) to 7 (“agree very strongly”), and each respondent had to evaluate, express its level of agreement with respect to the item which described a specific context in which Facebook and Instagram could be used for academic purposes (‘‘Please indicate how much you agree or disagree with the following statements’’).

Change to

Line 290 Ranging from 1 (“disagree very strongly”) to 7 (“agree very strongly”), and each respondent had to evaluate, express their level of agreement with respect to questions which described a specific context in which Facebook and Instagram could be used for academic purposes (‘‘Please indicate how much you agree or disagree with the following statements’’).

Line 293 The last part of the questionnaire comprises a series of socio-demographic variables: gender, degree level, field of study, owning a Facebook and Instagram account, the frequency of using the platforms

Change to

Line 293 The last part of the questionnaire was comprised of a series of socio-demographic variables: gender, degree level, field of study, and the frequency of using Facebook and Instagram platforms

Line 298 The data were analyzed using IBM SPSS Statistics 23

Change to

Line 298 The data was analyzed using IBM SPSS Statistics 23

Line 299 indicated that the data for each of the four instruments were suitable for two factor analysis

Change to

Line 299 indicated that the data for each of the four mechanisms were suitable for two factor analysis

Line 305 Some indicators were removed, and the final versions of the scales are presented in S1_ questionnaire.

Why were they removed?

Line 306 For each scale the factors were labeled: The use of social networks during the didactic activity, The use of social networks in extracurricular activities and career development (see Table 2).

Change to

Line 306 For each scale the factors were labeled: The use of social networks during the didactic activity, The use of social networks in extracurricular activities and career development, to name a few (see Table 2).

Line 324 We considered opportune and appropriate to compare respondents according to their gender, due to the fact that, generally, in many countries as well as in Romania, it is known that women are more active users of social networks

Change to

Line 324 We considered it appropriate to compare respondents according to their gender, because, generally, in many countries (as well as in Romania), women are more active users of social networks.

Line 334 Even more, we considered important a comparison depending on the level of degree of the respondents (Bachelor/ Master +PhD), because the profiles of the two categories of students are different

Change to

Line 334 Even more, we considered important a comparison depending on the level of degree of the respondents (Bachelor/ Master +PhD), because the profiles of the two categories of students vary.

Line 339 A previous study conducted in Romania also pointed out that these students are fonder of using online platforms in the educational process, than

Change to

Line 339 A previous study conducted in Romania also pointed out that these students prefer using online platforms in the educational process, than

Line 347 . In this regard, we expected students who use Facebook and Instagram daily, to be more open and willing to use these social networks in the educational field, them being more familiarized with the platforms

Change to

Line 347 In this regard, we expected students who use Facebook and Instagram daily, to be more open and willing to use these social networks in the educational field, because they are more familiar with these platforms.

Line 369 In the case of using Instagram as an educational tool, students have a more reluctant attitude, them ascribing lower scores to the affirmations included in the survey in this regard.

Change to

Line 369 In the case of using Instagram as an educational tool, students have a more reluctant attitude, ascribing lower scores to the affirmations included in the survey in this regard. Also, this should not be a separate paragraph. It should be the same as the section that discusses the Instagram results.

Line 379 The same as in the case of using Facebook as a tool in the teaching and learning process, students are

Change to

Line 379 Similar to their responses for using Facebook as a tool in the teaching and learning process, students are …

Line 382 …and not such a suitable platform for unfolding the formal educational process, the didactic activity (M=4.25, SD=2.15; M=4.00 383 SD=2.18; t(871)=-12.23, p=.00, t(871)=-14.57, p=.00).

Change to

Line 382 …and not such a suitable platform for 382 unfolding the formal educational process, the didactic activity (M=4.25, SD=2.15; M=4.00 SD=2.18; t(871)=-12.23, p=.00, t(871)=-14.57, p=.00). This should be a separate sentence. “Students believe Instagram is unsuitable for unfolding the formal educational process…”

In this regard, it can be inferred that master or PhD students

Change to

In this regard, it can be inferred that master’s or Ph.D. students

Line 413 The analysis of the items corresponding to each factor of the scale (S2_EFA_Reliability_ Explained Variances), revealed certain contexts considered by students more suitable for the use of Facebook and Instagram as educational tools

This sentence is very confusing. I’m not sure what you are trying to say.

Line 418 When referring to the didactic activity, to the formal educational process, students are of the opinion that teachers could use of Facebook for offering answers to their questions regarding the projects and tasks that they must accomplish, for inviting professionals in order to carry out debates on varied subjects, for sharing some of their experience related to their professional activity, for conducting surveys/polls on certain matters that concern the development of the courses and seminars, for informing students about any type of changes regarding the courses, or the deadlines for their projects.

Change to

Line 418 In consideration to the use of social media for formalized educational purposes, students believe teachers could use Facebook for providing answers to their questions concerning class projects and tasks, inviting professionals in their respective field for debates on varied subjects, sharing their experience related to professional activity, conducting surveys/polls on certain matters that concern the development of the courses and seminars, and, finally, informing students about any type of changes regarding the courses or the deadlines for their projects.

Line 446 With regards to the use of Facebook and Instagram by students during the didactic activity, students consider these social networks to be an appropriate place for them to manage a series of situations that require interaction and group collaboration.

Change to

Line 446 In consideration to the use of Facebook and Instagram by students as a didactic activity, students consider these social networks to be an appropriate place for them to manage interaction and group collaboration.

Line 454 With regards to the use of Facebook and Instagram by students in the context of developing extracurricular activities, students believe these platforms are suitable f

Change to

Line 454 In the context of using Facebook and Instagram for 455 developing extracurricular activities, students believe these platforms are suitable…

Line 463 Thus, considering the dimensions of extracurricular activates, our research revealed that students would like for Facebook and Instagram to be used by their teachers for conducting activities that are similar to the ones students would like to conduct on these platforms.

Change to

Line 463 Thus, considering the dimensions of extracurricular activities, our research revealed that students would like for Facebook and Instagram to be used by their teachers for conducting activities that are similar to the ones students would like to conduct on these platforms.

Line 468 Previous studies on the matter of the use of social networks in higher education revealed that many universities use and integrate Facebook into the educational process

Change to

Line 468 Previous studies on social networks use in higher education revealed that many universities use and integrate Facebook into the educational process

Line 475 Hence, if other countries opt for the integration and use of social networks in the educational act [25, 54, 55, 78], the use of such platforms in higher education institutions in Romania is rather modest, and often resumes to posting administrative information

Change to

Line 475 Hence, if other countries opt for the integration and use of social networks for educational purposes [25, 54, 55, 78], the use of such platforms in higher education institutions in Romania is rather modest, and often resumes to posting administrative information.

Line 478 Thus, one of the reasons for conducting the present study is represented by the desire to carry out a research that could complement and come as a response to a similar study [56], which also focused on students’ motives for using Facebook

Change to

Line 478 Thus, one of the reasons for conducting the present study is represented by the desire to carry out a research that could complement and come as a response to a similar study [56], which also focused on students’ motives for using Facebook-this sentence is very unclear. I am not sure what you mean by this

Line 481 Change to: In this regard, our research was conducted by taking into account the perspective of students regarding the ways they would like Facebook and Instagram to be used as educational tools in general, and in the context of the pandemic in particular.-The word “regard” is used redundantly. Consider alternative words

Line 484 The study previously mentioned [56], conducted in Romania in 2011, showed that 70% of the respondents were of the opinion that Facebook was a low-cost tool that could be used to promote knowledge in higher education, but only 26,7% of them considered Facebook an appropriate instrument of change in higher education, an intrument through which changes in strategies, and attitudes during the educational process could be achieved.

Change to

Line 484 In a previously mentioned study [56], conducted in Romania in 2011, showed that 70% of the respondents were of the opinion that Facebook was a low-cost tool that could be used to promote knowledge in higher education, but only 26,7% of them considered Facebook an appropriate instrument of change in higher education, an instrument through which changes in strategies, and attitudes during the educational process could be achieved.

Line 489 However, a change for the better can be observed in the case of using Facebook for academic purposes.

Change to

Line 489 However, we believe Facebook can be more effectively used for academic purposes.

Line 506 help the socialize.

Change to

Line 506 help them socialize.

Line 506 help the socialize. Another aspect revealed by our research is that Master and PhD students

Change to

Line 506 help the socialize. Another aspect revealed by our research is that master’s and Ph.D. students

Line 507 **Change to:** tend to have a more positive attitude towards using Facebook as an educational tool, than Bachelor students.-This is nearly identical to a previously written sentence. Consider rewording, otherwise redundant.

Line 510 With respect to the use of Instagram as an educational tool, students have a more reluctant attitude towards this social network

Change to

Line 510 With respect to the use of Instagram as an educational tool, expressed reservations towards this social network platform, considering its usefulness in the educational process occasionally, o

Line 514 . From this result it can be inferred that, as found in other studies [77], Instagram too can be considered a complementary tool for the educational process

Change to

Line 514 Based on this inference, we found, like previous studies related to Instagram use for educational purposes, …

Line 515 **Change to:** In the case of Instagram, students’ attitudes do not differ depending on their level of degree. In the case of both social networks, Facebook and Instagram, students who access daily their accounts consider to a greater extent that these platforms are suitable to be used for educational purposes.-This is redundant. You need to come up with alternative connective sentences. These are used identically in two consecutive sentences.

Line 519 Thus, students may have this attitude due to the fact that, by using these 26 social networks daily, by being more familiarized with their functions, they understand better the potential of these platforms to be used for academic purposes

Change to

Line 519 Students’ attitudes towards the use of social networks for educational purposes relates to their usage and familiarity with their programs.
