# Supplementary material for: Dear student, what should I write on my wall? A case study on academic uses of Facebook and Instagram during the pandemic
Source: PLoS One. 2021 Sep 23;16(9):e0257729. doi: 10.1371/journal.pone.0257729 (PMC8459956; doi:10.1371/journal.pone.0257729)
Supplement: Attachment — Submitted filename: Response to Reviewers.docx [file pone.0257729.s007.docx]

**Rebuttal letter**

Claudiu Coman

Transilvania University of Brasov

[claudiu.coman@unitbv.ro](mailto:claudiu.coman@unitbv.ro)

Dear Sir/Madam

With this cover letter we submit the revised manuscript entitled:” Dear student, what should I write on my wall? A case study on academic uses of Facebook and Instagram during the pandemic” Claudiu Coman, Luiza Mesesan-Schmitz, Laurentiu Gabriel Tiru, Gabriela Grosseck, and Maria Cristina Bularca for publication in PLOS ONE.

We revised the manuscript according to the suggestions made by the reviewers and we also addressed the points raised by the academic editor with regard to the changes we had to make in order to meet the requirements of the journal. We thank the reviewers for the taking the time to review our article and we are very grateful for the useful suggestions and recommendations that they provided in order to improve our article. We also thank the academic editor for taking into consideration our manuscript and for pointing out the changes we had to make to our paper in order to comply with the requirements of the journal. We tried to comply as well as possible with the suggestions of the reviewers, and we also tried our best in providing answers to the points raised by the academic editor.

In this letter we describe thoroughly the changes we made to the text depending on the comments of the reviewers and on the points raised by the academic editor. Firstly, we describe how we addressed, point by point, the recommendations of the reviewers, and every change we had to make to the text in this regard. Next, we describe how we complied with the journal requirements and what changes we made according to them.

Our manuscript needed major revisions. We made all changes to the text while having active the “Track changes” function from Microsoft Word, and the option “All Markup”. Because of the changes, because we had to insert text and additional explanations, the lines of the article changed, and the revised manuscript now has line numbers that are different from our initial manuscript. In order for our changes to be best viewed, and for the lines to correspond with the lines we referred to when we addressed the recommendations of the reviewers and the requirements of the journal, we kindly mention that the manuscript that should be consulted and read first, is the revised manuscript that has active the “Track changes”, function and its option, “All Markup”.

Further, we briefly provide a summary of major changes that we made to the text, according to the suggestions of the reviewers. Reviewer 1 provided us recommendations regarding the way certain words and phrases in the text should be changed in order to improve the article. We thank Reviewer 1 for taking the time to review the article and for providing us such useful recommendations. Thus, we complied with all the suggestions made by Reviewer 1, and changed the text in the way suggested by Reviewer 1. Every change that we made in this regard, will be described later in this letter.

Reviewer 2 made 3 comments with very useful suggestions. Before providing the suggestions, Reviewer 2 mentioned that, even though he/she has certain concerns, the paper is well-written and we would like to thank Reviewer 2 for the kind words and also for the recommendations that helped us improve the paper. In the first comment, Reviewer 2 asked us why we focused our research only the social networks Facebook and Instagram, why we did not take into account other platforms such as YouTube, Tik Tok, LinkedIN, Steemit, Reasearchgate, or platforms designed for online learning such as Coursera or Skillshare. Thus, Reviewer 2 asked us to explain what are the elements that Facebook and Instagram have in order to support the online learning process and that other social networks or platforms do not have. We tried to comply with the request of the reviewer and in order to do so, we firstly briefly explained why we focused only on Facebook and Instagram. Then we explained in the text, why each of the platforms mentioned by Reviewer 2 was not the subjects of our research. After these explanations, we clearly emphasized what elements, functions, do Facebook and Instagram have for supporting online learning, elements that other platforms do not have and elements that determined use to focus our research only on these two platforms.

In the second comment, Reviewer 2 firstly pointed out that in the Introduction section we used two references that were not supportive of our arguments, and recommended us to replace them. In order to comply with this recommendation, we replaced both Reference 3 and Reference 6 with more suitable references.

In the third comment, Reviewer 2 raised the issue of social networks addiction, in general and in the academic filed in particular, and he/she asked us to address this issue in our paper in the context of using social networks for learning. The Reviewer provided us two references that we took into account by citing them in the paper, and then we explained and provided answers to each of the question that the Reviewer requested us to address in the paper, regarding the matter of social networks addiction and their use in the process of learning.

Before describing point by point, all the changes we made to the text depending on the comments of the reviewers, we would like to mention again that the changes we made can be best seen in the revised manuscript that has active the function “Track changes” and the “All markup” option.

**Our response to Reviewer 1:**

We kindly thank the reviewer for taking the time to review our article, and for providing us such useful and valuable recommendations in order to improve our article. We tried to comply with all the changes suggested by the reviewer and the text was changed in the way the reviewer recommended. We made the changes while having active the “Track changes” function from Microsoft Word, as well as its option “All markup”. Thus, the lines we will further provide in order for our changes to be viewed correctly in the revised manuscript, can be viewed while having active the “All markup” option from the “Track changes” functions in the Word document. In other words, the “All markup” option should be ticked while consulting the revised manuscript, because the option shows both the text we deleted, as well as the text we inserted. However, with the “Simple markup”, the document shows the text with the change already made, meaning that, the document is seen as if already the changes made have been accepted, the document showing only the correct form of the text that was obtained through the changes we made. Therefore, in order for our changes to be seen completely, (text we deleted/text we corrected, text that we inserted) for each of our changes, we further mentions the lines, while having active the “All Markup” option.

**Reviewer 1 comment:**

Line 61 Although E-learning platforms or social networks were used by higher education institutions in the educational process prior to the COVID 19 pandemic, due to the pandemic

Change to

Line 61 Although E-learning platforms or social networks were used by higher education institutions in the educational process prior to the COVID-19 pandemic, due to the pandemic,

**Response:**

We thank you very much for the suggestion. Firstly, we mention that due to other changes that we were required to make to the text, some line numbers in the revised manuscript changed. Thus, some lines corresponding to the text as seen by you in our initial manuscript, changed in the revised manuscript. However, in this case, line 61 as seen by you in the initial manuscript, in the revised manuscript without the “Track Changes” option active, remained line 61, and in the revised manuscript with the “Track changes” and “All markup”, option active, it became line 63 (because this option also counts the lines of the text that we deleted as well as the lines of the text that we inserted). However, because we would like you to see the change completely, to see that we deleted the old text and inserted the text suggested, we further provide the lines as seen in the revised manuscript with the “Track changes” option active.

We changed the text as the reviewer suggested, and while having active the “Track changes” function with the “All markup” option, the full change, the text we deleted and the text we inserted can be viewed at lines 63- 66.

**Reviewer 1 comment:**

Line 66 Recent studies that focused on identifying the perception of Romanian students about online learning during the pandemic [8,9], revealed less positive attitudes towards this type of learning, students considering that the online educational process has less value than the traditional one, them preferring to use E-learning platforms as a complementary method to traditional, face to face learning

Change to

Line 66 Recent studies that focused on identifying the perception of Romanian students about online learning during the pandemic [8,9], revealed less positive attitudes towards this type of learning, students considering that the online educational process has less value than the traditional one, preferring instead to use E-learning platforms as a complementary method to traditional, face-to-face learning.

**Response:**

We thank you for the suggestion. We made changes to the text as you suggested. Line 66 became line 65 in the revised manuscript without the function “Track changes” active, and it became line 69 in the revised manuscript with the function “Track changes” and the option “All markup” active.

In the revised manuscript with the “Track changes” and “All markup” option active, the full change (the text we deleted and the text we inserted) can be seen at lines 69-77.

**Reviewer 1 comment:**

Line 73 Moreover, a study conducted on Romanian students [8], also revealed that even though students mostly used E-learning platforms, in certain circumstances they would have preferred to use other online platforms in order to compensate for the disadvantages of the E-learning platform, such as technical problems, lack of proper functions that allow collaborative learning.

Change to

Line 73 Moreover, a study conducted on Romanian students [8], also revealed that even though students frequently E-learning platforms, in certain circumstances they would have preferred to use other online platforms in order to compensate for the disadvantages of the E-learning platform, such as technical problems, lack of proper functions that allow collaborative learning.

**Response:**

We thank you for the suggestion. We made changes to the text as you suggested. Line 73 remained line 73 in the revised manuscript without the function “Track changes” active, and it became line 81 in the revised manuscript with the function “Track changes” and the option “All markup” active.

In the revised manuscript with the “Track changes” and “All markup” option active, the change (the text we deleted and the text we inserted) can be seen at lines 81-89.

**Reviewer 1 comment:**

Line 85 Facebook and Instagram were the focus of our research due to the fact that they are two of the most popular and used social networks worldwide

Change to

Line 85 Facebook and Instagram were the focus of our research because they are two of the most popular and used social networks worldwide.

**Response:**

Thank you for the recommendation. We changed the text according to the recommendations. Line 85 became line 84 in the revised manuscript without the function “Track changes” active, and it became line 96 in the revised manuscript with the function “Track changes” and the option “All markup” active.

In the revised manuscript with the “Track changes” and “All markup” option active, the change (the text we deleted and the text we inserted) can be viewed at lines 96-99.

**Reviewer 1 comment:**

Line 89 these tools, what they would like their teachers to post on these platforms and what types of activities would they prefer to carry out on them

Change to

Line 89 these tools, what they would like their teachers to post on these platforms, and what types of activities would they prefer to carry out on them.

**Response:**

Thank you for the recommendation. We changed the text according to the recommendation. Line 89 remained line 89 in the revised manuscript without the function “Track changes” active, and it became line 103 in the revised manuscript with the function “Track changes” and the option “All markup” active.

In the revised manuscript with the “Track changes” and “All markup” option active, the change (the text we deleted and the text we inserted) can be seen at lines 103-106.

**Reviewer 1 comment:**

Line 145 Students no longer use social networks only for connecting and interacting with friends or relatives [30] and these sites are nowadays also adopted by teachers because teaching and learning without technology often seems uninteresting.

Change to

Line 145 Students no longer use social networks only for connecting and interacting with friends or relatives [30], and these sites are nowadays also adopted by teachers because teaching and learning without technology often seems uninteresting.

**Response:**

Thank you for the recommendation. We changed the text according to the recommendation. Line 145 is now line 142 in the revised manuscript without the function “Track changes” active, and it became line 158 in the revised manuscript with the function “Track changes” and the option “All markup” active.

In the revised manuscript with the “Track changes” and “All markup” option active, the change (the text we deleted and the text we inserted) can be viewed at lines 158-163.

**Reviewer 1 comment:**

Line 150 Even more, with the development of online courses, it is important to understand the way students think and how do they approach learning in an environment where learning is very different from how it can usually be done in traditional courses

Change to

Line 150 Even more, with the development of online courses, it is important to understand the methods students use to learn and the manner in which they think in an environment where learning is very different from how it can usually be done in traditional courses.

**Response:**

Thank you for the recommendation. We changed the text according to the recommendation. Line 150 became line 148 in the revised manuscript without the function “Track changes” active, and it became line 166 in the revised manuscript with the function “Track changes” and the option “All markup” active.

In the revised manuscript with the “Track changes” and “All markup” option active, the change (the text we deleted and the text we inserted) can be viewed at lines 166-171.

**Reviewer 1 comment:**

Line 157 When teaching online, teachers must provide clear instructions, the content of the course must be delivered in a smooth, simple manner [35], and it is not recommended to attempt to create online the traditional course, but to adapt it and focus on the benefits offered by the environment

Change to

Line 157 When teaching online, teachers must provide clear instructions, the content of the course must be delivered in a smooth, simple manner [35], and adapt it to focus on the benefits offered by the environment.

**Response:**

Thank you for the suggestion. We changed the text according to the recommendation. Line 157 became 154 in the revised manuscript without the function “Track changes” active, and it became line 175 in the revised manuscript with the function “Track changes” and the option “All markup” active-

In the revised manuscript with the “Track changes” and “All markup” option active, the change (the text we deleted and the text we inserted) can be viewed at lines 175-180.

**Reviewer 1 comment:**

Line 162 Besides being used for dissemination of information [39], social networks encourage collaboration, allowing students to share, debate and examine their ideas together with their peers and teachers and can improve students engagement, providing the opportunity to learn in an informal environment, supporting intimidated or less involved students to express their opinion in a more pleasant way.

Change to

Line 162 Besides being used for dissemination of information [39], social networks encourage collaboration, allowing students to share, debate and examine their ideas together with their peers and teachers, which can improve students engagement; providing the opportunity to learn in an informal environment; and supporting intimidated or less involved students to express their opinion in a more pleasant way.

**Response:**

Thank you for the suggestion. Regarding this suggestion we want to mention that we were not able to change the text in the way recommended, because of the changes we were required to make in in order to also comply with the recommendation of the other reviewer. We will further provide additional explanations on this matter.

Because the other reviewer of the article recommended us to replace Reference [6], and thus we tried to also comply with this recommendation, Reference [6] of the article was replaced. The reference 6, was the reference from which we obtained the information regarding other ways in which social networks can be used for educational purposes, more precisely, this information,: “social networks encourage collaboration, allowing students to share, debate and examine their ideas together with their peers and teachers and can improve students engagement, providing the opportunity to learn in an informal environment, supporting intimidated or less involved students to express their opinion in a more pleasant way.” (lines 162- 166 in the initial manuscript, and lines 188-192 in the revised manuscript with “Track changes” and All Markup active. Hence, since Reference 6 was replaced, we could not use the text that you recommended for it no longer belonged to that reference.

The change of the reference can be consulted at lines 854-858 while having active the “All Markup” option of the “Track changes” function from Microsoft Word. However, the new Reference [6], also provides information about the ways social networks such as Facebook can be used as educational tools, and thus, the text from the line that was 162 in the initial manuscript: “social networks encourage collaboration, allowing students to share, debate and examine their ideas together with their peers and teachers, which can improve students engagement; providing the opportunity to learn in an informal environment; and supporting intimidated or less involved students to express their opinion in a more pleasant way”, was deleted, and information on the same matter was added from the new Reference 6.

Thus, line 162 became line 160 in the revised manuscript without the function “Track changes” active, and it became line 184 in the revised manuscript with the function “Track changes” and the option “All markup” active.

In the revised manuscript with the “Track changes” and “All markup” option active, the change (the text we deleted and the text we inserted) can be viewed at lines 184-192.

Therefore:

Line 162 Besides being used for dissemination of information [39], social networks encourage collaboration, allowing students to share, debate and examine their ideas together with their peers and teachers and can improve students engagement, providing the opportunity to learn in an informal environment, supporting intimidated or less involved students to express their opinion in a more pleasant way.

Was changed to: Besides being used for dissemination of information [39], social networks can improve students’ level of engagement during courses, can enhance the educational process by allowing teachers to share with students varied course materials, and they can also improve collaboration between students, by allowing them to chat in real time, to offer feedback and comment on the posts of their peers.

**Reviewer 1 comment:**

Line 167 Social networks, help teachers and students maintain continuous contact while setting up new and spaces for learning, [40] they make learning interactive [41] and through them students can access, read the content taught at any date and time.

Change to

Line 167 Social networks, help teachers and students maintain continuous contact while setting up new and spaces for learning; [40] they make learning interactive [41], and through them students can access and read the content taught at any date and time.

**Response:**

Thank you for the recommendation. We changed the text according to the recommendation. Line 167 became line 164 in the revised manuscript without the function “Track changes” active, and it became line 192 in the revised manuscript with the function “Track changes” and the option “All markup” active.

In the revised manuscript with the “Track changes” and “All markup” option active, the change (the text we deleted and the text we inserted) can be viewed at lines 192-197.

**Reviewer 1 comment:**

Line 171 Studies [42,43] on the use of social networks and their impact on student’s grades showed that they negatively affect student’s academic performance, those using platforms such as Facebook, registering lower grade-point averages, while other studies [44,45], found no connection or significant relationship between the amount of time spent on social networks and grades, thus providing contrasting results on this matter.

Change to

Line 171 Studies [42,43] on the use of social networks and their impact on student’s grades showed that they negatively affect student’s academic performance. Those using platforms such as Facebook, registering lower grade-point averages, while other studies [44,45], found no connection or significant relationship between the amount of time spent on social networks and grades, thus providing contrasting results on this matter.

**Response:**

Thank you for the recommendation. We changed the text according to the recommendation. Line 171 became line 168 in the revised manuscript without the function “Track changes” active, and it became line 198 in the revised manuscript with the function “Track changes” and the option “All markup” active.

In the revised manuscript with the “Track changes” and “All markup” option active, the change (the text we deleted and the text we inserted) can be viewed at lines 198-208.

**Reviewer 1 comment:**

Line 178 It is a fact that Facebook is the most popular and largest social network nowadays, reaching a wide number of people that use it for multiple purposes.

Change to

Line 178 It is a fact that Facebook is the most popular and largest social network nowadays, reaching a wide number of people who use it for multiple purposes.

**Response:**

Thank you for the recommendation. We changed the text according to the recommendation. Line 178 became line 175 in the revised manuscript without the function “Track changes” active, and it became line 213 in the revised manuscript with the function “Track changes” and the option “All markup” active.

In the revised manuscript with the “Track changes” and “All markup” option active, the change (the text we deleted and the text we inserted) can be viewed at lines 213-216.

**Reviewer 1 comment:**

Line 180 According to a study on the global internet use, in January 2020 there were 3,8 billion social media users and Facebook, with 2,44 billion monthly users, remains the social network most used worldwide.

Change to

Line 180 According to a study on the global internet use, in January 2020 there were 3,8 billion social media users and Facebook, with 2,44 billion monthly users. It remains the social network most used worldwide.

**Response:**

Thank you for the recommendation. We changed the text according to the recommendation, but instead of the word with, we used the word having. Thus the expression “…Facebook, with 2,44 billion monthly users", was written in the following way “…Facebook, having 2,44 billion monthly users." Line 180 became line 176 in the revised manuscript without the function “Track changes” active, and it became line 218 in the revised manuscript with the function “Track changes” and the option “All markup” active.

In the revised manuscript with the “Track changes” and “All markup” option active, the change (the text we deleted and the text we inserted) can be viewed at lines 218-220.

**Reviewer 1 comment:**

Line 182 Facebook was originally created within Harvard University’s grounds,[46] being restricted only to Harvard students, allowing them to create personal profiles, interact, socialize and contact other students

Change to

Line 182 Facebook was originally created within Harvard University’s grounds, [46] restricted only to Harvard students, and allowing them to create personal profiles, interact, socialize, and contact other students

**Response:**

Thank you for the recommendation. We changed the text according to the recommendation. Line 182 became line 179 in the revised manuscript without the function “Track changes” active, and it became line 221 in the revised manuscript with the function “Track changes” and the option “All markup” active.

In the revised manuscript with the “Track changes” and “All markup” option active, the change (the text we deleted and the text we inserted) can be viewed at lines 221-225.

**Reviewer 1 comment:**

Line 186 From a student’s perspective, teachers could use Facebook for creating groups where presentations related to the lectures could be posted, as well as information about different changes with respect to the development of the courses

Change to

Line 186 From a student’s perspective, teachers could use Facebook for creating groups where presentations related to the lectures could be posted, as well as provide information about different changes with respect to the development of the courses.

**Response:**

Thank you for the recommendation. We changed the text in order to comply with it. Line 186 became 182 in the revised manuscript without the function “Track changes” active, and it became line 227 in the revised manuscript with the function “Track changes” and the option “All markup” active.

In the revised manuscript with the “Track changes” and “All markup” option active, the change (the text we deleted and the text we inserted) can be viewed at lines 227-232.

**Reviewer 1 comment:**

Line 190 between them and the university, thus students being more willing to study and complete their degree at their current university

Change to

Line 190 between them and the university, thus students are more willing to study and complete their degree at their current university.

**Response:**

Thank you for the recommendation. We changed the text according to the recommendation. Line 190 became line 187 in the revised manuscript without the function “Track changes” active, and it became line 234 in the revised manuscript with the function “Track changes” and the option “All markup” active.

In the revised manuscript with the “Track changes” and “All markup” option active, the change (the text we deleted and the text we inserted) can be viewed at lines 234-236.

**Reviewer 1 comment:**

Line 201 much more proper medium for social learning than the real one, they preferring to receive tasks on this platform and stating that they favor discovering, searching and accomplishing school tasks through this platform, for it makes them feel more comfortable.

Change to

Line 201 much more proper medium for social learning than the real one; they preferring to receive tasks on this platform and, because they prefer this medium for discovering information, accomplishing school tasks through this platform.

**Response:**

We changed the text according to the recommendation. Line 201 became line 198 in the revised manuscript without the function “Track changes” active, and it became line 246 in the revised manuscript with the function “Track changes” and the option “All markup” active.

In the revised manuscript with the “Track changes” and “All markup” option active, the change (the text we deleted and the text we inserted) can be viewed at lines 246-251.

**Reviewer 1 comment:**

Line 206 accommodate to university life

Change to

Line 206 acclimate to university life.

**Response:**

We changed the text according to the recommendation. Line 206 is now line 204 in the revised manuscript without the function “Track changes” active, and it became line 253 in the revised manuscript with the function “Track changes” and the option “All markup” active.

In the revised manuscript with the “Track changes” and “All markup” option active, the change (the text we deleted and the text we inserted) can be viewed at lines 253-254.

**Reviewer 1 comment:**

Line 207 Even more, a large amount of students access Facebook in order to find out more information about people they had recently met, to stay in touch with old and present friends [59],

Change to

Line 207 Even more, a large number of students access Facebook in order to find out more information about people they had recently met and to stay in touch with old and present friends [59]; additional studies [60] show that students would rather interact with friends on public wall spaces, with this type of communication being twice as frequent as exchanging information through private messages, over personal or face-to-face interaction.

**Response:**

We changed the text according to the recommendation. Line 207 became line 203 in the revised manuscript without the function “Track changes” active, and it became line 254 in the revised manuscript with the function “Track changes” and the option “All markup” active.

In the revised manuscript with the “Track changes” and “All markup” option active, the change (the text we deleted and the text we inserted) can be viewed at lines 254-263.

**Reviewer 1 comment:**

Line 211 In addition, it is taught that Facebook is the social network best suited for students [61], and this affirmation is sustained by a study which included students using a Facebook group for academic purposes that uncovered the fact that all students confirmed an improved interaction with their teachers, concluding that the use of social networks could refine their learning experience [62].

Change to

Line 211 In addition, it is thought that Facebook is the social network best suited for students [61], This statement is sustained by a study which included students using a Facebook group for academic purposes confirmed an improved student interaction with their teachers, and, thus, concluding that the use of social networks could refine their learning experience [62].

**Response:**

We changed the text according to the recommendation. Line 211 became line 207 in the revised manuscript without the function “Track changes” active, and it became line 263 in the revised manuscript with the function “Track changes” and the option “All markup” active.

In the revised manuscript with the “Track changes” and “All markup” option active, the full change (the text we deleted and the text we inserted) can be viewed at lines 263-271.

**Reviewer 1 comment:**

Line 219 Recognizing the educational potential of Facebook, teachers must find ways to efficiently use it and to understand how to interact with students so as to create positive attitudes towards this type of learning

Change to

Line 219 Recognizing the educational potential of Facebook, teachers must find ways to efficiently use it and understand how to interact with students, so they create positive attitudes towards this type of learning.

**Response:**

We changed the text according to the recommendation. Line 219 became line 215 in the revised manuscript without the function “Track changes” active, and it became line 275 in the revised manuscript with the function “Track changes” and the option “All markup” active.

In the revised manuscript with the “Track changes” and “All markup” option active, the full change (the text we deleted and the text we inserted) can be viewed at lines 275-279.

**Reviewer 1 comment:**

Line 226 Instagram is a photo-video sharing platform launched in 2010, currently owned by Facebook, that allows users to post visual content, 24 hours stories, and privately communicate with their friends, the platform registering in 2020 over 1 billion monthly active users.

Change to

Line 226 Instagram is a photo-video sharing platform launched in 2010, currently owned by Facebook, that allows users to post visual content, 24 hours stories, and privately communicate with their friends. The platform registering in 2020 over 1 billion monthly active users.

**Response:**

We changed the text according to the recommendation. Line 226 became line 223 in the revised manuscript without the function “Track changes” active, and it became line 286 in the revised manuscript with the function “Track changes” and the option “All markup” active.

In the revised manuscript with the “Track changes” and “All markup” option active, the full change (the text we deleted and the text we inserted) can be viewed at lines 286-291.

**Reviewer 1 comment:**

Line 230 Instagram is especially designed for mobile use and it’s a platform more orientated to mobile experience, one of the reasons for its rapid ascension being the development of smartphones [68], and the excessively use of them.

Change to

Line 230 Instagram is especially designed for mobile use; it is a platform more orientated to a mobile experience. One of the reasons for its rapid ascension is the development and widespread use of smartphones.

**Response:**

We changed the text according to the recommendation. Line 230 became line 226 in the revised manuscript without the function “Track changes” active, and it became line 292 in the revised manuscript with the function “Track changes” and the option “All markup” active.

In the revised manuscript with the “Track changes” and “All markup” option active, the full change (the text we deleted and the text we inserted) can be viewed at lines 292-296.

**Reviewer 1 comment:**

Line 232 However, Instagram is about more than just photo sharing, the platform offering the possibility to create communities in many domains, with users being able to broaden their contact list, exchange information, share knowledge through public or private messages, live videos

Change to

Line 232 However, Instagram is about more than just photo sharing. The platform offers the possibility to create communities in many domains, and the ability for users to broaden their contact list, exchange information, share knowledge through public or private messages, live videos.

**Response:**

We changed the text according to the recommendation. Line 232 became line 228 in the revised manuscript without the function “Track changes” active, and it became line 296 in the revised manuscript with the function “Track changes” and the option “All markup” active.

In the revised manuscript with the “Track changes” and “All markup” option active, the full change (the text we deleted and the text we inserted) can be viewed at lines 296-302.

**Reviewer 1 comment:**

Line 235 Even more, through its emphasis on visual content, and through the actions of the users: taking the photo, editing it, writing a description, Instagram can help improve student’s spatial or linguistic intelligence

Change to

Line 235 Additionally, through its emphasis on visual content, and through the actions of the users (taking the photo, editing a photo, and writing a description) Instagram can help improve student’s spatial or linguistic intelligence.

**Response:**

We changed the text according to the recommendation. Line 235 became line 231 in the revised manuscript without the function “Track changes” active, and it became line 302 in the revised manuscript with the function “Track changes” and the option “All markup” active.

In the revised manuscript with the “Track changes” and “All markup” option active, the full change (the text we deleted and the text we inserted) can be viewed at lines 302-307.

**Reviewer 1 comment:**

Line 237 While students today are considered digital natives who heavily use social networks, [71], professors should integrate such networks as teaching tools, in order to enhance their learning experience, giving them the chance to share ideas and opinions through a medium that they consider pleasant and interesting [72].

Changes

Line 237 This should be added to the previous paragraph. Also, change to: While students today are considered digital natives who heavily use social networks,[71], professors should integrate such networks as teaching tools in order to enhance their learning experience and give students the chance to share ideas and opinions through a medium that they consider pleasant and interesting [72].

**Response:**

We changed the text according to the recommendation. The text was added to the previous paragraph. Line 237 became line 233 in the revised manuscript without the function “Track changes” active, and it became line 308 in the revised manuscript with the function “Track changes” and the option “All markup” active.

In the revised manuscript with the “Track changes” and “All markup” option active, the full change (the text we deleted and the text we inserted) can be viewed at lines 308-314.

**Reviewer 1 comment:**

Line 244 A previous study on the efficiency of Instagram in the field of education indicated that, including this platform in the teaching process helped improve students writing, them being more creative, developing the story in a more attractive way by approaching diverse topics [74].

Change to

Line 244 A previous study on the efficiency of Instagram in the field of education indicated that including this platform in the teaching process helped improve students writing, creativity, and story development by approaching diverse topics [74].

**Response:**

We changed the text according to the recommendation. Line 244 became line 240 in the revised manuscript without the function “Track changes” active, and it became line 317 in the revised manuscript with the function “Track changes” and the option “All markup” active.

In the revised manuscript with the “Track changes” and “All markup” option active, the full change (the text we deleted and the text we inserted) can be viewed at lines 317-322.

**Reviewer 1 comment:**

Line 247 Other studies also support the idea that Instagram can have positive effects on students, showing that this tool can help them socially adjust [75], and can assist them in the process of learning new languages [76].

Change to

Line 247 Other studies also support the idea that Instagram can have positive effects on students, showing that this tool can help them socially adjust [75] and can assist in the process of learning new languages [76].

**Response:**

We changed the text according to the recommendation. Line 247 became line 242 in the revised manuscript without the function “Track changes” active, and it became line 317 in the revised manuscript with the function “Track changes” and the option “All markup” active.

In the revised manuscript with the “Track changes” and “All markup” option active, the full change (the text we deleted and the text we inserted) can be viewed at lines 322-327.

**Reviewer 1 comment:**

Line 251 and while asked in a study [78] to rank social media apps, teachers and students mentioned Instagram as being the second social network that they use most, after WhatsApp and Facebook.

Change to

Line 251; when asked in a study [78] to rank social media apps, teachers and students mentioned Instagram as being the second social network that they use most, after WhatsApp and Facebook.

**Response:**

We changed the text according to the recommendation. Line 251 became line 246 in the revised manuscript without the function “Track changes” active, and it became line 317 in the revised manuscript with the function “Track changes” and the option “All markup” active.

In the revised manuscript with the “Track changes” and “All markup” option active, the full change (the text we deleted and the text we inserted) can be viewed at lines 328-332.

**Reviewer 1 comment:**

Line 253 Thus, according to previous studies, Instagram is used in education as a tool for improving student’s ability to write, to learn a new languages, and further research can be done regarding its use in the teaching and learning process.

Change to

Line 253 Thus, according to previous studies, Instagram is used in education as a tool for improving student’s ability to write, to learn a new languages, and aid in research development.

**Response:**

We changed the text according to the recommendation. Line 253 became line 248 in the revised manuscript without the function “Track changes” active, and it became line 332 in the revised manuscript with the function “Track changes” and the option “All markup” active.

In the revised manuscript with the “Track changes” and “All markup” option active, the full change (the text we deleted and the text we inserted) can be viewed at lines 332-337.

**Reviewer 1 comment:**

Line 268 708 (81.2%) students are in the Bachelor Primary 267 Education program, 135 (15.5%) are enrolled in a Master programme and 29 (3.3%) are in a PhD 268 programme.

Change to

Line 268 708 Should not start a sentence with a number-A total of 708 (81.2%) students are in the Bachelor Primary 267 Education program, 135 (15.5%) are enrolled in a Master program, and 29 (3.3%) are in a PhD program.

**Response:**

We changed the text according to the recommendation. Line 268 became line 262 in the revised manuscript without the function “Track changes” active, and it became line 349 in the revised manuscript with the function “Track changes” and the option “All markup” active.

In the revised manuscript with the “Track changes” and “All markup” option active, the full change (the text we deleted and the text we inserted) can be viewed at lines 349-352.

**Reviewer 1 comment:**

Line 269 847 (97.1%) students have a Facebook account and 86.7% use daily their Facebook account.

Change to

Line 269 The selected 847 (97.1%) students have a Facebook account, and 86.7% use daily their Facebook account.

**Response:**

We changed the text according to the recommendation. Line 269 became line 264 in the revised manuscript without the function “Track changes” active, and it became line 352 in the revised manuscript with the function “Track changes” and the option “All markup” active.

In the revised manuscript with the “Track changes” and “All markup” option active, the full change (the text we deleted and the text we inserted) can be viewed at lines 352-354.

**Reviewer 1 comment:**

Line 270 Also, 760 (87.2%) students have an Instagram account and 84.7% of them use daily their 13 Instagram account.

Change to

Line 270 Also, 760 (87.2%) students have an Instagram account, and 84.7% of them use daily their 13 Instagram account.

**Response:**

We changed the text according to the recommendation. Line 270 became line 265 in the revised manuscript without the function “Track changes” active, and it became line 354 in the revised manuscript with the function “Track changes” and the option “All markup” active.

In the revised manuscript with the “Track changes” and “All markup” option active, the full change (the text we deleted and the text we inserted) can be viewed at lines 354-357.

**Reviewer 1 comment:**

Line 271 These information, together with other characteristics of the participants are presented in Table 1.

Change to

Line 271 This information, together with other characteristics of the participants, are presented in Table 1.

**Response:**

We changed the text according to the recommendation. Line 271 became line 266 in the revised manuscript without the function “Track changes” active, and it became line 357 in the revised manuscript with the function “Track changes” and the option “All markup” active.

In the revised manuscript with the “Track changes” and “All markup” option active, the full change (the text we deleted and the text we inserted) can be viewed at lines 357-359.

**Reviewer 1 comment:**

Line 276 Having as a frame of reference, a series of instruments previously used by researchers in order to analyze students’ attitude towards the use of Facebook and Instagram in general and in the academic field in particular, [79, 80, 81, 82], we developed a questionnaire through which we measured the attitude of students towards the use of the these two platforms

Change to

Line 276 Based on the data gleaned and a series of mechanisms previously used by researchers in order to analyze students’ attitude towards the use of Facebook and Instagram in general, [79, 80, 81, 82], we developed a questionnaire that measured the attitude of students towards the use of the these two platforms in the educational process.

**Response:**

We changed the text according to the recommendation. Line 276 became line 272 in the revised manuscript without the function “Track changes” active, and it became line 364 in the revised manuscript with the function “Track changes” and the option “All markup” active.

In the revised manuscript with the “Track changes” and “All markup” option active, the full change (the text we deleted and the text we inserted) can be viewed at lines 364-371.

**Reviewer 1 comment:**

Line 282 In this regard, the questionnaire comprises four scales, two for each of the social networks analyzed, scales that focused on the use of Facebook and Instagram in the online teaching and learning process by teachers and students.

Change to

Line 282 The questionnaire is comprised of four scales, two for each of the social networks analyzes, scales that focused on the use of Facebook and Instagram in the online teaching and learning process by teachers and students.

**Response:**

We changed the text according to the recommendation. Line 282 became line 277 in the revised manuscript without the function “Track changes” active, and it became line 373 in the revised manuscript with the function “Track changes” and the option “All markup” active.

In the revised manuscript with the “Track changes” and “All markup” option active, the full change (the text we deleted and the text we inserted) can be viewed at lines 373-378.

**Reviewer 1 comment:**

Line 285 Change to: All students understood the questions, and they did not find the questions difficult to answer.

**Response:**

We changed the text according to the recommendation. Line 285 became line 280 in the revised manuscript without the function “Track changes” active, and it became line 379 in the revised manuscript with the function “Track changes” and the option “All markup” active.

In the revised manuscript with the “Track changes” and “All markup” option active, the full change (the text we deleted and the text we inserted) can be viewed at lines 379-381.

**Reviewer 1 comment:**

Line 286 However, on the basis of their comments, we reformulated, inserted or deleted certain items.

Change to

Line 286 However, based on their feedback, we reformulated questions by inserted or deleted certain elements.

**Response:**

We changed the text according to the recommendation. Line 286 became line 281 in the revised manuscript without the function “Track changes” active, and it became line 381 in the revised manuscript with the function “Track changes” and the option “All markup” active.

In the revised manuscript with the “Track changes” and “All markup” option active, the full change (the text we deleted and the text we inserted) can be viewed at lines 381-383.

**Reviewer 1 comment:**

Line 290 Ranging from 1 (“disagree very strongly”) to 7 (“agree very strongly”), and each respondent had to evaluate, express its level of agreement with respect to the item which described a specific context in which Facebook and Instagram could be used for academic purposes (‘‘Please indicate how much you agree or disagree with the following statements’’).

Change to

Line 290 Ranging from 1 (“disagree very strongly”) to 7 (“agree very strongly”), and each respondent had to evaluate, express their level of agreement with respect to questions which described a specific context in which Facebook and Instagram could be used for academic purposes (‘‘Please indicate how much you agree or disagree with the following statements’’).

**Response:**

We changed the text according to the recommendation. Line 290 became line 285 in the revised manuscript without the function “Track changes” active, and it became line 385 in the revised manuscript with the function “Track changes” and the option “All markup” active.

In the revised manuscript with the “Track changes” and “All markup” option active, the full change (the text we deleted and the text we inserted) can be viewed at lines 385-393.

**Reviewer 1 comment:**

Line 293 The last part of the questionnaire comprises a series of socio-demographic variables: gender, degree level, field of study, owning a Facebook and Instagram account, the frequency of using the platforms

Change to

Line 293 The last part of the questionnaire was comprised of a series of socio-demographic variables: gender, degree level, field of study, and the frequency of using Facebook and Instagram platforms

**Response:**

We changed the text according to the recommendation. Line 293 became line 288 in the revised manuscript without the function “Track changes” active, and it became line 393 in the revised manuscript with the function “Track changes” and the option “All markup” active.

In the revised manuscript with the “Track changes” and “All markup” option active, the full change (the text we deleted and the text we inserted) can be viewed at lines 393-397.

**Reviewer 1 comment:**

Line 298 The data were analyzed using IBM SPSS Statistics 23

Change to

Line 298 The data was analyzed using IBM SPSS Statistics 23

**Response:**

We changed the text according to the recommendation. Line 298 became line 293 in the revised manuscript without the function “Track changes” active, and it became line 400 in the revised manuscript with the function “Track changes” and the option “All markup” active.

In the revised manuscript with the “Track changes” and “All markup” option active, the full change (the text we deleted and the text we inserted) can be viewed at lines 400-401.

**Reviewer 1 comment:**

Line 299 indicated that the data for each of the four instruments were suitable for two factor analysis

Change to

Line 299 indicated that the data for each of the four mechanisms were suitable for two factor analysis

**Response:**

We changed the text according to the recommendation. Line 299 became line 294 in the revised manuscript without the function “Track changes” active, and it became line 401 in the revised manuscript with the function “Track changes” and the option “All markup” active.

In the revised manuscript with the “Track changes” and “All markup” option active, the full change (the text we deleted and the text we inserted) can be viewed at lines 401-403.

**Reviewer 1 comment:**

Line 305 Some indicators were removed, and the final versions of the scales are presented in S1_ questionnaire.

Why were they removed?

**Response:**

Thank you for raising this question and for suggesting us to provide an explanation for why we removed some of the items. Firstly, we want to mention that, because in our revised manuscript we also had to comply with some requirements of the journal, the name of the document S1_ questionnaire was changed to S1 Initial versions of the scales.

In the revised manuscript we explained that we had two reasons for removing some items (f23p., f24p., f20.p, f21p., f19s, f21s., i17p., i18,p., i17.s) The first reasons is related to the fact that the correlation of the coefficients of the items was very low, and the second reason was related to the fact that the indicators we removed are correlated with both factors extracted, but it is also related to the fact that the indicators were formulated generally.

Line 305 became line 299 in the revised manuscript without the function “Track changes” active, and it became line 408 in the revised manuscript with the function “Track changes” and the option “All markup” active.

Hence, we inserted in our paper the following explanation:

“Some indicators were removed (f20.p, f21.p; f19.s, f21.s; i8.p, i16.p, i17.p, i18.p, i20.p, i17.s), and we had two reasons for removing them. One of the reasons is related to the correlation of the coefficients of items with the factors, which are nearby 0.60 or lower. Another reason is related to the fact that these indicators are correlated with both factors extracted (the correlation coefficients are approximately equal). Looking more closely at these items we see that they are formulated generally, and because of these, students may have different representations to which to refer. Therefore, initial versions of the scales are presented in “S1 Initial versions of the scales”, and the final versions of scales are presented in “S2 EFA Reliability Explained Variances”.

In the revised manuscript with the “Track changes” and “All markup” option active, the full change, (the text we deleted and the text we inserted) can be viewed at lines 408-417.

In the revised manuscript without the function “Track changes” active, the explanation can be viewed at lines 299-307. In addition, in our supporting information document S1 initial versions of the scales, we also highlighted the items that we removed.

f20.p Facebook in an appropriate platform for teachers to maintain connection with students

f21.p Facebook is an appropriate platform for teachers to maintain connection with graduates

f23.p Facebook is an appropriate platform for teachers to communicate with students about certain personal problems that have an impact on their academic performance

f24.p Facebook is an appropriate platform for teachers to manage o series of situation that requires group interaction

f19.s Facebook is an appropriate platform for students to manage a series of situations that require interaction and group consensus

f21.s Facebook is an appropriate platform for students to post information about the projects they are involved in

i17p. Instagram is an appropriate platform for teachers to maintain connection with students

i18p. Instagram is an appropriate platform for teachers to maintain connection with graduates

i17s. Instagram is an appropriate platform for students to post information about the projects they are involved in

**Reviewer 1 comment:**

Line 306 For each scale the factors were labeled: The use of social networks during the didactic activity, The use of social networks in extracurricular activities and career development (see Table 2).

Change to

Line 306 For each scale the factors were labeled: The use of social networks during the didactic activity, The use of social networks in extracurricular activities and career development, to name a few (see Table 2).

**Response:**

We changed the text according to the recommendation. Line 306 became line 307 in the revised manuscript without the function “Track changes” active, and it became line 417 in the revised manuscript with the function “Track changes” and the option “All markup” active.

In the revised manuscript with the “Track changes” and “All markup” option active, the full change (the text we deleted and the text we inserted) can be viewed at lines 417-421.

**Reviewer 1 comment:**

Line 324 We considered opportune and appropriate to compare respondents according to their gender, due to the fact that, generally, in many countries as well as in Romania, it is known that women are more active users of social networks

Change to

Line 324 We considered it appropriate to compare respondents according to their gender, because, generally, in many countries (as well as in Romania), women are more active users of social networks.

**Response:**

We changed the text according to the recommendation. Line 324 became line 325 in the revised manuscript without the function “Track changes” active, and it became line 417 in the revised manuscript with the function “Track changes” and the option “All markup” active.

In the revised manuscript with the “Track changes” and “All markup” option active, the full change (the text we deleted and the text we inserted) can be viewed at lines 438-442.

**Reviewer 1 comment:**

Line 334 Even more, we considered important a comparison depending on the level of degree of the respondents (Bachelor/ Master +PhD), because the profiles of the two categories of students are different

Change to

Line 334 Even more, we considered important a comparison depending on the level of degree of the respondents (Bachelor/ Master +PhD), because the profiles of the two categories of students vary.

**Response:**

We changed the text according to the recommendation. Line 334 remained line 334 in the revised manuscript without the function “Track changes” active, and it became line 450 in the revised manuscript with the function “Track changes” and the option “All markup” active.

In the revised manuscript with the “Track changes” and “All markup” option active, the full change (the text we deleted and the text we inserted) can be viewed at lines 450-454.

**Reviewer 1 comment:**

Line 339 A previous study conducted in Romania also pointed out that these students are fonder of using online platforms in the educational process, than

Change to

Line 339 A previous study conducted in Romania also pointed out that these students prefer using online platforms in the educational process, than

**Response:**

We changed the text according to the recommendation, but after the word “process,” we also added the word “more”, in order to clarify better that master and phd students prefer more to use online platforms than bachelor students.

Thus the initial text was changed to: “A previous study conducted in Romania also pointed out that these students prefer using online platforms in the educational process, more than

Line 339 became line 338 in the revised manuscript without the function “Track changes” active, and it became line 456 in the revised manuscript with the function “Track changes” and the option “All markup” active.

In the revised manuscript with the “Track changes” and “All markup” option active, the full change (the text we deleted and the text we inserted) can be viewed at lines 456-459.

**Reviewer 1 comment:**

Line 347 . In this regard, we expected students who use Facebook and Instagram daily, to be more open and willing to use these social networks in the educational field, them being more familiarized with the platforms

Change to

Line 347 In this regard, we expected students who use Facebook and Instagram daily, to be more open and willing to use these social networks in the educational field, because they are more familiar with these platforms.

**Response:**

We changed the text according to the recommendation. Line 347 became line 346 in the revised manuscript without the function “Track changes” active, and it became line 465 in the revised manuscript with the function “Track changes” and the option “All markup” active.

In the revised manuscript with the “Track changes” and “All markup” option active, the full change (the text we deleted and the text we inserted) can be viewed at lines 465-469.

**Reviewer 1 comment:**

Line 369 In the case of using Instagram as an educational tool, students have a more reluctant attitude, them ascribing lower scores to the affirmations included in the survey in this regard.

Change to

Line 369 In the case of using Instagram as an educational tool, students have a more reluctant attitude, ascribing lower scores to the affirmations included in the survey in this regard. Also, this should not be a separate paragraph. It should be the same as the section that discusses the Instagram results.

**Response:**

We changed the text according to the recommendation. Also, the text is no longer a separate paragraph, we added the text to section that discusses the Instagram results. Line 369 became line 368 in the revised manuscript without the function “Track changes” active, and it became line in the revised manuscript with the function “Track changes” and the option “All markup” active.

In the revised manuscript with the “Track changes” and “All markup” option active, the full change (the text we deleted and the text we inserted) can be viewed at lines 490-493.

**Reviewer 1 comment:**

Line 379 The same as in the case of using Facebook as a tool in the teaching and learning process, students are

Change to

Line 379 Similar to their responses for using Facebook as a tool in the teaching and learning process, students are …

**Response:**

We changed the text according to the recommendation. Line 379 became line 378 in the revised manuscript without the function “Track changes” active, and it became line 501 in the revised manuscript with the function “Track changes” and the option “All markup” active.

In the revised manuscript with the “Track changes” and “All markup” option active, the full change (the text we deleted and the text we inserted) can be viewed at lines 501-503.

**Reviewer 1 comment:**

Line 382 …and not such a suitable platform for unfolding the formal educational process, the didactic activity (M=4.25, SD=2.15; M=4.00 383 SD=2.18; t(871)=-12.23, p=.00, t(871)=-14.57, p=.00).

Change to

Line 382 …and not such a suitable platform for unfolding the formal educational process, the didactic activity (M=4.25, SD=2.15; M=4.00 SD=2.18; t(871)=-12.23, p=.00, t(871)=-14.57, p=.00). This should be a separate sentence. “Students believe Instagram is unsuitable for unfolding the formal educational process…”

**Response:**

We changed the text according to the recommendation, the information was written as a separate sentence. Line 382 became line 381 in the revised manuscript without the function “Track changes” active, and it became line 505 in the revised manuscript with the function “Track changes” and the option “All markup” active.

In the revised manuscript with the “Track changes” and “All markup” option active, the full change (the text we deleted and the text we inserted) can be viewed at lines 505-509.

**Reviewer 1 comment:**

In this regard, it can be inferred that master or PhD students

Change to

In this regard, it can be inferred that master’s or Ph.D. students

**Response:**

We changed the text according to the recommendation. In the revised manuscript with the “Track changes” and “All markup” option active, the full change (the text we deleted and the text we inserted) can be viewed at lines 518-519, and in the revised manuscript without the function “Track changes” active, the correct text can be viewed at line 392.

**Reviewer 1 comment:**

Line 413 The analysis of the items corresponding to each factor of the scale (S2_EFA_Reliability_ Explained Variances), revealed certain contexts considered by students more suitable for the use of Facebook and Instagram as educational tools

This sentence is very confusing. I’m not sure what you are trying to say.

**Response:** In the text, we deleted the sentence and we reformulated it, so it can be better understood. We tried to say that after we conducted the Exploratory Factorial Analysis, we identified certain context in which students would prefer for platforms such as Facebook and Instagram to be used for educational purposes. Thus, we improved our explanation and we added the following text: “After conducting the factorial analysis, and after we analyzed the items corresponding to each factor of the scale (S2_EFA_Reliability_ Explained Variances), we identified certain contexts, circumstances related to the educational process in which students believe Facebook and Instagram can be used for educational purposes. These contexts are presented in the following sections.”

Line 413 became line 412 in the revised manuscript without the function “Track changes” active, and it became line 538 in the revised manuscript with the function “Track changes” and the option “All markup” active.

In the revised manuscript with the “Track changes” and “All markup” option active, the full change (the text we deleted and the text we inserted) can be viewed at lines 538-544.

**Reviewer 1 comment:**

Line 418 When referring to the didactic activity, to the formal educational process, students are of the opinion that teachers could use of Facebook for offering answers to their questions regarding the projects and tasks that they must accomplish, for inviting professionals in order to carry out debates on varied subjects, for sharing some of their experience related to their professional activity, for conducting surveys/polls on certain matters that concern the development of the courses and seminars, for informing students about any type of changes regarding the courses, or the deadlines for their projects.

Change to

Line 418 In consideration to the use of social media for formalized educational purposes, students believe teachers could use Facebook for providing answers to their questions concerning class projects and tasks, inviting professionals in their respective field for debates on varied subjects, sharing their experience related to professional activity, conducting surveys/polls on certain matters that concern the development of the courses and seminars, and, finally, informing students about any type of changes regarding the courses or the deadlines for their projects.

**Response:**

We changed the text according to the recommendation. Line 418 remained line418 in the revised manuscript without the function “Track changes” active, and it became line 547 in the revised manuscript with the function “Track changes” and the option “All markup” active.

In the revised manuscript with the “Track changes” and “All markup” option active, the full change (the text we deleted and the text we inserted) can be viewed at lines 547-559.

**Reviewer 1 comment:**

Line 446 With regards to the use of Facebook and Instagram by students during the didactic activity, students consider these social networks to be an appropriate place for them to manage a series of situations that require interaction and group collaboration.

Change to

Line 446 In consideration to the use of Facebook and Instagram by students as a didactic activity, students consider these social networks to be an appropriate place for them to manage interaction and group collaboration.

**Response:**

We changed the text according to the recommendation. Line 446 became line 443 in the revised manuscript without the function “Track changes” active, and it became line 579 in the revised manuscript with the function “Track changes” and the option “All markup” active.

In the revised manuscript with the “Track changes” and “All markup” option active, the full change (the text we deleted and the text we inserted) can be viewed at lines 579-583.

**Reviewer 1 comment:**

Line 454 With regards to the use of Facebook and Instagram by students in the context of developing extracurricular activities, students believe these platforms are suitable f

Change to

Line 454 In the context of using Facebook and Instagram for developing extracurricular activities, students believe these platforms are suitable…

**Response:**

We changed the text according to the recommendation. Line 454 became line 450 in the revised manuscript without the function “Track changes” active, and it became line 589 in the revised manuscript with the function “Track changes” and the option “All markup” active.

In the revised manuscript with the “Track changes” and “All markup” option active, the full change

(the text we deleted and the text we inserted) can be viewed at lines 589-592.

**Reviewer 1 comment:**

Line 463 Thus, considering the dimensions of extracurricular activates, our research revealed that students would like for Facebook and Instagram to be used by their teachers for conducting activities that are similar to the ones students would like to conduct on these platforms.

Change to

Line 463 Thus, considering the dimensions of extracurricular activities, our research revealed that students would like for Facebook and Instagram to be used by their teachers for conducting activities that are similar to the ones students would like to conduct on these platforms.

**Response:**

We changed the text according to the recommendation. Line 463 became line 458 in the revised manuscript without the function “Track changes” active, and it became line 598 in the revised manuscript with the function “Track changes” and the option “All markup” active.

In the revised manuscript with the “Track changes” and “All markup” option active, the full change

(the text we deleted and the text we inserted) can be viewed at lines 598-604.

**Reviewer 1 comment:**

Line 468 Previous studies on the matter of the use of social networks in higher education revealed that many universities use and integrate Facebook into the educational process

Change to

Line 468 Previous studies on social networks use in higher education revealed that many universities use and integrate Facebook into the educational process

**Response:**

We changed the text according to the recommendation. Line 468 became line 462 in the revised manuscript without the function “Track changes” active, and it became line 606 in the revised manuscript with the function “Track changes” and the option “All markup” active.

In the revised manuscript with the “Track changes” and “All markup” option active, the full change

(the text we deleted and the text we inserted) can be viewed at lines 606-609.

**Reviewer 1 comment:**

Line 475 Hence, if other countries opt for the integration and use of social networks in the educational act [25, 54, 55, 78], the use of such platforms in higher education institutions in Romania is rather modest, and often resumes to posting administrative information

Change to

Line 475 Hence, if other countries opt for the integration and use of social networks for educational purposes [25, 54, 55, 78], the use of such platforms in higher education institutions in Romania is rather modest, and often resumes to posting administrative information.

**Response:**

We changed the text according to the recommendation. Line 475 became line 468 in the revised manuscript without the function “Track changes” active, and it became line 611 in the revised manuscript with the function “Track changes” and the option “All markup” active.

In the revised manuscript with the “Track changes” and “All markup” option active, the full change

(the text we deleted and the text we inserted) can be viewed at lines 611-619.

**Reviewer 1 comment:**

Line 478 Thus, one of the reasons for conducting the present study is represented by the desire to carry out a research that could complement and come as a response to a similar study [56], which also focused on students’ motives for using Facebook

Change to

Line 478 Thus, one of the reasons for conducting the present study is represented by the desire to carry out a research that could complement and come as a response to a similar study [56], which also focused on students’ motives for using Facebook-this sentence is very unclear. I am not sure what you mean by this

**Response:** In order to clarify the sentence, we added an extra explanation. Thus, line 478 became line 470 in the revised manuscript without the function “Track changes” active, and it became line 619 in the revised manuscript with the function “Track changes” and the option “All markup” active.

In the text we explained that the research we conducted could complement, it could come as a response, to a previous study that approached the issue of the use of social networks for academic purposes in a similar manner. The previous study, [56], approached the issue from the view on students and it focused on the question of what should students post on their social networks accounts so as to maintain connection with their peers. However, we also approached the matter from the perspective of students but we focused on the question of what should teachers post on social network in order to better interact with students during the educational process.

In the document we added the following text: “In other words, our desire was to conduct a research that could come as a response to a previous study which also addressed the subject of the use of Facebook by students. The previous study [56] addressed the matter from the perspective of students, and focused on the question of what students should post on their Facebook accounts in order to maintain a proper interaction with their teachers. In this regard, our study also approaches the matter from the perspective of students, but it focuses on the question of what should teachers post on platforms such as Facebook and Instagram in order to interact with their students for academic purposes”

Thus, in the revised manuscript without the function “Track changes” active, the text can be seen at lines 473-480 and in the revised manuscript with the function “Track changes” and the option “All markup” active, the change (the text we inserted) can be seen at lines 622-629.

**Reviewer 1 comment:**

Line 481 Change to: In this regard, our research was conducted by taking into account the perspective of students regarding the ways they would like Facebook and Instagram to be used as educational tools in general, and in the context of the pandemic in particular.-The word “regard” is used redundantly. Consider alternative words

**Response:**

We changed the text according to the recommendation. We deleted the phrase “in this regard” and replaced it with “Hence”.

Line 481 became line 480 in the revised manuscript without the function “Track changes” active, and it became line 629 in the revised manuscript with the function “Track changes” and the option “All markup” active.

In the revised manuscript with the “Track changes” and “All markup” option active, the full change

(the text we deleted and the text we inserted) can be viewed at line 629.

**Reviewer 1 comment:**

Line 484 The study previously mentioned [56], conducted in Romania in 2011, showed that 70% of the respondents were of the opinion that Facebook was a low-cost tool that could be used to promote knowledge in higher education, but only 26,7% of them considered Facebook an appropriate instrument of change in higher education, an intrument through which changes in strategies, and attitudes during the educational process could be achieved.

Change to

Line 484 In a previously mentioned study [56], conducted in Romania in 2011, showed that 70% of the respondents were of the opinion that Facebook was a low-cost tool that could be used to promote knowledge in higher education, but only 26,7% of them considered Facebook an appropriate instrument of change in higher education, an instrument through which changes in strategies, and attitudes during the educational process could be achieved.

**Response:**

We changed the text according to the recommendation, but in order for the phrase to be better understood, before the word “showed” we also added “it”. Thus, the text was changed to:

“In a previously mentioned study [56], conducted in Romania in 2011, it showed that 70% of the respondents were of the opinion that Facebook was a low-cost tool that could be used to promote knowledge in higher education, but only 26,7% of them considered Facebook an appropriate instrument of change in higher education, an instrument through which changes in strategies, and attitudes during the educational process could be achieved.”

Line 484 became line 517 in the revised manuscript without the function “Track changes” active, and it became line 666 in the revised manuscript with the function “Track changes” and the option “All markup” active.

In the revised manuscript with the “Track changes” and “All markup” option active, the full change

(the text we deleted and the text we inserted) can be viewed at lines 666-675.

**Reviewer 1 comment:**

Line 489 However, a change for the better can be observed in the case of using Facebook for academic purposes.

Change to

Line 489 However, we believe Facebook can be more effectively used for academic purposes

**Response:**

We changed the text according to the recommendation. Line 489 became line 521 in the revised manuscript without the function “Track changes” active, and it became line 675 in the revised manuscript with the function “Track changes” and the option “All markup” active.

In the revised manuscript with the “Track changes” and “All markup” option active, the full change

(the text we deleted and the text we inserted) can be viewed at lines 675-677.

**Reviewer 1 comment:**

Line 506 help the socialize.

Change to

Line 506 help them socialize.

**Response:**

We changed the text according to the recommendation. Line 506 became line 538 in the revised manuscript without the function “Track changes” active, and it became line 694 in the revised manuscript with the function “Track changes” and the option “All markup” active.

In the revised manuscript with the “Track changes” and “All markup” option active, the full change

(the text we deleted and the text we inserted) can be viewed at line 694.

**Reviewer 1 comment:**

Line 507 Change to: tend to have a more positive attitude towards using Facebook as an educational tool, than Bachelor students.-This is nearly identical to a previously written sentence. Consider rewording, otherwise redundant.

**Response:**

We reworded the sentence in the following way: “are more willing to use Facebook for educational purposes than, Bachelor students.” Line 507 became line 540 in the revised manuscript without the function “Track changes” active, and it became line 694 in the revised manuscript with the function “Track changes” and the option “All markup” active.

In the revised manuscript with the “Track changes” and “All markup” option active, the full change (the text we deleted and the text we inserted) can be viewed at lines 694-698.

**Reviewer 1 comment:**

Line 510 With respect to the use of Instagram as an educational tool, students have a more reluctant attitude towards this social network

Change to

Line 510 With respect to the use of Instagram as an educational tool, expressed reservations towards this social network platform, considering its usefulness in the educational process occasionally, o

**Response:**

We changed the text according to the recommendation, but before the word “expressed”, we also added the word “students”, so that the phrase can be understood better. The text was changed to:

“With respect to the use of Instagram as an educational tool, students expressed reservations towards this social network platform, considering its usefulness in the educational process occasionally”

Line 510 became line 543 in the revised manuscript without the function “Track changes” active, and it became line 701 in the revised manuscript with the function “Track changes” and the option “All markup” active.

In the revised manuscript with the “Track changes” and “All markup” option active, the full change

(the text we deleted and the text we inserted) can be viewed at lines 701-705.

**Reviewer 1 comment:**

Line 514 From this result it can be inferred that, as found in other studies [77], Instagram too can be considered a complementary tool for the educational process

Change to

Line 514 Based on this inference, we found, like previous studies related to Instagram use for educational purposes, …

**Response:**

We changed the text according to the recommendation. Line 514 became line 545 in the revised manuscript without the function “Track changes” active, and it became line 706 in the revised manuscript with the function “Track changes” and the option “All markup” active.

In the revised manuscript with the “Track changes” and “All markup” option active, the full change

(the text we deleted and the text we inserted) can be viewed at lines 706-708.

**Reviewer 1 comment:**

Line 515 Change to: In the case of Instagram, students’ attitudes do not differ depending on their level of degree. In the case of both social networks, Facebook and Instagram, students who access daily their accounts consider to a greater extent that these platforms are suitable to be used for educational purposes.-This is redundant. You need to come up with alternative connective sentences. These are used identically in two consecutive sentences.

**Response:**

Thank you for the suggestion. We reformulated the sentences, in the following way:

“In the case of Instagram, it was found that students’ study degree does not have an influence on their attitude towards using this platform in the educational process. Even more, students who use daily their Facebook and Instagram accounts are of the opinion that these platforms could also be used for academic purposes.”

Line 515 became line 549 in the revised manuscript without the function “Track changes” active, and it became line 710 in the revised manuscript with the function “Track changes” and the option “All markup” active.

In the revised manuscript with the “Track changes” and “All markup” option active, the full change

(the text we deleted and the text we inserted) can be viewed at lines 710-716.

**Reviewer 1 comment:**

Line 519 Thus, students may have this attitude due to the fact that, by using these 26 social networks daily, by being more familiarized with their functions, they understand better the potential of these platforms to be used for academic purposes

Change to

Line 519 Students’ attitudes towards the use of social networks for educational purposes relates to their usage and familiarity with their programs.

**Response:**

We changed the text according to the recommendation. Line 519 became line 552 in the revised manuscript without the function “Track changes” active, and it became line 716 in the revised manuscript with the function “Track changes” and the option “All markup” active.

In the revised manuscript with the “Track changes” and “All markup” option active, the full change

(the text we deleted and the text we inserted) can be viewed at lines 716-720.

We thank you again for taking time to revise our manuscript and we also thank you for the very useful suggestions that you provided!

**Our response to Reviewer 2:**

We are very grateful for the time the reviewer spent on reviewing our article and for providing recommendations, for requesting us to discuss in the paper another important matter, that of social networks addiction. We sincerely appreciate the very useful suggestions made by the reviewer, and we tried our best to address each of the concerns and suggestions of the reviewer. Before we describe all the changes we made in order to comply with the requests of the reviewer, we mention that we made the changes while having active the “Track changes” function, with the “All markup” option. In this regard, the lines we will refer to when describing the changes, correspond to the lines that can be found in the revised manuscript where the “Track changes”, and “All markup” options are activated.

**Reviewer 2, comment 1:**

1) I understood that you focus the two most important social networking/media platforms, but why these two in particular? Why not using other platforms, such as YouTube, TikTok, Steemit, Researchgate, or Linkedin? Why not considering other platforms specifically thought for learning such as Skillshare and Coursera? In short, the paper should clearly state what Facebook and Instagram have to support the learning process that other platforms don't have.

**Response:** We thank you for recommending us to highlight the reasons why we only focused on Facebook and Instagram and not on other platforms. We found this suggestion very useful and we tried to provide a complete answer to all the questions presented in the first comment.

Thus, we firstly provided a short explanation for choosing to focus only on Facebook and Instagram, highlighting the fact that these platforms have impressive numbers of users. Then, for each of the platforms mentioned in comment 1: YouTube, TikTok, Steemit, Researchgate, or LinkedIN, Skillshare, Coursera, we provided a description of the reasons why they were not included in our research. Next, we described the elements, functions, that, in our opinion, Facebook and Instagram have in order to support online learning and that other platforms do not have. In other words, we emphasized the elements that determined us to focus our research only on Facebook and Instagram.

We inserted the text with the explanations in the Discussion section of the article, and the full change, can be viewed at lines 631-665 in the revised manuscript with the “All track” function and the “All markup” option active. In the unmarked version of the revised manuscript which does not have the “Track changes” active, the explanations we added can be found at lines: 482-516. Thus, we will further provide the exact lines that contain our responses for each of the questions that are highlighted in comment 1. We provide the lines for both the revised manuscript with “Track changes” and “All markup” option active, but also for the revised manuscript without the “Track changes” option activated.

Lines 631-634, in the manuscript with “Track changes” and “All markup” active, which are lines 482-484- in the revised manuscript which does not have active the function “Track changes”, and which represents the unmarked versions of our revised paper, describe shortly the reason for choosing to focus on Facebook and Instagram, and respond to the following question of the reviewer: “I understood that you focus the two most important social networking/media platforms, but why these two in particular? “

In this regard, at the lines mentioned above, our changes should be better seen while having active the “All Markup” and “Track changes” function. Thus, in the document with these options active, at lines 631- 634, we inserted into the paper the following explanation:

“These two platforms manage to register impressive numbers of users worldwide [10], and they are the subject of our research because they provide users with a wide range of functions and options.”

At lines 634-655 in the revised manuscript with “Track changes” and “All markup” active, which are lines 485-506- in the revised manuscript which does not have active the function “Track changes, we inserted explanations for not choosing to include in our research, the platforms: YouTube, TikTok, Steemit, Researchgate, or LinkedIN, Skillshare, Coursera, and thus we answered two of the questions highlighted in comment 1: “Why not using other platforms, such as YouTube, TikTok, Steemit, Researchgate, or Linkedin? Why not considering other platforms specifically tought for learning such as Skillshare and Coursera?”

Yotutube: at lines 636-640 – in the revised manuscript with “Track changes” and “All markup” ,which are lines 487-491- in the unmarked version of the manuscript, the one that does not use the “Track changes” function, we explained why we did not integrate YouTube into our research, stating that, even though on YouTube, users can post or access diversified content, we consider this platform less interactive than Facebook and Instagram, one of its disadvantages being that it does not allow users to create groups and interact in this way. The change can be best seen in the revised manuscript with “Track changes” and “All markup” active. Thus, at lines 636-640 in the paper with these options active, we inserted the following explanation:

“Hence, YouTube was not included in our research because, even though, through the functions and instruments that it incorporates, it can provide users with diversified content, we considered that this platform is less interactive because it does not allow the creation of groups, and thus it could hinder the process of real time communication between students.”

Tik Tok: at lines 640-647 in the revised manuscript with “Track changes” and “All markup” ,which are lines 491-498 - in the unmarked version of the manuscript, the one that does not use the “Track changes” function, we explained why we did not focus our research on Tik Tok. Thus, we emphasized the idea that Tik Tok is a relatively new social network, that it is used uneven by people and countries over the world, and this would have affected the replicability of our research. Even more, we also emphasized that, because people are usually excited about new things, by being a relatively new platform, answers of the responds might have been influenced by the novelty character of the platform. The text we inserted can be best viewed in the revised manuscript with “Track changes” and “All markup”, where at lines 640-647, we inserted the following explanation:

“As far as Tik Tok is concerned, being a relatively new platform, we considered that the analysis of the way it could be integrated into the educational process, could have altered the replicability of the research, because the development and use of Tik Tok globally is rather uneven. Even more, we believe that compared to Instagram and Facebook, Tik Tok has this novelty character that can lead to measurement errors. This errors might appear because people are generally excited about everything that is new in matter of technology, and thus their answers might have been influenced by the novelty of the platform Tik Tok.”

Steemit and Skillshare: At lines at lines 647-648 in the revised manuscript with “Track changes” and “All markup” ,which are lines 498-499 - in the unmarked version of the manuscript, the one that does not use the “Track changes” function, we explained that these platforms were not included in the research because they are not very popular in Romania, and they are used to a very little extent by people in Romania. The explanation can be best viewed at lines 647-648 in the revised manuscript with “Track changes” and “All markup” active. Thus the text that we inserted is the following:

“Next, as for Steemit and Skillshare, they were not included in our research because they are used to a very little extent in Romania.”

Research gate: at lines 648-650 in the revised manuscript with “Track changes” and “All markup” ,which are lines 499-501- in the unmarked version of the manuscript, the one that does not use the “Track changes” function, we provided an explanation for not focusing on Researchgate. We emphasized that this platform is mainly built for spreading knowledge by offering authors the chance to share their research, and by offering users access to their work. However our focus was on platform with more diverse options. The explanation can be best viewed at lines 648-650 in the revised manuscript with “Track changes” and “All markup” active. Thus the following text was inserted:

“Researchgate is a platform that aims to promote research, to spread knowledge through scientific papers, and we wanted to focus on social networks that offer more diverse functions.”

LinkedIn: at lines 650- 653 in the revised manuscript with “Track changes” and “All markup” ,which are lines 501-504- in the unmarked version of the manuscript, the one that does not use the “Track changes” function, we explained why we did not focus on LinkedIn. We argued that the platform is a more formal, business platform, which can be used by students for finding jobs or internships, and because we focused on social networks with varied functions, which are used for multiple purposes, this platform was not included in the research. The explanation can be best viewed at lines 650- 653 in the revised manuscript with “Track changes” and “All markup” active. Thus the following text was inserted:

“LinkedIn is a more formal, business platform, that could be used by students and teachers for searching for jobs and internships, but our interest was in finding out how social networks more general and comprehensive, like Facebook, could be integrated into the educational process.”

Coursera: at lines 653-655 in the revised manuscript with “Track changes” and “All markup” ,which are lines 504-506- in the unmarked version of the manuscript, the one that does not use the “Track changes” function, we stated why we did not focus on Coursera. We highlighted the fact that in the paper we wanted to focus on platforms that were not initially designed for learning, and in this regard, Coursera could not be included in our research. The change we made to the text can best seen at lines 653-655 in the revised manuscript with “Track changes” and “All markup” active. Thus at this lines, the following text was inserted:

“Furthermore, Coursera was not in our area of interest because the focus of this paper was on social networks, on platforms that were not initially designed for educational purposes.”

At lines 656-665 in the revised manuscript with “Track changes” and “All markup” ,which are lines 507-516- in the unmarked version of the manuscript, the one that does not use the “Track changes” function, we described the elements that Facebook and Instagram have, elements that in our opinion make them more suitable for supporting the learning process. In this regard, we addressed the last recommendation that is written in comment 1.

Thus, we emphasized the variety of functions that the platforms offer, the fact that they can help students collaborate better, and that students are already familiar with them, and that it might be beneficial for them to learn in an environment they already appreciate. The change we made to the text can best seen at lines 656-665 in the revised manuscript with “Track changes” and “All markup” active. Thus at this lines, the following text was inserted:

“Taking into account the aforementioned aspects, we argue that, compared to other social networks, Facebook and Instagram can better support the learning process. These platforms incorporate a wide variety of functions that allow the creation of diversified content, they allow students to properly communicate and collaborate with each other during courses ( for example, on Facebook, teachers could create groups and students could work in teams in order to fulfil certain tasks, that can later be presented to the other teams on a live meeting), and last but not least, students are familiar with these social networks, and even though they usually use them to relax and communicate with their friends, because they are already accustomed to them, students may be more relaxed, more creative and more willing to learn in a familiar environment.”

**Reviewer 2, comment 2:**

2) In the Introduction the very first paragraph is not convincing (lines 50-60). In particular:

- Reference 3 states that for 70% of the students Facebook is not superior to Moodle, 90% of the students find Facebook not appropriate for learning (see the paper's table 2)

- Reference 6 discusses how social networking sites can be used in the learning process, but does not prove the usefulness.

I think the authors should replace these references with other more relevant

**Response:**

We thank you very much for this recommendation. We tried our best in order to comply with it, and we replaced both Reference 3 and Reference 6.

Reference 3 was replaced with another source that emphasized that social networks like Facebook, have an important role in the life of university students. The full change (the text deleted and the text inserted) is visible, at lines 839-843 in the revised manuscript with “Track changes” and “All markup” active. In the unmarked version of the manuscript, the one without Tracked changes, the new Reference 3 can be found at lines 672-673.

Thus, in the References section, in the in the revised manuscript with “Track changes” and “All markup” active, at lines 839-843 we deleted Reference 3 and replaced it with a new reference:

The old Reference 3: Anderson GS, Dogoriti E, Pange J. The use of social networking and learning management systems in English language teaching in higher education. Campus-Wide Information Systems, 2014; 31(4):254-263. doi: 10.1108/CWIS-11-2013-0062

The new Reference 3: Duncan D, Barczyk C. Facebooks Effect on Learning in Higher Education: An Empirical Investigation. Information Systems Education Journal, 2016; 14(3): 14-28

In the Introduction section, we inserted explanations according to the new Reference 3. Thus, at lines 55-56, in the in the revised manuscript with “Track changes” and “All markup” active, the text “Studies show that social networks play an important role in the life of university students, their focus being on collaboration and communication” which belonged to the old Reference 3 was deleted, and we inserted the following information, that can be found in the new Reference 3:

“Studies reveal that social networks are important in the life of university students, platforms such as Facebook being considered by students appropriate for supplementing the traditional face- to – face courses, and also being perceived as platforms that can improve the quality of the courses and which can help them better communicate with their peers and feel more connected to them.” [3].

The full change (the text deleted and the text inserted) can be viewed at lines 55-60 in the in the revised manuscript with “Track changes” and “All markup” option active. Only the explanation that we inserted according to the new Reference 3, and not the full change, can be seen in the unmarked version of the manuscript, the one without Tracked changes, at lines 55-59.

Reference 6 was replaced with a reference which shows that social networks are efficient and useful in the process of learning in higher education. Thus, we believe the new reference is more suitable for supporting the statement that the use of social networks was proven to be effective in many universities, because in the new Reference 6, the author shows that the use of Facebook for educational purposes, while delivering an online course enhanced the process of learning. Students’ engagement increased, many discussions were initiated voluntary by them, without being requested to do so. Even more, the study also shows that the use of Facebook was effective in the educational process because it allowed teachers to offer students access to diverse course materials, such as video tutorials.

The full change (the text deleted and the text inserted) is visible, at lines 850- 854 in the revised manuscript with “Track changes” and “All markup” active. In the unmarked version of the manuscript, the one without Tracked changes, the new Reference 6 can be found at lines 680-682.

The old Reference 6: Faizi R, El Afia A, Chiheb R. Exploring the potential benefits of using social media in education. International Journal of Engineering Pedagogy (iJEP). 2013; 3(4): 50-53. doi:10.3991/ijep.v3i4.2836

The new Reference 6: Esteves KK. Exploring Facebook to Enhance Learning and Student Engagement: A Case from the University of Philippines (UP) Open University. Malaysian Journal of Distance Education. 2012; 14(1): 1-15.

**Reviewer 2, comment 3:**

3) Platforms such as Facebook and Instagram were shown to generate pethological addiction (see <https://www.springerprofessional.de/en/discovering-the-impact-of-notifications-on-social-network-addict/18929896>) also in the academic scenario (see <https://www.sciencedirect.com/science/article/pii/S0736585314000021>). How do you frame this problem in the usage of social networks for learning? Since this problem is mainly due to the "just one more post/picture/video" effect, where you keep consuming contents mindlessly for hours, what is the impact of using such an unrestricted platform for learning? Does Moodle and other such platforms not provide better support? How much of the interviewers were in favour of using, say, Facebook just because they can have access to it without feeling guilty of not studying? I think the pper should discuss this problem.

**Response:**

We are very grateful for suggesting us to discuss in the paper the issue of social networks addiction. We took the suggestion very seriously, and since we also believe that social networks addiction is a serious problem nowadays, we tried to comply with the suggestion, and to provide answers to the questions highlighted in comment 3 in the best way possible.

In the Discussion section of the text, the full explanation, (the text we inserted) can be found at lines 721-770 in the revised manuscript with “Track changes” and “All markup” active. In the unmarked version of the manuscript, the one without Tracked changes, the text we inserted can be found at lines 555-604.

The two references pointed out in comment 3 were very useful and we are very thankful for providing them to use. We took into account the references when we provided answers to the questions highlighted in comment 3. Thus, we cited the references in the text, and we also added them to the Reference section.

The first reference suggested has the number [84] in the text: Nasti L, Michienzi A, Guidi B. Discovering the Impact of Notifications on Social Network Addiction. In Bowels J, Broccia G, Nanni M, editors. From Data to Models and Back: 9th International Symposium, DataMod 2020, Virtual Event, October 20, 2020, Revised Selected Papers 9, Springer, 2021; pp. 72-86

Reference [84] 1084- 1087 can be found at lines in the revised manuscript with “Track changes” and “All markup”, which are lines 912-915- in the unmarked version of the manuscript, the one that does not use the “Track changes” function.

The second reference suggested has the number [85] in the text: Hong FY, Huang DH, Lin HY, Chiu SL. Analysis of the psychological traits, Facebook usage, and Facebook addiction model of Taiwanese university students. Telematics and Informatics. 2014; 31(4): 597-606.

Reference [85] can be found at lines 1088- 1090 in the revised manuscript with “Track changes” and “All markup”, which are lines 916-918- in the unmarked version of the manuscript, the one that does not use the “Track changes” function.

At lines 721- 749, in the revised manuscript with “Track changes” and “All markup” ,which are lines 555-583- in the unmarked version of the manuscript, the one that does not use the “Track changes” function, we offered explanations and answers for the first and second question highlighted in comment 1: “How do you frame this problem in the usage of social networks for learning? Since this problem is mainly due to the "just one more post/picture/video" effect, where you keep consuming contents mindlessly for hours, what is the impact of using such an unrestricted platform for learning?” We firstly expressed or view on the matter of social network addiction in the academic field and in the learning process. We emphasized the fact that students spend a lot of time on social networks and they use them for many purposes, but using them for academic and educational purposes should not worsen issue of social networks addiction. In this regard, we stated that because of the unrestricted character of platforms such as Facebook and Instagram, teachers could provide students different types of content, and during courses held with the help of these platforms teachers could also help students prevent or overcome the social network addiction, they could offer them counseling. Even more, we also stated that teachers should start looking for ways to encourage students to use social networks in a more beneficial way.

Hence, at lines 721- 749, in the revised manuscript with “Track changes” and “All markup” ,which are lines 555-583- in the unmarked version of the manuscript, the one that does not use the “Track changes” function, the following text was inserted:

“Furthermore, when addressing the subject of the use of social networks, regardless of the purpose for which they are being used, a discussion about students’ addiction to platforms such as Facebook and Instagram is relevant and necessary. Thus, a previous study which took into account the mathematical model of people’s Dopamine System model, analysed the behavior of Facebook users belonging to 18 Facebook groups from domains such as education, sport, entertainment, work or news, and revealed that the notifications people receive on social networks can generate addiction [84]. With respect to the academic field, previous studies also showed that university students can become addicted to Facebook, according to some of their personality and psychological traits such as depressive character, and that the more time students spend of Facebook, the more likely are to develop Facebook addiction [85].

Social network addiction is a serious problem, and while taking into account the findings of previous studies, we argue that, by using social networks in the teaching-learning process, teachers could actually help students overcome this problem. Hence, it is known that students spend many hours on platforms like Facebook, study [85] showing that the majority of the respondents daily used Facebook for more than 4,5 hours. Thus, students consume content posted on social network for many hours and can slowly become addicted by saying to themselves that they will only watch one more post. In this regard, students use social networks for varied purposes, but we are of the opinion that the use of these platforms for educational purposes should not increase students’ level of addiction. On the contrary, because they are platforms where the content is not restricted, or it is restricted to a very little extent, teachers can share diversified course related content, but also other type of information that could help students understand the problem of addiction, that could help them cope with it and teachers may encourage students to use social networks like Facebook and Instagram in order to expand their knowledge, to share ideas, and not just use them for entertainment purposes. In other words, universities and teachers should focus more on findings ways through which students could be encouraged to use social networks in more positive and beneficial ways, and thus, by making them use Facebook and Instagram for academic purposes, professors could also teach their student about addiction, they could offer them advices and counselling.”

Next, we tried to address the third and the fourth question highlighted in comment 3: “Does Moodle and other such platforms not provide better support? How much of the interviewers were in favour of using, say, Facebook just because they can have access to it without feeling guilty of not studying?”

We are very thankful for raising such important issues and for suggesting us to discuss them in our paper. We tried to comply with the suggestions and we further describe how we responded to the third questions as well as how we responded to the fourth question.

At lines 750-757 in the revised manuscript with “Track changes” and “All markup”, which are lines 584-591- in the unmarked version of the manuscript, the one that does not use the “Track changes” function, we provided an explanation for the third question highlighted in comment 3. In this regard, we mentioned in our explanation that we are of the opinion that Moodle platforms do provide adequate support for conducting the educational process, and we emphasized the fact that by encouraging the use of Facebook and Instagram as educational tools, we do not believe that Moodle platforms should no longer be used. Thus, the aim is to use social network like Facebook and Instagram as complementary tools for Moodle platforms in order to enhance the teaching-learning process.

Thus, at lines 750-757, in the revised manuscript with “Track changes” and “All markup” ,which are lines 584-591- in the unmarked version of the manuscript, the one that does not use the “Track changes” function, we inserted the following text:

“Taking into consideration the aspects mentioned above, certain issues can be addressed. The first issue refers to whether Moodle platforms provide better support than social networks for carrying out the educational process. Hence, we argue that, Moodle platforms are suitable for conducting the educational process and the aim is not to eliminate the use of Moodle platforms for delivering courses, but to add value to the educational process by supplementing it with the use of social networks. Because of their nature, and because students are familiar with these platforms and enjoy using them, social networks could enhance the learning process, and in this regard, they could be used as complementary tools.”

At lines 758- 770 in the revised manuscript with “Track changes” and “All markup”, which are lines 592-604- in the unmarked version of the manuscript, the one that does not use the “Track changes” function, we provided an explanation for the fourth question highlighted in comment 4. Hence, we emphasized that by integrating social networks into the educational process, students could learn how to use this platforms in a way that is more beneficial to them. Even more, we also stated that, the fact that students will accept to use social networks for academic purposes does not necessarily mean that they will prefer to use these platforms because in this way they could have a justified reason for accessing them without feeling guilty for not studying. In other words, the act of using Facebook and Instagram for academic purposes, should not be seen as a method through which students could eliminate their guilt for not studying, because the act of using these platforms in order to expand their knowledge, can already be seen as a step that students made towards studying.

At lines 758- 770 in the revised manuscript with “Track changes” and “All markup”, which are lines 592-604- in the unmarked version of the manuscript, the one that does not use the “Track changes” function“, the following text was inserted:

“Another issue that arises when approaching the subject of using Facebook and Instagram as educational tools, refers to the matter of whether students will be willing to use these platforms only because they would have a reasonable motive for using them, thus not developing a feeling of guilt for not studying. Thus, as we previously mentioned, by integrating social networks in the educational process, we believe that students could be taught how to make better use of them. In this regard, the act of using social networks for academic purposes should not be seen as a method that helps students eliminate their guilt for not studying, because the activities that can be carried out through these platforms are activities meant to help students study. In other words, being encouraged and accepting to use social networks for educational purposes, does not mean that students will prefer to use such platforms because in this way they can eliminate the guilt for not studying, because the action of using Facebook and Instagram for academic purposes itself is an action that the student made in order to learn and acquire knowledge.

We want to thank again the reviewer for taking the time to review our paper and for providing us very useful suggestions!

Further, we describe how we responded to the points raised by the academic editor in order to meet the requirements of the journal.

**Journal requirements:**

When submitting your revision, we need you to address these additional requirements.

**Journal requirement 1**:

1. Please ensure that your manuscript meets PLOS ONE's style requirements, including those for file naming. The PLOS ONE style templates can be found at

<https://journals.plos.org/plosone/s/file?id=wjVg/PLOSOne_formatting_sample_main_body.pdf> and

<https://journals.plos.org/plosone/s/file?id=ba62/PLOSOne_formatting_sample_title_authors_affiliations.pdf>

**Response:**

We thank you for suggesting us to check again if our manuscript meets the journal’s style requirements. In order to comply with the requirements we checked if our manuscript is formatted according to the guidelines provided by the journal. After we consulted the documents, we made some changes to the text, while having active the “Track changes” function and the option “All markup”. In the title page we improved the Affiliations of the authors by including the departments in which they work and the name of the city. Also, we deleted the street address that we provided in the initial manuscript and we added the initials of the corresponding author.

Next, we describe point by point each changes that we made, and we also provide the lines where the changes can be found.

Line 3 in the revised manuscript with “Track changes” and “All markup” active and in the unmarked version of the manuscript, the one that does not use the “Track changes” function.

At this line, the number assigned to the fourth author (Gabriela Grosseck) was changed from “2” to “3”. Initially, the third and fourth author of the paper were assigned the number 2. Then, when we noticed that we also have to mention the departments in which they work, we changed the number because, even though the author (Gabriela Grosseck) works in the same university as the third author of the paper (Laurentiu Gabriel Tiru), they work in different departments.

Line 5 in the revised manuscript with “Track changes” and “All markup” active and in the unmarked version of the manuscript, the one that does not use the “Track changes” function. At this line we added the department of the first, second, and fifth author of the paper. Thus, the following information was added: “Department of Social Sciences and Communication.”

Line 6 in the revised manuscript with “Track changes” and “All markup” active and in the unmarked version of the manuscript, the one that does not use the “Track changes” function“. At this line we added the name of the city in which the university of the first, second and fifth author of the paper is located. The following city was added: “Brasov”.

Line 7 in the revised manuscript with “Track changes” and “All markup” active and in the unmarked version of the manuscript, the one that does not use the “Track changes” function. At this line we added the department of the third author of the paper. Thus, the following information was added: “Department of Sociology”

Line 8 in the revised manuscript with “Track changes” and “All markup” active and in the unmarked version of the manuscript, the one that does not use the “Track changes” function. At this line we added the name of the city in which the university of the third author of the paper is located. The following city was added: “Timisoara”.

Lines 9-10 in the revised manuscript with “Track changes” and “All markup” active and in the unmarked version of the manuscript, the one that does not use the “Track changes” function. At this lines we provided the affiliation for the fourth author of the paper. The third and fourth author of the paper work in the same university but in different departments, and thus their affiliations are separated. In this regard, at the lines mentioned above we inserted the following: “Department of Psychology, Faculty of Sociology and Psychology, West University of Timisoara, Timisoara, Romania;”

Line 11 in the revised manuscript with “Track changes” and “All markup” active and in the unmarked version of the manuscript, the one that does not use the “Track changes” function. At this lines we added the department to the address we provided. We inserted the following text “Department of Social Sciences and Communication”

Line 12 in the revised manuscript with “Track changes” and “All markup” active and in the unmarked version of the manuscript, the one that does not use the “Track changes” function. At this line we deleted the street address that we mentioned in the initial manuscript and we added the name of the city to the address provided. Thus, we inserted the following city: “Brasov”

Line 15 in the revised manuscript with “Track changes” and “All markup” active and in the unmarked version of the manuscript, the one that does not use the “Track changes” function. At this line we added the initials of the corresponding author. Thus we added: “CC”

Next, in order to meet the requirements of the journal, we changed the dimensions of the font for the article’s headings. Thus, each level 1 heading now has an 18 point font, each level 2 heading has a 16 point font, and each level 3 heading has a 14 point font.

Level 1 headings:

Abstract - the change we made can be seen at line 25 in the revised manuscript with “Track changes” and “All markup” options active.

Introduction - the change we made can be seen at line 47 in the revised manuscript with “Track changes” and “All markup” options active.

Clarifying the concept of social networks- - the change we made can be seen at line 107 in the revised manuscript with “Track changes” and “All markup” options active.

Social networks as tools in educational process- the change we made can be seen at line 141 in the revised manuscript with “Track changes” and “All markup” options active.

Facebook and Instagram as educational tools - the change we made can be seen at line 210 in the revised manuscript with “Track changes” and “All markup” options active.

Materials and methods- the change we made can be seen at line 339 in the revised manuscript with “Track changes” and “All markup” options active.

Results- the change we made can be seen at line 472 in the revised manuscript with “Track changes” and “All markup” options active.

Discussion- the change we made can be seen at line 605 in the revised manuscript with “Track changes” and “All markup” options active.

Conlusions- the change we made can be seen at line 771 in the revised manuscript with “Track changes” and “All markup” options active.

Acknowledgements - the change we made can be seen at line 814 in the revised manuscript with “Track changes” and “All markup” options active.

Author Contributions- the change we made can be seen at line 818 in the revised manuscript with “Track changes” and “All markup” options active.

References- the change we made can be seen at line 831 in the revised manuscript with “Track changes” and “All markup” options active.

Supporting information- the change we made can be seen at line 1092 in the revised manuscript with “Track changes” and “All markup” options active.

Level 2 headings:

Sampling and data collection procedures- the change we made can be seen at line 340 in the revised manuscript with “Track changes” and “All markup” options active.

The Research Instrument- the change we made can be seen at line 363 in the revised manuscript with “Track changes” and “All markup” options active.

Statistical analyses- the change we made can be seen at line 399 in the revised manuscript with “Track changes” and “All markup” options active.

What is the attitude of students towards the use of Facebook and Instagram in the educational process?- the change we made can be seen at lines 473-474 in the revised manuscript with “Track changes” and “All markup” options active.

What are the contexts that students consider appropriate for the use of Facebook and Instagram in the educational process?- the change we made can be seen at lines 535-536 in the revised manuscript with “Track changes” and “All markup” options active.

Limitations and future research directions - the change we made can be seen at line 804 in the revised manuscript with “Track changes” and “All markup” options active.

Level 3 headings:

The use of Facebook and Instagram by teachers- the change we made can be seen at line 546 in the revised manuscript with “Track changes” and “All markup” options active.

The use of Facebook and Instagram by students- the change we made can be seen at line 578 in the revised manuscript with “Track changes” and “All markup” options active.

**Journal requirement 2**:

2. Please include additional information regarding the survey or questionnaire used in the study and ensure that you have provided sufficient details that others could replicate the analyses. For instance, if you developed a questionnaire as part of this study and it is not under a copyright more restrictive than CC-BY, please include a copy, in both the original language and English, as Supporting Information.

**Response:**

We developed a questionnaire in order to conduct our research. In order to meet the second requirement we included as Supporting Information, a copy of the questionnaire, in English (S4 questionnaire English version) and a copy in the original language – Romanian (S5 questionnaire Romanian version).

With the initial manuscript we uploaded as Supporting Information a document (S1 questionnaire), that actually contained the initial versions of the scales that we developed. The document includes the dimensions, the items and the questions of the scales. In this regard we want to highlight two changes that we made:

- Because Reviewer 1 asked us to provide an explanation regarding why some of the items of the scales were removed, we provided the explanation, but we also added to the document (S1 questionnaire- which was now given the name “S1 Initial versions of the scales” in order to avoid any confusions), some of the items that we removed, and that were not present in the document we initially submitted. These items are:

f20.p Facebook in an appropriate platform for teachers to maintain connection with students

f21.p Facebook is an appropriate platform for teachers to maintain connection with graduates

f23.p Facebook is an appropriate platform for teachers to communicate with students about certain personal problems that have an impact on their academic performance

f24.p Facebook is an appropriate platform for teachers to manage o series of situation that requires group interaction

f19.s Facebook is an appropriate platform for students to manage a series of situations that require interaction and group consensus

f21.s Facebook is an appropriate platform for students to post information about the projects they are involved in

i17p. Instagram is an appropriate platform for teachers to maintain connection with students

i18p. Instagram is an appropriate platform for teachers to maintain connection with graduates

i17s. Instagram is an appropriate platform for students to post information about the projects they are involved in

- Because you also requested us to provide the questionnaire that we developed, in the original language and in English, in order to avoid any confusions, the name of the document “S1 questionnaire”, was changed to “S1 Initial versions of the scales”.

Thus, to the three documents that we submitted with the initial manuscript, we added two more documents, entitled: “S4 questionnaire English version” and “S5 questionnaire Romanian version”.

**Journal requirement 3**

Thank you for stating the following in the Acknowledgments Section of your manuscript:

[We kindly thank the respondents for participating in the study and we thank Transilvania University of Brasov and West University of Timisoara for their support.]

We note that you have provided funding information that is not currently declared in your Funding Statement. However, funding information should not appear in the Acknowledgments section or other areas of your manuscript. We will only publish funding information present in the Funding Statement section of the online submission form.

Please remove any funding-related text from the manuscript and let us know how you would like to update your Funding Statement. Currently, your Funding Statement reads as follows:

[The authors received no specific funding for this work.]

Please include your amended statements within your cover letter; we will change the online submission form on your behalf.

**Response:**

Thank you for pointing out the issues regarding our Funding statement.

We want to state clearly that we did not receive any funding for our research.

The information written in the Acknowledgments Section of our manuscript was changed in order to make it clear that we did not receive any funding. In the Acknowledgments Section we simply want to thank the participants of our study for completing the questionnaire voluntarily, without receiving any incentives.

Thus, the information:

[We kindly thank the respondents for participating in the study and we thank Transilvania University of Brasov and West University of Timisoara for their support.]

Was changed to:

[We kindly thank the respondents for participating in the study, especially because they agreed to voluntary participate in our study, without receiving any incentives.]

The change can be seen in the revised manuscript with “Track changes” and “All markup” options active at lines 815- 817 and at lines 649-650 in the unmarked version of the manuscript, the one that does not use the “Track changes” function.

Therefore, since we did not receive any funding, our Funding statement should remain the same as we declared initially:

[The authors received no specific funding for this work.]

We appreciate the effort the academic editor and the reviewers made in order to provide suggestions that could improve our article, and we thank you for reviewing the article.

Sincerely,

Prof. Dr. Claudiu Coman
