# Supplementary material for: Dear student, what should I write on my wall? A case study on academic uses of Facebook and Instagram during the pandemic
Source: PLoS One. 2021 Sep 23;16(9):e0257729. doi: 10.1371/journal.pone.0257729 (PMC8459956; doi:10.1371/journal.pone.0257729)
Supplement: Attachment — Submitted filename: Response to reviewers.docx [file pone.0257729.s008.docx]

**Rebuttal letter**

Claudiu Coman

Transilvania University of Brasov

[claudiu.coman@unitbv.ro](mailto:claudiu.coman@unitbv.ro)

Dear Sir/Madam

With this cover letter we submit the revised manuscript entitled:” Dear student, what should I write on my wall? A case study on academic uses of Facebook and Instagram during the pandemic” Claudiu Coman, Luiza Mesesan-Schmitz, Laurentiu Gabriel Tiru, Gabriela Grosseck, and Maria Cristina Bularca for publication in PLOS ONE.

We revised the manuscript according to the suggestions made by the reviewers and we also addressed the points raised by the academic editor with regard to the aspects that we had to pay attention to in order to meet the requirements of the journal. We would like to thank the reviewers for taking time to review our paper and for providing such useful suggestions. We also thank the academic editor for pointing out the aspects that we should address in order to improve the paper and make sure that it meets the requirements of the journal. We tried to comply with all the suggestions and recommendations made by the reviewers and the academic editor.

In this letter, we describe the changes we made to the text according to the recommendations of the reviewers and of the academic editor. Firstly, we described all the changes we made in order to comply with the comments of the reviewers, and then we provide explanations to the issues raised by the academic editor.

Our manuscript needed minor revisions. The changes were made while having active the “Track changes” function from Microsoft Word and the lines where the text was changed can be best viewed while having active the “All markup” option. Moreover, in order for our changes to be best seen, we will provide the lines from the revised manuscript with the “Track changes” function, and “All markup” option active, as well as the lines from the version 1 of the revised manuscript without the “Track changes” function, and “All markup” option active, (the manuscript consulted by the reviewers), and the lines from the version of the manuscript without the “Track changes” function that we are submitting together with this cover letter.

Further, we provide a brief summary of the changes we made to the text depending on the comments of the reviewers. Reviewer 1 provided suggestions and recommendations regarding the way some parts of the text should be formatted, or regarding the way some words or punctuation marks should be changed in order to improve the article. We thank the reviewer for taking the time to provide such useful suggestions. We complied with all of the suggestions of Reviewer 1, and each change made according to the comments of Reviewer 1 will be described later in this letter. In regard to Reviewer 2 comments, we thank the reviewer for spending time to review our paper and to review the way we addressed his/hers previous comments. We are very grateful for the suggestion made by Reviewer 2 and we complied with it.

Next, we will describe point by point each of the changes we made to the text. Thus, we would like to mention that the changes can be best seen while having active the “Track changes” and “All markup” option from Microsoft Word.

**Our response to Reviewer 1:**

We firstly thank the reviewer for taking time to review our manuscript and provide suggestions in order to improve it. We addressed all the suggestions made by the reviewer and changed the text in the way the reviewer suggested. Hence, when we described how the text was changed, we provide firstly the lines where the text can be found in the revised manuscript with the option “Track changes” active. In this way, the changes can be viewed completely (the text we deleted, and the text we inserted). Next, we also provide the lines where the correct text, according to the recommendations of the reviewer, can be found in the manuscript without the “Track changes” option active, in the manuscript that we submit together with this cover letter.

**Reviewer 1 comment:**

Line 48

Minor formatting concern-should be another space between subtitle and text.

**Response:**

We thank you for the suggestion. We added another space between the subtitle and the text. The change can be seen at line 48 (where we added the space) in the revised manuscript with the option “Track changes” and “All markup” active, as well as in the manuscript without the “Track changes” option active.

**Reviewer 1 comment:**

Line 57 traditional face- to – face courses, and also being perceived as platforms that can improve the

Just a minor comment-it looks like a different symbol was used or the font varies in the face-to-face section.

**Response:**

We thank you for the suggestion. We firstly want to mention that, because of the space we added at line 48, the numbers of the lines as seen by the reviewer in the manuscript changed. Thus, line 57 is now line 58. The symbol used was indeed different. We corrected the text, and the expression “face- to – face courses” was replaced with “ face - to - face courses”. The change can be seen at line 59 in the revised manuscript with the option “Track changes” and “All Markup” active and at line 58 in the manuscript without the “Track changes” option active.

**Reviewer 1 comment:**

Line 95 between people [11] 96 Social networks have been defined by Boyd and Ellison, as web based services that offer

This paragraph seems too short. It like it, but you may want to incorporate it into its following paragraph.

**Response:**

We thank you for the useful suggestion. We incorporated the paragraph in the following paragraph. Line 95 became 96 in the revised manuscript without the “Track changes” option. Thus, the change can be seen at line 112 in the revised manuscript with the option “Track changes” and “All Markup” active, and at line 96 in the manuscript without the “Track changes” option active.

**Reviewer 1 comment:**

Line 133 showed that 42, 17% of the

I think this is typo. Maybe you mean to say showed that 17% of the …?

**Response:**

In reference 28, at page 209 the authors described the number of respondents which consider that the possibility of using social networks is considered a good possibility by providing the percent 42.17%, a percent with two decimals. We corrected the way we wrote the percent and we replaced the comma “,”, with a point. Thus, “42, 17%”, became “42.17%”. Line 133 remained line 133 in the revised manuscript without the “Track changes” option. The full change (the deleted comma and the point added) can be seen at line 149 in the revised manuscript with the option “Track changes” and “All Markup” active, and at line 133 in the manuscript without the “Track changes” option active.

**Reviewer 1 comment:**

Line 136 online communication, social networks helping them accommodate

Run on sentence, perhaps say …online communication, with social networks helping them accommodate easier to university life, and by…

**Response:**

We changed the text according to the recommendation. Line 136 remained line 136 in the manuscript without the “Track changes” option active. The full changed ( the text deleted and the text we inserted) can be seen at lines 152 - 154 in the revised manuscript with the option “Track changes” and “All Markup” active, and at lines 136 - 137 in the manuscript without the “Track changes” option active.

**Reviewer 1 comment:**

Line 163 collaboration between students, by allowing them to chat in real time that facilitates effective feedback and 164 comments on the posts of their peers

**Response:**

We made the changes to the text as you suggested. Line 163 remained line 163 in the manuscript without the “Track changes” option active. The full change can be seen at lines 192 -193 in the revised manuscript with the option “Track changes” and “All Markup” active, and at lines 136 - 137 in the manuscript without the “Track changes” option active.

**Reviewer 1 comment:**

Line 164 Social networks help teachers

**Response:**

We changed the text according to the recommendation. Thus, “Social networks, help teachers” became “Social networks help teachers”. Line 164 remained line 164 in the manuscript without the “Track changes” option active. The change can be seen at line 196 in the revised manuscript with the option “Track changes” and “All Markup” active, and at line 164 in the manuscript without the “Track changes” option active.

**Reviewer 1 comment:**

Line 170 platforms such as Facebook registered lower grade-point averages,

**Response:**

We changed the text according to the recommendation. Thus, “platforms such as Facebook, registering lower grade-point averages,” became “platforms such as Facebook registered lower grade-point averages”. Line 170 remained line 170 in the manuscript without the “Track changes” option active. The change can be seen at line 206 in the revised manuscript with the option “Track changes” and “All Markup” active, and at line 170 in the manuscript without the “Track changes” option active.

**Reviewer 1 comment:**

Line 177 internet use, in January 2020 there were 3.8 billion social media users and Facebook, having 178 2.44 billion

**Response:**

We changed the text according to the recommendation. Thus, “internet use, in January 2020 there were 3, 8 billion social media users and Facebook, having 2,44 billion monthly users” became “internet use, in January 2020 there were 3.8 billion social media users and Facebook, having 178 2.44 billion”. Line 177 remained line 177 in the manuscript without the “Track changes” option active. The change can be seen at line 220 in the revised manuscript with the option “Track changes” and “All Markup” active, and at lines 177- 178 in the manuscript without the “Track changes” option active.

**Reviewer 1 comment:**

Line 194 high interest for studying the effects of Facebook and what students use it for [55]. 195 According

Connect these two paragraphs

**Response:**

Thank you for the recommendation. We connected the paragraphs. Line 194 remained line 194 in the manuscript without the “Track changes” option active. The change can be seen at line 243 in the revised manuscript with the option “Track changes” and “All Markup” active, and at line 194 in the manuscript without the “Track changes” option active.

**Reviewer 1 comment:**

Line 201 students also use Facebook for discussing educational content, [57], while another research

Word research is becoming redundant. Perhaps say study for one instead.

**Response:**

Thank you for the recommendation. We replaced the word “research” with “study”. Line 201 remained line 201 in the manuscript without the “Track changes” option active. The change can be seen at line 253 in the revised manuscript with the option “Track changes” and “All Markup” active, and at line 201 in the manuscript without the “Track changes” option active.

**Reviewer 1 comment:**

Line 222 educational tool [66]. 11 223 Instagram is

Connect these two paragraphs

**Response:**

Thank you for the recommendation. We connected the paragraphs. Line 222 and line 223 remained line 222 and line 223 in the manuscript without the “Track changes” option active. The change can be seen at line 286 in the revised manuscript with the option “Track changes” and “All Markup” active, and at line 222 in the manuscript without the “Track changes” option active.

**Reviewer 1 comment:**

Line 254 the present cross

Minor formatting concern-should be another space between subtitle and text.

**Response:**

Thank you for the recommendation. We added a space between the subtitle and the text. Line 254 became line 253 in the manuscript without the “Track changes” option active, because of the previous changes made to the text. After adding the space, the line received the number 254 again. The change (the space added) can be seen at line 365 in the revised manuscript with the option “Track changes” and “All Markup” active, and at line 253 in the manuscript without the “Track changes” option active.

**Reviewer 1 comment:**

Line 272 Based on the data gleaned

Minor formatting concern-should be another space between subtitle and text.

**Response:**

Thank you for the recommendation. We added a space between the subtitle and the text. At line 272 we added a space, and the text “Based on the data gleaned”, now starts at line 273 in the manuscript without the “Track changes” option active. The change (the space added) can be seen at line 366 in the revised manuscript with the option “Track changes” and “All Markup” active, and at line 272 in the manuscript without the “Track changes” option active.

**Reviewer 1 comment:**

Line 293 the data was analyzed

Minor formatting concern-should be another space between subtitle and text.

**Response:**

Thank you for the recommendation. We added a space between the subtitle and the text. Line 293 became line 294 in the manuscript without the “Track changes” option active, because of previous changes. Thus, we added a space at line 294 and the text “The data was analyzed” now starts at line 295. The change (the space added) can be seen at line 403 in the revised manuscript with the option “Track changes” and “All Markup” active, and at line 295 in the manuscript without the “Track changes” option active.

**Reviewer 1 comment:**

Line 354 the means (Table 2) indicate that most

Minor formatting concern-should be another space between subtitle and text.

**Response:**

We added a space between the subtitle and the text. Line 354 became line 356 in the manuscript without the “Track changes” option active, because of previous changes. Thus, we added a space at line 356 and the text “The means (Table 2) indicate that most” now starts at line 357. The change (the space added) can be seen at line 479 in the revised manuscript with the option “Track changes” and “All Markup” active, and at line 357 in the manuscript without the “Track changes” option active.

**Reviewer 1 comment:**

Line 418 in consideration to the use of social

Minor formatting concern-should be another space between subtitle and text.

**Response:**

We added a space between the subtitle and the text. Line 418 became line 421 in the manuscript without the “Track changes” option active, because of previous changes. Thus, we added a space at line 421 and the text “In consideration to the use of social” now starts at line 422. The change (the space added) can be seen at line 552 in the revised manuscript with the option “Track changes” and “All Markup” active, and at line 421 in the manuscript without the “Track changes” option active.

**Reviewer 1 comment:**

Line 443 in consideration to the use of Facebook

Minor formatting concern-should be another space between subtitle and text.

**Response:**

We added a space between the subtitle and the text. Line 443 became line 447 in the manuscript without the “Track changes” option active, because of previous changes. Thus, we added a space at line 447 and the text “In consideration to the use of Facebook” now starts at line 448. The change (the space added) can be seen at line 585 in the revised manuscript with the option “Track changes” and “All Markup” active, and at line 447 in the manuscript without the “Track changes” option active.

**Reviewer 1 comment:**

Line 462 Previous studies on social networks use

Minor formatting concern-should be another space between subtitle and text.

**Response:**

We added a space between the subtitle and the text. Line 462 became line 467 in the manuscript without the “Track changes” option active, because of previous changes. Thus, we added a space at line 467 and the text “In consideration to the use of Facebook” now starts at line 468. The change (the space added) can be seen at line 613 in the revised manuscript with the option “Track changes” and “All Markup” active, and at line 467 in the manuscript without the “Track changes” option active.

**Reviewer 1 comment:**

Line 472 research that could complement and come as a response to a similar study

Could complement and act as a response to a similar study

**Response:**

We changed the text according to the recommendation. Line 472 became line 478 in the manuscript without the “Track changes” option active. The change can be seen at lines 628 - 630 in the revised manuscript with the option “Track changes” and “All Markup” active, and at line 478 in the manuscript without the “Track changes” option active.

**Reviewer 1 comment:**

Line 474 research that could come as a response to a previous study

Could support a previous study

Also, more information about this previous study. Who conducted it? For what purpose? What were the results?

**Response:**

We changed the text according to the recommendation. Line 474 became line 480 in the manuscript without the “Track changes” option active. The change can be seen at line 653 in the revised manuscript with the option “Track changes” and “All Markup” active, and at line 480 in the manuscript without the “Track changes” option active. In order to improve the article, as the reviewer suggested we added some more information about the previous study. The study was conducted by some of the researchers involved in this present study, and it addressed the subject of students’ motives for using Facebook, while focusing on the use of the platform for academic purposes. Hence, at lines 635 - 641 in the revised manuscript with the option “Track changes” and “All Markup” active, and at lines 483 - 489 in the manuscript without the “Track changes” option active, the following text was added:

“Even more, in the study were involved some of the researchers involved in the present study, and the purpose of the study was to identify how students perceive the use of Facebook for academic purposes. The results of the study revealed that more than half of the respondents (57.3%) were open to receiving tasks and projects through messages sent through Facebook, and that 30% of the respondents considered that Facebook can represent a comfortable environment that could also increase their motivation to research, discover or complete school projects.”

**Reviewer 1 comment:**

Line 485 Even more, to highlight why we focused only on Facebook and Instagram, and not on other 486 platforms such as Yoututbe

Further, we focused solely on Facebook and Instagram…also should be YouTube

**Response:**

We changed the text according to the recommendation. Line 485 became line 497 in the manuscript without the “Track changes” option active. In order not to lose the meaning of the sentence, we changed the first part of sentence as the reviewer recommended, but we kept the second part of the sentence: “Further, we focused solely on Facebook and Instagram and not on other platforms such as YouTutbe, Tik Tok, Steemit, Researchgate, LinkedIN, Skillshare or Coursera, and we further present several reasons for this.” The change can be seen at lines 649 - 651 in the revised manuscript with the option “Track changes” and “All Markup” active, and at lines 497 - 499 in the manuscript without the “Track changes” option active.

**Reviewer 1 comment:**

Line 487 YouTube

**Response:**

We corrected the word. Line 487 became line 500 in the manuscript without the “Track changes” option active. The change can be seen at lines 651 in the revised manuscript with the option “Track changes” and “All Markup” active, and at line 500 in the manuscript without the “Track changes” option active.

**Reviewer 1 comment:**

Line 489 with diversified content, we considered

With diversified content. As a consequence, we considered….

**Response:**

We changed the text according to the recommendation. Line 489 became line 501 in the manuscript without the “Track changes” option active. The change can be seen at line 653 in the revised manuscript with the option “Track changes” and “All Markup” active, and at lines 501- 502 in the manuscript without the “Track changes” option active.

**Reviewer 1 comment:**

Line 491 between students. As far as Tik Tok is concerned, being a relatively new platform, we 492 considered that the analysis of the way it could be integrated into the educational process, 493 could have altered the replicability of the research, because the development and use of Tik 25 494 Tok globally is rather uneven.

In regard to Tik Tok, while we considered the educational benefits of the platform, we decided to exclude it from the study because it is a relatively new platform and people’s response to the application is uneven

**Response:**

We are very grateful for the recommendation. We changed and replaced the text according to the recommendation. Line 491 became line 501 in the manuscript without the “Track changes” option active. The change can be seen at lines 655 - 657 in the revised manuscript with the option “Track changes” and “All Markup” active, and at lines 504- 506 in the manuscript without the “Track changes” option active.

**Reviewer 1 comment:**

Line 500 Researchgate is a platform that aims to 500 promote research, to spread knowledge through scientific papers, and we wanted to focus on 501 social networks that offer more diverse functions.

While Researchgate is a beneficial platform for promoting research and spreading knowledge trough the scientific community, it is not a widely known application with diverse functions.

**Response:**

Thank you very much for the suggestion. We replaced the text with the text recommended. Line 500 became line 511 in the manuscript without the “Track changes” option active. The change can be seen at lines 663 – 665 in the revised manuscript with the option “Track changes” and “All Markup” active, and at lines 511- 513 in the manuscript without the “Track changes” option active.

**Reviewer 1 comment:**

Line 502 that could be used by students and teachers for searching for jobs and internships, but our 503 interest was in finding out how social networks more general and comprehensive,

Internships; however, our interest

**Response:**

Thank you for the suggestion. We changed the text according to the suggestion. Line 502 became line 514 in the manuscript without the “Track changes” option active. The change can be seen at line 666 in the revised manuscript with the option “Track changes” and “All Markup” active, and at lines 514- 515 in the manuscript without the “Track changes” option active.

**Reviewer 1 comment:**

Line 505 Furthermore, Coursersa was not in 505 our area of interest because the focus of this paper was on social networks, on platforms that 506 were not initially designed for educational purposes

Finally, Coursersa was not a focus of this particular study because it centralized its format specifically on educational purposes, rather than social network outreach.

**Response:**

Thank you for the recommendation. We changed the text according to the recommendation. Line 505 became line 516 in the manuscript without the “Track changes” option active. The change can be seen at lines 668 - 669 in the revised manuscript with the option “Track changes” and “All Markup” active, and at lines 516- 518 in the manuscript without the “Track changes” option active.

**Reviewer 1 comment:**

Line 510 they allow students to properly communicate and collaborate with eachother during courses

Each other

**Response:**

We corrected the word. Line 510 became line 522 in the manuscript without the “Track changes” option active. The change can be seen at line 673 in the revised manuscript with the option “Track changes” and “All Markup” active, and at line 522 in the manuscript without the “Track changes” option active.

**Reviewer 1 comment:**

Line 511 for example, on Facebook, teachers could create groups and students could work in teams in 512 order to fulfil certain tasks, that can later be presented to the other teams on a live meeting)

Separate sentence entirely

**Response:**

Thank you for the suggestion. We separated the sentence. Line 511 became line 523 in the manuscript without the “Track changes” option active. The change can be seen at lines 673 - 675in the revised manuscript with the option “Track changes” and “All Markup” active, and at lines 523 - 524 in the manuscript without the “Track changes” option active.

**Reviewer 1 comment:**

Line 513 and last but not least, students are familiar with these social networks, and even though they 514 usually use them to relax and communicate with their friends, because they are already 26 515 accustomed to them, students may be more relaxed, more creative and more willing to learn in 516 a familiar environment.

Separate sentence entirely

**Response:**

Thank you for the suggestion. We separated the sentence. Line 513 became line 525 in the manuscript without the “Track changes” option active. The change can be seen at lines 676 – 679 in the revised manuscript with the option “Track changes” and “All Markup” active, and at lines 525 - 528 in the manuscript without the “Track changes” option active. In order to comply with the recommendation, the text was changed in the following way:

“Last but not least, students are familiar with these social networks. Even though they usually use them to relax and communicate with their friends, because they are already accustomed to them, students may be more relaxed, more creative and more willing to learn in a familiar environment.”

**Reviewer 1 comment:**

Line 519 used to promote knowledge in higher education, but only 26,7%

26.7%

**Response:**

We changed the text according to the recommendation. Line 519 became line 531 in the manuscript without the “Track changes” option active. The change can be seen at line 687 in the revised manuscript with the option “Track changes” and “All Markup” active, and at line 531 in the manuscript without the “Track changes” option active.

**Reviewer 1 comment:**

Line 536 educational process, students would rather use Facebook. Thus,

You use “thus” too frequently. You can use “as a consequence” instead.

**Response:**

We changed the text according to the recommendation. Line 536 became line 548 in the manuscript without the “Track changes” option active. The change can be seen at line 705 in the revised manuscript with the option “Track changes” and “All Markup” active, and at line 548 in the manuscript without the “Track changes” option active.

**Reviewer 1 comment:**

Line 535 According to our findings, if they were to use Facebook and Instagram in the 536 educational process, students would rather use Facebook. Thus, this higher preference for 27 537 Facebook, and the idea that Facebook is the most suitable platforms for students, might also be 538 influenced by the fact that the platform was initially created for students [61], in order to help 539 them socialize. A

Very choppy. Consider re-wording these sentences.

For example, “according to our findings, students prefer using Facebook exclusively for educational purposes, opting to exclude the use of Instagram. Because students prefer Facebook, and because it is better suited for students academically, this platform may aid instructors in student socialization and interaction.

Another aspect revealed by our research is that master’s and Ph.D. students are 540 more willing to use Facebook for educational purposes than, Bachelor students. Thus, this 541 result is in line with other studies conducted in Romania, [8] which showed that Master 542 students were fonder of using online platforms, than Bachelor students.

“our study also revealed acceptance of these social platforms by master’s and Ph.D. students, which concurs with previous studies conducted in Romania, which supported the use of online platforms in students with advanced educational degrees as opposed to Bachelor’s students.”

**Response:**

We are very grateful for the suggestion. We changed the text according to the recommendation.

Line 535 became line 547 in the manuscript without the “Track changes” option active. We deleted the text and inserted the text suggested by the reviewer. The text deleted and the text inserted can be viewed at lines 704 - 711 in the revised manuscript with the option “Track changes” and “All Markup”, and in the manuscript without the “Track changes” option active, the text that we added can be seen at lines 547 – 550.

We would like to mention that, since the phrase was re - worded, the change made to the text in order to comply with the previous comment of the reviewer, according to which we had to change the word “thus” from line 536 which became line 548, to “as a consequence”, can no longer be seen.

In regard to the second sentence that needed to be re – worded, we firstly mention that lines 539 - 540 became lines 550 – 551 in the manuscript without the “Track changes” option active. We deleted the text and inserted the text suggested by the reviewer. The full change can be seen at lines 711 – 718 in the revised manuscript with the option “Track changes” and “All Markup”, and in the manuscript without the “Track changes” option active, the text that we added can be seen at lines 550 - 553.

**Reviewer 1 comment:**

Line 548 process. 549 In the case of Instagram, i

Combine these paragraphs

**Response:**

Thank you for the suggestion. We combined the paragraphs. Lines 548 - 549 became lines 559 - 560 in the manuscript without the “Track changes” option active. The change can be seen at line 727 in the revised manuscript with the option “Track changes” and “All Markup” active, and at line 559 in the manuscript without the “Track changes” option active.

**Reviewer 1 comment:**

Line 551 students who use daily their Facebook and Instagram accounts

Students who use their Facebook and Instagram accounts daily

**Response:**

We changed the text according to the recommendation. Line 551 became line 561 in the manuscript without the “Track changes” option active. The change can be seen at lines 732 – 733 in the revised manuscript with the option “Track changes” and “All Markup” active, and at lines 561 - 562 in the manuscript without the “Track changes” option active.

**Reviewer 1 comment:**

Line 557 such as Facebook and Instagram is relevant and necessary. Thus

Again, thus

**Response:**

We replaced to word “thus” with “hence”. Line 557 became line 567 in the manuscript without the “Track changes” option active. The change can be seen at line 740 in the revised manuscript with the option “Track changes” and “All Markup” active, and at line 567 in the manuscript without the “Track changes” option active.

**Reviewer 1 comment:**

Line 569 respondents daily used Facebook for more than 4,5 hours

4.5 hours

**Response:**

We replaced to comma, “,” with “.” Line 569 became line 579 in the manuscript without the “Track changes” option active. The change can be seen at line 752 in the revised manuscript with the option “Track changes” and “All Markup” active, and at line 579 in the manuscript without the “Track changes” option active.

**Reviewer 1 comment:**

Line 584 Taking into consideration the aspects mentioned above, certain issues can be 585 addressed. The first issue refers to whether Moodle platforms

Not all educators are versed in Moodle. You should define this platform

**Response:**

Line 584 became line 594 in the manuscript without the “Track changes” option active. In order to comply with the suggestion, we addressed the point raised by the reviewer, and we explained that, even though not all teachers have the necessary skills in order to use Moodle platforms, by organizing certain trainings or courses for them, or by providing certain guides in which each of the functions of the platform is explained, teacher could better understand how to use such platforms. In this regard, at lines 774 – 780 in the revised manuscript with the option “Track changes” and “All Markup” active, and at lines 601 - 607 in the manuscript without the “Track changes” option active, the following text was added:

“Even more, another issue related to the use of Moodle platforms in the educational process is represented by the fact that not all educators have the necessary skills in order to properly use the platforms. However, in order to help teachers understand how to use such platforms in order to better collaborate with students, universities could organize certain training sessions for teachers, and they could offer teachers access to detailed guides that provide explanation on the way the tools integrated into the platform should be used.”

**Reviewer 1 comment:**

Line 594 these platforms only because they would have a reasonable motive for using them, thus not 595 developing a feeling of guilt for not studying. Thus, a

Thus, again

**Response:**

We replaced to word “Thus” with “In this regard”. Line 594 became line 611 in the manuscript without the “Track changes” option active. The change can be seen at line 784 in the revised manuscript with the option “Track changes” and “All Markup” active, and at line 611 in the manuscript without the “Track changes” option active.

**Reviewer 1 comment:**

Line 606 In the context

Minor formatting concern-should be another space between subtitle and text.

**Response:**

We added a space between the subtitle and the text. Line 606 became line 622 in the manuscript without the “Track changes” option active, because of previous changes. Thus, we added a space at line 622 and the text “In the context..” now starts at line 623. The change (the space added) can be seen at line 795 in the revised manuscript with the option “Track changes” and “All Markup” active, and at line 622 in the manuscript without the “Track changes” option active.

**Reviewer 1 comment:**

Line 629 Taking into account the previously mentioned aspects, some theoretical and practical 630 implications of our paper can be highlighted. Considering the

Awkward sentence structure. Consider something like “turning to the theoretical and practical implications of our paper, this research can contribute to the literature

**Response:**

We changed the text according to the recommendation. Line 629 became line 646 in the manuscript without the “Track changes” option active. The change (the text we deleted and the text we inserted) can be seen at lines 819 - 822 in the revised manuscript with the option “Track changes” and “All Markup” active, and in the manuscript without the “Track changes” option active, only the text inserted can be seen at lines 646 - 647.

**Reviewer 1 comment:**

Line 639 Therefore

Minor formatting concern-should be another space between subtitle and text.

Also, delete therefore

**Response:**

We deleted the word “Therefore.” We added a space between the subtitle and the text. Line 639 became line 655 in the manuscript without the “Track changes” option active, because of previous changes. Thus, we added a space at line 655 and the text “The study..” now starts at line 656. The change (the space added) can be seen at line 830 in the revised manuscript with the option “Track changes” and “All Markup” active, and at line 656 in the manuscript without the “Track changes” option active.

**Reviewer 1 comment:**

Line 640 future researches. Our study being exploratory

Because our study is exploratory, the instruments

**Response:**

We changed the text according to the recommendation. Line 640 became line 657 in the manuscript without the “Track changes” option active. The change (the text we deleted and the text we inserted) can be seen at line 832 in the revised manuscript with the option “Track changes” and “All Markup” active, and in the manuscript without the “Track changes” option active, only the text inserted can be seen at line 657.

We thank you again for the time spent reviewing our article and for the useful recommendations provided!

**Our response to Reviewer 2:**

We are very grateful to the reviewer for taking the times to review our paper and to examine the way we addressed his/hers previous comments. We complied with the suggestion made by the reviewer, and the changes to the text were made while having active the option “Track changes” and “All Markup” from Microsoft Word.

**Reviewer 2 comment:**

I think the authors made an excellent work in addressing all the comments.

I only have one final suggestion: I understand Steemit is not as popular as other platforms, so maybe a citation that explains its features is due to help the reader in case she/he wants to to delve into the platform. Just add the following reference the first time you name Steemit in your paper: <https://ieeexplore.ieee.org/document/9298888/>

**Response:**

Thank you for the suggestion. We inserted into our manuscript the reference provided. Since the reviewer suggested that the reference should be added the first time we mentioned the platform Steemit, we added it at line 498 in the manuscript without the “Track changes” option active, which is line 650 in the revised manuscript with the option “Track changes” and “All Markup” active.

The reference has the number [86]: Guidi B, Michienzi A, Ricci L. A Graph-Based Socioeconomic Analysis of Steemit. IEEE Transactions on Computational Social Systems. 2020; 8 (2): 365-376

doi: 10.1109/TCSS.2020.3042745.

In the reference list, it can be found at lines 936 – 938 in the manuscript without the “Track changes” option active, which are lines 1139 – 1141, in the revised manuscript with the option “Track changes” and “All Markup” active.

We thank you again for all the recommendations provided in order to improve our article!

Further, we present our response to the points raised by the academic editor in order to meet the requirements of the journal.

**Journal requirements:**

Please review your reference list to ensure that it is complete and correct. If you have cited papers that have been retracted, please include the rationale for doing so in the manuscript text, or remove these references and replace them with relevant current references. Any changes to the reference list should be mentioned in the rebuttal letter that accompanies your revised manuscript. If you need to cite a retracted article, indicate the article’s retracted status in the References list and also include a citation and full reference for the retraction notice.

**Response:**

We reviewed our reference list and made sure it is correct and complete. We did not cite papers that were retracted. The only change made to the reference list was adding another reference to our list, because Reviewer 2 recommended us to add it. The reference added has the number 86: Guidi B, Michienzi A, Ricci L. A Graph-Based Socioeconomic Analysis of Steemit. IEEE Transactions on Computational Social Systems. 2020; 8 (2): 365-376

doi: 10.1109/TCSS.2020.3042745.

In the text we added the reference at line 498 in the manuscript without the “Track changes” option active, which is line 650 in the revised manuscript with the option “Track changes” and “All Markup” active, and in the reference list, it can be seen at lines 936 – 938 in the manuscript without the “Track changes” option active, which are lines 1139 – 1141, in the revised manuscript with the option “Track changes” and “All Markup” active.

We are very thankful to the reviewers and the academic editor for all the useful recommendations, comments and points raised in order to improve our paper!

Sincerely,

Prof. Dr. Claudiu Coman
